# Therapeutic potential of berries in age-related neurological disorders

**DOI:** 10.3389/fphar.2024.1348127

**Published:** 2024-05-09

**Authors:** Narges Norouzkhani, Shaghayegh Afshari, Sayedeh-Fatemeh Sadatmadani, Mohammad Mahdi Mollaqasem, Shakila Mosadeghi, Hani Ghadri, Safa Fazlizade, Keyvan Alizadeh, Pouyan Akbari Javar, Hamidreza Amiri, Elaheh Foroughi, Arina Ansari, Kourosh Mousazadeh, Bozorgmehr Abdullahzadeh Davany, Ata Akhtari kohnehshahri, Alaleh Alizadeh, Parisa Alsadat Dadkhah, Mohadeseh Poudineh

**Affiliations:** ^1^ Department of Medical Informatics, Faculty of Medicine, Mashhad University of Medical Sciences, Mashhad, Iran; ^2^ Student Research Committee, School of Medicine, Shahroud University of Medical Sciences, Shahroud, Iran; ^3^ School of Medicine, Isfahan University of Medical Sciences, Isfahan, Iran; ^4^ Student Research Committee, School of Medicine, Iran University of Medical Sciences, Tehran, Iran; ^5^ Student Research Committee, School of Medicine, Ardabil University of Medical Sciences, Ardabil, Iran; ^6^ Student Research Committee, School of Medicine, Shahid Beheshti University of Medical Science, Tehran, Iran; ^7^ Student Research Committee, School of Medicine, Arak University of Medical Sciences, Arak, Iran; ^8^ Student Research Committee, School of Medicine, North Khorasan University of Medical Sciences, Bojnurd, Iran; ^9^ School of Medicine, Islamic Azad University, Tehran Medical Branch, Tehran, Iran; ^10^ Student Research Committee, School of Medicine, Islamic Azad University, Tehran Medical Branch, Tehran, Iran; ^11^ Student Research Committee, Faculty of Medicine, Tabriz Medical Sciences, Islamic Azad University, Tabriz, Iran; ^12^ Student Research Committee, Faculty of Medicine, Mashhad Branch, Islamic Azad University, Mashhad, Iran; ^13^ Student Research Committee, School of Medicine, Isfahan University of Medical Sciences, Isfahan, Iran; ^14^ Student Research Committee, School of Medicine, Zanjan University of Medical Sciences, Zanjan, Iran

**Keywords:** berry fruits, age-related neurological disease, aging, complementary medicine, treatment

## Abstract

Aging significantly impacts several age-related neurological problems, such as stroke, brain tumors, oxidative stress, neurodegenerative diseases (Alzheimer’s, Parkinson’s, and dementia), neuroinflammation, and neurotoxicity. Current treatments for these conditions often come with side effects like hallucinations, dyskinesia, nausea, diarrhea, and gastrointestinal distress. Given the widespread availability and cultural acceptance of natural remedies, research is exploring the potential effectiveness of plants in common medicines. The ancient medical system used many botanical drugs and medicinal plants to treat a wide range of diseases, including age-related neurological problems. According to current clinical investigations, berries improve motor and cognitive functions and protect against age-related neurodegenerative diseases. Additionally, berries may influence signaling pathways critical to neurotransmission, cell survival, inflammation regulation, and neuroplasticity. The abundance of phytochemicals in berries is believed to contribute to these potentially neuroprotective effects. This review aimed to explore the potential benefits of berries as a source of natural neuroprotective agents for age-related neurological disorders.

## 1 Introduction

Humans have always had an innate urge to protect their chances of survival and to age gracefully or become eternal. As a result of recent advances in medical science and medical technology, the average human lifespan has increased. Unfortunately, these advances have not yet produced the anticipated improvement in healthy aging (Campisi et al., 2019). Aging is a physiological process that leads to irreversible and progressive organ and tissue deterioration. It is mediated by various genetic markers, which are directly associated with longevity and all age-related diseases (Harman, 2001). Neurodegenerative diseases, cancers, cardiovascular diseases, immunological disorders, musculoskeletal disorders and metabolic diseases are the most prevalent age-related illnesses (Li et al., 2021). Age is the most significant threat factor for age-related neurological diseases (ANDs), particularly Alzheimer’s disease (AD), Parkinson’s disease (PD), Cerebrovascular accident (CVA) and other cognitive impairments. A wealth of literature has shown significant adverse effects of aging on the neurological system (Pringsheim et al., 2014; Siebert et al., 2018; Hou et al., 2019; Trevisan et al., 2019; Yousufuddin and Young, 2019). Deoxyribonucleic acid (DNA) damage, which worsens with age, plays a crucial role in the pathogenesis of ANDs. The brain is particularly vulnerable to the harmful effects of aging and is more exposed to DNA damage than other organs since it is mainly made up of post-mitotic cells (Madabhushi et al., 2014). The pathogenesis of ANDs is strongly associated with some natural markers, including genomic instability, short telomeres, epigenetic variations, loss of proteostasis, altered intercellular communication, deregulated nutrient seeing, cellular anility, stem cell prostration, and mitochondrial dysfunction, which are the main targets of the anti-aging therapy (López-Otín et al., 2013).

Population aging is an undeniable consequence of modern demographics, leading to a concerning rise in mortality and disability rates. This trend poses a significant social, psychological, and economic burden on societies worldwide (De Magalhães et al., 2017). The lack of readily available, efficacious treatments or preventive measures for age-related diseases further exacerbates the challenge, resulting in escalating healthcare costs. Therefore, exploring novel approaches to promote healthy aging is critical. In this context, dietary interventions, particularly the inclusion of fruits, have emerged as a promising strategy (Paredes-López et al., 2010; Navarro-Hortal et al., 2022). Nutritional guidelines consistently emphasize the importance of a balanced diet rich in fruits. Emerging research suggests that specific metabolites of certain berries may play a role in delaying the onset of ANDs (Navarro-Hortal et al., 2022). Berries, encompassing popular varieties from the *Rosaceae* J*uss*. (strawberry (*Fragaria vesca* L.), blackberry (*Rubus fruticosus* L.), and jeer), *Ericaceae* Durande (blueberry (*Vaccinium corymbosum* L.) and cranberry (*Vaccinium macrocarpon* Aiton)), and *Vitis vinifera L* (grape) families, are a valuable source of bioactive metabolites linked to potential health benefits (Skrovankova et al., 2015a). These bioactive metabolites encompass antioxidants like phenolic metabolites, anthocyanins, and carotenoids, alongside essential vitamins such as folic acid and ascorbic acid (Skrovankova et al., 2015b). The aforementioned bioactive elements contribute to the potential of berries as a functional food. Studies suggest that the consumption of blueberries, either whole fruit, leaves, or even supplements, might be beneficial in preventing neurological disorders with a neuroinflammatory metabolite.

Studies suggest that antioxidant-rich berry fruit supplements may offer a promising alternative for managing conditions like anxiety, depression, Parkinson’s disease, and Alzheimer’s disease (Olas, 2018; Micek et al., 2022). These findings point towards specific phenolic metabolites, such as quercetin and rutin, present in berry extracts potentially triggering anti-inflammatory responses through the modulation of oxidative stress (Khan et al., 2023). Phenolic acids, including conjugates of hydroxybenzoic and hydroxycinnamic acids, and flavonoids (encompassing flavonols, anthocyanins, stilbenes, and tannins) are key contributors to the vibrant colors of berries (red, violet, and blue) and are believed to play a crucial role in their health benefits (Beattie et al., 2005; Paredes-López et al., 2010; Skrovankova et al., 2015b). Berry consumption may significantly promote healthy aging by mitigating oxidative stress, mitochondrial dysfunction, neuroinflammation, and potentially influencing factors like genomic stability, telomere degradation, cellular senescence, and nutrient uptake (Maher, 2017; Maher, 2019a; Navarro-Hortal et al., 2022). Age-related factors often lead to an overproduction of free radicals and reactive oxygen species (ROS), overwhelming the body’s antioxidant defenses and contributing to neuroinflammation (Paredes-López et al., 2010). Given the potential of berries’ bioactive metabolite to contribute to healthy aging, this review aims to evaluate the scientific evidence supporting the potential of berry consumption or berry-derived supplements for promoting healthy aging and mitigating ANDs.

## 2 Materials and methods

The information regarding the pharmacotherapeutic potential of berries in age-related neurological clutters is summarized in this study. The keywords used in this analysis were (“*Vaccinium* L.”, “blueberries”, “*R. fruticosus* L.”, “blackberries”, “*Rubus idaeus* L.”, “raspberries”, “*Vaccinium macrocarpon*”, “cranberries”, “Strawberries”, “Huckleberry”, “Chokeberry”, “Aronia berry”, “Elderberry”, “Gooseberry”, “Lingonberry”, “Boysenberries”, “Mulberry”, “ethanol extract of mulberry”, “Anthocyanin”, “cyanidin-3-O-glucoside”, “Ellagic acid”, “Tetrahydroxybenzopyrano”, “3-Hydroxybenzofuran”, “benzopyran dione”, “phenolicacids”, “flavonoids”, “tannins”, “flavonols”, “ascorbic acid”, “proanthocyanin”, “Hydroxycinnamic acids”, “hydroxycinnamates”, “Gallic acid”) AND (“Oxidative Stress”, “Alzheimer Disease”, “Alzheimer’s disease”, “Parkinson’s disease”, “Parkinson Disease”, “dementia”, “polyneuropathy*”, “Neurotoxicity”, “Neurotoxicity Syndrome*", “Neuroinflammation”, “Neuroinflammatory Disease*" “brain tumor”, “Brain Neoplasm*", “migraine “, “Migraine Disorder*", “Epilepsy”, “Multiple sclerosis”, “Atherosclerosis”, “Glioma”, “Gliosarcoma”, “Cerebrovascular Disorders”, “Huntington Disease”, “Memory loss”, “Memory Disorder*"). Searches for English-language journal articles published up to 30 September 2022 were conducted in databases including PubMed/MEDLINE, Scopus and Google Scholar. According to each database, we used particular phrases and approaches (See Table 1 for the search strategy).

**TABLE 1:** Database search strategies.

Search engine	Search strategy	Results
PubMed/MEDLINE	(“Vaccinium cyanococcus” [ti] OR “blueberries” [ti] OR “Rubus fruticosus” [ti] OR “blackberries” [ti] OR “Rubus idaeus” [ti] OR “raspberries” [ti] OR “Vaccinium macrocarpon” [ti] OR “cranberries” [ti] OR “Strawberries” [ti] OR “Huckleberry” [ti] OR “Chokeberry” [ti] OR “Aronia berry” [ti] OR “Elderberry” [ti] OR “Gooseberry” [ti] OR “Lingonberry” [ti] OR “Boysenberries” [ti] OR “Mulberry” [ti] OR “ethanol extract of mulberry” [ti] OR “Anthocyanin” [ti] OR “cyanidin-3-O-glucoside” [ti] OR “Ellagic acid” [ti] OR “Tetrahydroxybenzopyrano” [ti] OR “3-Hydroxybenzofuran” [ti] OR “benzopyran-dione” [ti] OR “phenolic acids” [ti] OR “flavonoids” [ti] OR “tannins” [ti] OR “flavonols” [ti] OR “ascorbic acid” [ti] OR “proanthocyanin” [ti] OR “Hydroxycinnamic acids” [ti] OR “hydroxycinnamates” [ti] OR “Gallic acid” [ti])	124
	AND (“Oxidative Stress” [ti] OR “Oxidative Stress" [Mesh] OR “Alzheimer Disease" [ti] OR “Alzheimer Disease” [Mesh] OR “Alzheimer’s disease” [ti] OR “Parkinson’s disease” [ti] OR “Parkinson Disease” [ti] OR “Parkinson Disease” [Mesh] OR “dementia” [ti] OR “Dementia" [Mesh] OR “polyneuropathy*“ [ti] OR “Polyneuropathies” [Mesh] OR “Neurotoxicity” [ti] OR “Neurotoxicity Syndrom*" [ti] OR “Neurotoxicity Syndromes" [Mesh] OR “Neuroinflammation” [ti] OR “Neuroinflammatory Disease*" [ti] OR “Neuroinflammatory Diseases" [Mesh] OR “brain tumor” [ti] OR “Brain Neoplasm*" [ti] OR “Brain Neoplasms" [Mesh] OR “migraine “[ti] OR “Migraine Disorder*" [ti] OR “Migraine Disorders" [Mesh] OR Epilepsy [ti] OR “Epilepsy" [Mesh] OR “Multiple sclerosis” [ti] OR “Multiple Sclerosis" [Mesh] OR “Atherosclerosis" [ti] OR “Atherosclerosis" [Mesh] OR “Glioma" [ti] OR “Glioma" [Mesh] “Gliosarcoma" [ti] OR “Gliosarcoma" [Mesh] OR “Cerebrovascular Disorders" [ti] OR “Cerebrovascular Disorders" [Mesh] OR “Huntington Disease" [ti] OR “Huntington Disease" [Mesh] OR “Memory loss” [ti] OR “Memory Disorder*" [ti] OR “Memory Disorders" [Mesh])	
	AND 2000/01/01:2022/09/31 [dp]	
Scopus	TITLE (“vaccinium planococcus” OR “blueberries” OR “Rubus fruticosus” OR “blackberries” OR “Rubus idaeus” OR “raspberries” OR “Vaccinium macrocarpon” OR “cranberries” OR “Strawberries” OR “Huckleberry” OR “Chokeberry” OR “Aronia berry” OR “Elderberry” OR “Gooseberry” OR “Lingonberry” OR “Boysenberries” OR “Mulberry” OR “ethanol extract of mulberry” OR “Anthocyanin” OR “cyanidin-3-O-glucoside” OR “Ellagic acid” OR “Tetrahydroxybenzopyrano” OR “3-Hydroxybenzofuran” OR “benzopyran-dione” OR “phenolic acids” OR “flavonoids” OR “tannins” OR “flavonols” OR “ascorbic acid” OR “proanthocyanin” OR “Hydroxycinnamic acids” OR “hydroxycinnamates” OR “Gallic acid")	5
	AND TITLE (“Oxidative Stress” OR “Alzheimer Disease” OR “Alzheimer’s disease” OR “Parkinson’s disease” OR “Parkinson Disease” OR “dementia” OR “polyneuropathy*” OR “Neurotoxicity” OR “Neurotoxicity Syndrome*" OR “Neuroinflammation” OR “Neuroinflammatory Disease*" “brain tumor” OR “Brain Neoplasm*" OR “migraine “OR “Migraine Disorder*" OR “Epilepsy” OR “Multiple sclerosis” OR “Atherosclerosis” OR “Glioma” OR “Gliosarcoma” OR “Cerebrovascular Disorders” OR “Huntington Disease” OR “Memory loss” OR “Memory Disorder*")	
	AND ((PUBYEAR >1999 AND PUBYEAR <2023) AND NOT PUBDATETXT ("September 2022″OR “October 2022″OR “November 2022″OR “December 2022″))	
Google scholar	Intitle: (“vaccinium planococcus” OR “blueberries” OR “Rubus fruticosus” OR “blackberries” OR “Rubus idaeus” OR “raspberries” OR “Vaccinium macrocarpon” OR “cranberries” OR “Strawberries” OR “Huckleberry” OR “Chokeberry” OR “Aronia berry” OR “Elderberry” OR “Gooseberry” OR “Lingonberry” OR “Boysenberries” OR “Mulberry” OR “ethanol extract of mulberry” OR “Anthocyanin” OR “cyanidin-3-O-glucoside” OR “Ellagic acid” OR “Tetrahydroxybenzopyrano” OR “3-Hydroxybenzofuran” OR “benzopyran-dione” OR “phenolic acids” OR “flavonoids” OR “tannins” OR “flavonols” OR “ascorbic acid” OR “proanthocyanin” OR “Hydroxycinnamic acids” OR “hydroxycinnamates” OR “Gallic acid")	1
	AND Intitle: (“Oxidative Stress” OR “Alzheimer Disease” OR “Alzheimer’s disease” OR “Parkinson’s disease” OR “Parkinson Disease” OR “dementia” OR “polyneuropathy*” OR “Neurotoxicity” OR “Neurotoxicity Syndrome*” OR “Neuroinflammation” OR “Neuroinflammatory Disease*" “brain tumor” OR “Brain Neoplasm*” OR “migraine” OR “Migraine Disorder*” OR Epilepsy OR “Multiple sclerosis” OR “Atherosclerosis” OR “Glioma” OR “Gliosarcoma” OR “Cerebrovascular Disorders” OR “Huntington Disease” OR “Memory loss” OR “Memory Disorder*”)	

We have used all the research that has been done on the effects of berries and neurological clutters. The references retrieved were managed with EndNote 8X software and duplicate articles were disregarded. One of the authors, after obtaining the full papers of the research, performed a thorough search of the citations of recovered articles to locate any missing related publications.

## 3 Results

The effects of berries and their active metabolites on age-related neurological problems are discussed in the following paragraphs. The effect of berries on various neurological diorders are summarized in Supplementary Table S2.

### 3.1 Alzheimer’s disease

AD is a genetically complex, age-related neurodegenerative disorder characterized by progressive cognitive decline and irreversible loss of intellectual abilities (Ishola et al., 2019). The global burden of AD is expected to rise significantly, with estimates predicting nearly 1 million new cases annually by 2050 (Qiu et al., 2022; Uwishema et al., 2022). A hallmark feature of AD is oxidative stress, characterized by an imbalance between free radical production and antioxidant defenses, leading to damage of macromolecules and mitochondrial dysfunction. Emerging evidence suggests a potential link between gut microbiota alterations and AD pathogenesis. Changes in gut microbiota composition may contribute to increased amyloid-β aggregation, neuroinflammation, and insulin resistance in the brain, further promoting oxidative stress (Liu et al., 2020; Panchal and Brown, 2022).

While current understanding of AD pathophysiology remains incomplete, mitochondrial dysfunction, insulin resistance, and impaired cerebral blood flow are believed to be key factors mediating neurodegeneration in AD. These pathological processes ultimately contribute to the accumulation of amyloid-β plaques, tau hyperphosphorylation, synaptic loss, and subsequent neurological decline. Intriguingly, research on the microbiota-gut-brain axis has emerged as a promising avenue for exploring novel therapeutic targets for central nervous system (CNS) diseases, including AD (Liu et al., 2020). Studies have shown that specific berry extracts, particularly those rich in blueberry anthocyanins, exhibit various neuroprotective properties against AD. These properties include inhibiting amyloid-β aggregation, preventing glycosylation, scavenging free radicals, and mitigating the effects of reactive carbonyls. Additionally, these extracts may promote microglial neuroprotection (Ma et al., 2018).

### 3.2 Mulberry extract (ME) (Morus alba L.: Moraceae)

Lead (Pb) toxicity is a significant concern due to its detrimental effects on adult learning and memory, particularly in occupational settings (Mason et al., 2014). The high prevalence of Pb exposure further underscores this concern. A study by Mitra et al. found an alarmingly high prevalence of lead poisoning in children residing in metropolitan Bangladesh, with rates ranging from 50% to 99% (Mitra et al., 2012). Notably, even moderate Pb exposure levels in both humans and animals have been associated with various neurological abnormalities (Flora et al., 2007). ME have high antioxidant capacity (Xiangyang Wu et al., 2011). This highlights the potential of Pb exposure to disrupt cognitive function across the lifespan.

A study by Chen et al. investigated the potential protective effects of ME against lead (Pb)-induced neurotoxicity. The study employed a randomized controlled design, dividing mice into eight groups of ten each. Mice were exposed to Pb via oral administration of 500 mg/L Pb acetate for 3 weeks. During this period, different groups received either dimercaptosuccinic acid alone or in combination with varying doses of ME. Notably, the three groups receiving ME intervention displayed a significant decrease in brain lead levels alongside a marked improvement in nitric oxide production and antioxidant enzyme activity. Furthermore, the Morris water maze test revealed a significant improvement in learning and memory function in mice treated with a 100 mg/kg dose of ME. These findings suggest that ME may offer a potential therapeutic strategy for mitigating Pb-induced neurotoxicity. The mechanism by which ME exerts its protective effects, potentially through the reversal of Pb-induced neurotoxic changes, warrants further investigation (Chen et al., 2014).

### 3.3 Ascorbic acid

This study investigated the potential of ascorbic acid (vitamin C) to improve memory function in mice. Male and female Swiss mice at 3 months of age were included. The experimental group received daily oral administration (gavage) of blueberry ethanol extract (AD + blueberry) at a dose of 150 mg/kg body weight for 16 weeks. Control groups included an Alzheimer’s disease model (AD, positive control) and non-transgenic mice (negative control), both receiving saline solution (0.9%) at an equivalent volume. Treatment with ascorbic acid (60 or 120 mg/kg) for 3 or 8 consecutive days significantly improved learning and memory in aged mice. This effect was attributed to reduced transfer quiescence and increased depressive inactivity. Ascorbic acid also protected young mice from memory decline induced by scopolamine and diazepam, suggesting its potential as a memory-enhancing agent. Interestingly, the memory improvement observed in older mice treated with ascorbic acid was more pronounced compared to young mice. This may be related to the primary absorption of ascorbic acid in the body. Previous research on live animals suggests that dietary vitamin E may reduce the risk of developing AD. In contrast, this study demonstrates that ascorbic acid administration for a short duration (3 or 8 days) significantly improves memory in both young and old mice. These findings suggest that while vitamin E may offer preventive benefits against AD, ascorbic acid could potentially be explored as a therapeutic agent for treating AD by targeting amnesia-related mechanisms (Parle and Dhingra, 2003).

### 3.4 Ellagic acid

According to a previous original study utilizing a genetically modified mouse model of AD, mice who consumed blueberry extract showed higher plasticity as seen by improved long-term potentiation, less cell loss in the hippocampus, and a total rise in BDNF (Tan et al., 2017).

A study by Kiasalari et al. investigated the potential of ellagic acid (EA) to counteract learning and memory impairments caused by amyloid beta (Aβ) injection in the hippocampus of rats. In this study, rats were randomly assigned to four groups: a sham group, an ellagic acid-pretreated sham group, an Aβ-injected group, and an Aβ-injected group pretreated with ellagic acid at varying doses (10, 50, or 100 mg/kg). The researchers observed that Aβ injection significantly impaired the rats’ ability to discriminate between novel and familiar objects. However, pretreatment with ellagic acid, particularly at the highest dose (100 mg/kg), significantly improved learning and memory performance in the Aβ-injected rats. Mechanistically, the study suggests that ellagic acid’s protective effects may be mediated by its ability to reduce oxidative stress and acetylcholineesterase activity, both of which are implicated in Aβ-induced neurodegeneration. Furthermore, ellagic acid treatment appeared to regulate the expression of specific proteins (NF-κB, Nrf2, and TLR4) involved in inflammatory and antioxidant pathways within the hippocampus. The most pronounced effects on these proteins were observed at the highest ellagic acid dose (100 mg/kg). Overall, this study provides evidence that ellagic acid treatment may offer a potential therapeutic strategy to mitigate Aβ-induced learning and memory deficits. The findings suggest that ellagic acid’s neuroprotective effects may stem from its ability to modulate oxidative stress, neuroinflammation, and key regulatory proteins (Kiasalari et al., 2017). Further research is needed to explore the optimal dosing regimen and long-term safety of ellagic acid for potential use in AD prevention or treatment.

### 3.5 Flavonoids

Similar to fisetin, quercetin, another flavonol, has shown promise in various animal models of AD (Zaplatic et al., 2019) following either oral administration (Puerta et al., 2017) or IP injection (Sabogal-Guaqueta et al., 2015). Notably, a study using SAMP8 mice compared the effects of free quercetin to nano-encapsulated quercetin patches. Treatment with quercetin nanoparticles resulted in a significant increase in brain quercetin levels, which coincided with improved learning and memory, and reduced astrogliosis (Puerta et al., 2017). Rutin, another dietary flavonoid, has also been investigated for its potential benefits in AD. Studies in rats have shown that rutin administration following Aβ injection can prevent memory loss, minimize lipid peroxidation (cellular damage), and increase levels of brain-derived neurotrophic factor (BDNF), a protein critical for neuronal health. Furthermore, rutin appears to exert neuroprotective effects by inhibiting Aβ aggregation and cytotoxicity (cell death), reducing oxidative stress, and lowering production of pro-inflammatory cytokines and nitric oxide in SH-SY5Y neuroblastoma cells (Moghbelinejad et al., 2014).

Similar to quercetin nanoparticles (Puerta et al., 2017), studies have shown that daily injections of a combination of anthocyanins (a type of flavonoid) with gold nanoparticles (10 mg/kg/day) are significantly more effective than free anthocyanins in reducing memory decline, synaptic protein loss, and neuroinflammation in mice with Aβ1-42 pathology (Ali et al., 2017; Kim et al., 2017). However, translating these findings to humans remains a challenge.

The improved efficacy observed with nanoparticle delivery systems for quercetin (Puerta et al., 2017) and anthocyanins (Ali et al., 2017; Kim et al., 2017) underscores the importance of optimizing delivery methods for potential future pharmacological applications of flavonoids. Current treatments for dementia (including those associated with aging) often focus on inhibiting acetylcholinesterase activity or mitigating neurodegeneration, oxidative stress, and inflammation (Knopman, 2006). Researchers are also investigating how natural product derivatives might affect dementia and Alzheimer’s disease by addressing these same targets (cholinergic deficit, oxidative stress, and inflammation).

Similar to the findings with quercetin nanoparticles, it was determined that daily IP injections of a mixture of anthocyanins, a glycosylated form of anthocyanidin, gold nanoparticles (10 mg/kg/day), were significantly more effective than free anthocyanins in reducing memory impairments, synaptic protein loss, and neuroinflammation in Aβ_1-42_- adapted mouse brains (Ali et al., 2017; Kim et al., 2017). Unfortunately, there is a veritable wealth of research on mortals that it is unclear if this possibility will ever materialize. Additionally, the vastly improved products demonstrated with the quercetin nanoparticles (Puerta et al., 2017) and anthocyanins (Ali et al., 2017; Kim et al., 2017) explosively imply that expression needs to be more carefully considered if flavonoids are to be used pharmacologically. The remedial methods used to treat insanity (whether it be a symptom of similar disorders, or it is due to aging) are based on blocking the acetylcholinesterase enzyme or improving the neuroseditive process and oxidative stress (Knopman, 2006). Researchers are also exploring how natural product derivatives affect craziness and public announcement in light of their potential to correct the cholinergic deficit, oxidative stress, and inflammatory variances.

Recent research suggests that a specific flavonoid, cyanidin-3-O-β-glucopyranoside, may improve cognitive function in AD by increasing levels of PGC-1α, a protein involved in cellular energy metabolism, *in vivo* (Seong et al., 2016). Furthermore, studies have shown that other flavonoids, including dihydromyricetin (Liu et al., 2018), fisetin (Yang W. et al., 2019), icariside II (Yin et al., 2018), curcumin (Li et al., 2015), and genistein (Valles et al., 2010; Bonet-Costa et al., 2016) may also have potential benefits for AD by regulating the AMPK/PGC-1α pathway (Micek et al., 2022). Flavonoids influence neurodevelopmental disorders (NDDs) via the AMPK/PGC-1α pathway, and an antioxidant beverage including polyphenols can reduce total tube homocysteine levels (Liu et al., 2020).

## 4 Amnesia

One of the primary characteristics of AD and one of its earliest and most noticeable symptoms is amnesia (Bertoux et al., 2020). β-Amyloid plaques, neurofibrillary tangles, and a lack of cholinergic neurotransmission are some of the pathologic characteristics of AD (Ishola et al., 2019). Additionally, disruption of the cholinergic system, which may result in memory deficits, is one of the causes of ROS formation (Fukui et al., 2002).

### 4.1 Gallic acid

An *in vivo* study by Nagpal et al. (Nagpal et al., 2013) investigated the potential of gallic acid (GA) to improve memory function in mice with scopolamine-induced amnesia. Scopolamine, a known cholinergic antagonist, disrupts memory and was used to induce amnesia in the study. Male Swiss albino mice were administered either pure GA, GA loaded onto chitosan nanoparticles (GANP) at a dose equivalent to 10 mg/kg GA, or the positive control piracetam (400 mg/kg) for seven consecutive days. Compared to scopolamine-treated mice, those receiving GA displayed significantly improved memory performance. This was evident in the Morris water maze test, where GA-treated mice spent more time in the target quadrant, and in the elevated plus maze test, where they exhibited a reduced transfer latency (time taken to enter a new arm). Importantly, GA treatment also lowered brain acetylcholinesterase activity, an enzyme that breaks down acetylcholine, a key neurotransmitter for memory. These findings suggest that GA’s memory-enhancing effects may be attributed to its ability to inhibit acetylcholinesterase activity. Interestingly, the study found that GA encapsulated in Tween 80-coated nanoparticles (cGANP) showed a more pronounced improvement in memory function compared to free GA. However, there was no significant difference observed between free GA and GANP (GA loaded onto chitosan nanoparticles without the Tween 80 coating). This suggests that the specific nanoparticle formulation (with Tween 80) may play a role in enhancing GA’s bioavailability and delivery to the brain. Overall, this study provides evidence for the potential of GA as a therapeutic strategy for memory impairments associated with Alzheimer’s disease or other cholinergic deficits. Further research is needed to explore the long-term safety and optimal dosing regimen of GA for potential clinical applications.

## 5 Huntington disease

Huntington’s disease is an age-related fatal neurodegenerative disease that presents clinically with progressive psychiatric, motor and cognitive impairment. Huntington’s disease as an autosomal dominant inherited disease manifests after an unstable overreplication of cytosine, adenine and guanine, which encodes an abnormally long polyglutamine tract in the huntingtin protein, and the age at onset of HD is inversely related to the repetition of a number of cytosines, adenine and guanine (Borrell-Pagès et al., 2006; Ramaswamy et al., 2007; Gil and Rego, 2008; Imarisio et al., 2008).

### 5.1 Elderberry

Elderberry (Sambucus nigra L.) possesses a variety of beneficial bioactivities, including antioxidant, anti-inflammatory, and neuroprotective properties (Ferreira et al., 2022; Liu et al., 2022). Studies have shown that elderberry extracts can protect nerve cells from death in animal models (Wang et al., 2002; Simonyi et al., 2005; Wang et al., 2005; Chuang et al., 2014). Notably, elderberry consumption during ischemia/reperfusion injury reduces the expression of p47Phox (a subunit of NADPH oxidase) and ERK1/2 (a mitogen-activated protein kinase), both of which contribute to neuroinflammation and oxidative stress in microglial cells (Chuang et al., 2014). NADPH oxidase is known to be involved in inflammatory and oxidative processes, including the production of ROS that can lead to apoptosis (cell death) and mitochondrial dysfunction after ischemia (Chen et al., 2011a; Chen et al., 2011b; Yoshioka et al., 2011). Given the link between neuroinflammation, immune activation, and nerve damage in neurodegenerative diseases, elderberry is being explored as a potential therapeutic agent. This study aimed to investigate the potential of elderberry’s antioxidant and anti-inflammatory properties to reduce these processes (Moghaddam et al., 2021). By incorporating elderberry into the daily diet of mice, researchers assessed its effectiveness in a model of neurodegeneration.

### 5.2 Ellagic acid

Ellagic acid (EA), a potent neuroprotective antioxidant, has garnered interest for its potential to mitigate the core pathological hallmarks of HD–a neurodegenerative disorder characterized by a dysregulation of the redox balance and mitochondrial dysfunction. Sharma et al. employed a 3-nitropropionic acid (3-NP) rat model of HD to investigate the efficacy of EA pre-treatment. The study design incorporated six groups: a disease control group receiving 3-NP for 14 days followed by vehicle for 21 days, an EA *per se* group receiving only EA (100 mg/kg) for 21 days, and four EA pre-treatment groups receiving varying doses of EA (25, 50, or 100 mg/kg) 90 min before daily 3-NP administration for 21 days. The findings revealed that oral EA administration (25, 50, and 100 mg/kg) for 3 weeks significantly attenuated neurotoxicity, oxidative and nitrosative stress within the brain, mitochondrial dysfunction, and the behavioral symptomatology of HD in the 3-NP model (Sharma et al., 2021). These results suggest that EA pre-treatment may represent a viable therapeutic strategy for HD.

## 6 Brain aging

Aging is the primary risk factor for most neurodegenerative diseases, causing a cascade of changes throughout the brain (Saha and Sen, 2019). As we age, our brains shrink in size, and blood flow and cognitive function decline (Peters, 2006). These changes manifest at all levels, from alterations in molecules to overall brain structure. Additionally, the incidence of stroke and memory impairment increases with age, along with fluctuations in neurotransmitter and hormone levels. One key contributor to this rapid brain aging is the accumulation of oxidative stress. Antioxidants, therefore, hold promise as therapeutic drugs to slow down age-related cognitive decline (Floyd and Hensley, 2002; Wu et al., 2014; Feng et al., 2016).

### 6.1 Mulberry

The neuroprotective effect of ME was examined *in vivo* and/or *in vitro* models in another unique study by Shin et al. (Shin et al., 2021). Male ICR mice in the *in vivo* model were pretreated with ME (0–200 mg/kg/day, p. o.) throughout the experiment before receiving an intraperitoneal (IP) injection of scopolamine. The *in vitro* model was performed using HT-22 cells. Cells were cultured with ME for 30 min before receiving glutamate treatment. ME improved cell viability, oxidative stress and apoptotic factors in glutamate-treated HT-22 cells, as seen by the results. ME also enhanced the expression of antioxidant enzymes and brain-derived neurotrophic factor (BDNF) by stabilizing activation of the TrkB/Akt pathway and protecting neuronal cells. Additionally, ME decreased the amnesia induced by scopolamine in mice by controlling the activity of cholinergic and antioxidant enzymes. In addition, ME shielded the neuronal cells in the CA1 and CA3 areas of the mouse hippocampus.

### 6.2 Berry active metabolites

Flavonoids are a class of naturally occurring polyphenolic phytochemicals, which are non-nutritional bioactive substances that are widely distributed in plants (Orhan et al., 2015). There is substantial proof that is consuming flavonoids, whether as an extract or as a whole food, can prevent cognitive decline in animal models of standard, non-pathological aging and pathological neurodegenerative diseases (Bhullar and Rupasinghe, 2013; Lamport et al., 2014; Almeida et al., 2016).

Blueberries (*Vaccinium corymbosum* L.), a rich source of flavonoids, are particularly high in anthocyanins, pigments that give many fruits their vibrant colors (Khoo et al., 2017). These anthocyanins, including peonidin, cyanidin, delphinidin, and malvidin (Bhagwat et al., 2014; Khoo et al., 2017), are found not only in blueberries but also in red, blue, and purple berries, red and purple grapes, and red wine (Khoo et al., 2017). According to studies, they can penetrate the blood-brain barrier (Kalt et al., 2008) and anthocyanins may be able to prevent the cognitive deterioration that comes with age (Andres-Lacueva et al., 2005; Spencer, 2010; Rehman et al., 2017; Wei et al., 2017). Beyond neuroprotection, anthocyanins are emerging as potential therapeutic agents for chronic CNS disorders due to their anti-inflammatory and antioxidant properties (Panchal and Brown, 2022). Furthermore, studies indicate that anthocyanins may modulate gut microbiota, offering additional benefits in preventing cardiovascular and neurodegenerative diseases (Winter and Bickford, 2019).

Emerging research suggests that flavonoids may benefit the aging brain through various mechanisms, including reducing inflammation, neutralizing free radicals, and influencing neuroplasticity (Rendeiro et al., 2015). Berries and specific polyphenols, such as flavanols, anthocyanins, and resveratrol, have received significant attention for their potential to promote brain health. These metabolites exhibit several key biological features to enhance neuronal. They can interact with signaling pathways in both neurons and glial cells, protecting neurons from injury and death caused by neurotoxins or neuroinflammation. Additionally, they may modulate the production of ROS and reduce the accumulation of markers associated with neurodegenerative diseases (Bensalem et al., 2015).

A study by Carey et al. employed an *in vitro* model to investigate the effects of blueberry extract and its isolated anthocyanin metabolites on microglial function. BV-2 microglial cells were pre-treated with varying concentrations (0.25, 0.5, 1, or 2 mg/mL) of blueberry extract or individual anthocyanins (pterostilbene, resveratrol, delphinidin-3-O-glucoside, or malvidin-3-O-glucoside) at concentrations of 1, 10, 20, or 30 μM. Following pre-treatment, all groups were exposed to LPS. The study observed that both the blueberry extract and its anthocyanin metabolites, particularly malvidin-3-O-glucoside, significantly reduced the production of TNF-α by BV-2 microglia. Interestingly, the isolated anthocyanins required significantly higher concentrations compared to those found within the whole blueberry extract to achieve a comparable reduction in TNF-α production. For example, 1 mg/mL of blueberry extract, containing approximately 2.6 μM malvidin-3-O-glucoside, effectively reduced LPS-induced nitric oxide release. However, when malvidin-3-O-glucoside was tested in isolation, a much higher concentration of 20 μM was necessary to observe a similar effect. These findings suggest a potential synergistic or additive interaction between various bioactive metabolites within the blueberry extract, contributing to its anti-inflammatory properties (Carey et al., 2013).

### 6.3 Ascorbic acid

Studies explore the potential of bioactive plant metabolites to combat neurodegenerative diseases. A prime example is a study by Nam et al. investigating the effects of ascorbic acid (vitamin C) on D-galactose (D-gal) induced brain aging in mice (Nam et al., 2019). Mice received D-gal (150 mg/kg/day) subcutaneously for 10 weeks, with ascorbic acid (150 mg/kg/day) co-administered orally for the last 4 weeks. Notably, co-administration of ascorbic acid significantly prevented D-gal-induced decline in hippocampal neurogenesis. This was achieved by promoting cellular proliferation, neuronal differentiation, and maturation. Ascorbic acid further protected the hippocampus. It prevented the reduction of synaptophysin and phosphorylated Ca2+/calmodulin-dependent protein kinase II, both markers crucial for synaptic plasticity. Additionally, it countered D-gal’s negative effects on antioxidant enzymes (superoxide dismutase 1 & 2), sirtuin1, caveolin-1, and BDNF (brain-derived neurotrophic factor), while also suppressing inflammatory markers (IL-1β and TNF-α). Ultimately, ascorbic acid’s antioxidant and anti-inflammatory properties led to improved hippocampal recovery and enhanced memory function in D-gal treated mice.

### 6.4 Flavonoids

Flavonoids possess a number of medicinal benefits, including anti-cancer, antioxidant, anti-inflammatory, and anti-viral properties. They also have neuroprotective and cardio-defensive effects. Aurone, a quercetin benzofuranone, another flavonoid that has been shown to inhibit cancer, is a natural flavonoid found in plants. It has generally consumed second-hand foods such as green tea and grains. Luteolin, a botanical drug quercetin, is with botanical druga flavonoid found in plant carcinoma and, in generally, eats up metabolites flavonoid proapoptotic activity in hepatocellular cells, arrests the cancer cell, including berries, green tea and grains. Foods that have ample amounts of flavonoids decrease the chance of neurodegenerative illnesses and decrease the chance of neurodegenerative and additionally counteract age-associated cognitive disorders. Antioxidant flavonoid, abundantly observed in woody plants, has an analogue of 3-O-methyl-epicatechin, which has neuroprotective effects to the polyphenolic luteolin flavonoid, in addition to having a protective effect against inhibition of neurotoxicity *in vitro*. Cyanidin-3-O-glucoside or kuranine is a subgroup of anthocyanins. Kuranine observed in a variety of vegetables and fruits (Ullah et al., 2020).

## 7 Memory loss and impairment

Memory decline is a hallmark symptom of several neurodegenerative diseases, including AD, and can significantly impact daily life and societal wellbeing (Khan et al., 2014). Chronic stress exposure is another factor known to contribute to memory impairments and mood disorders such as anxiety and depression (Ma et al., 2021). Interestingly, research suggests a potential link between the gut microbiome and stress regulation. One study demonstrated that changes in gut microbiota induced by probiotics may lead to reduced stress and anxiety (Ma et al., 2021). Emerging evidence highlights the potential benefits of blueberries for brain health. Blueberry extract exhibits neuroprotective properties and may exert antidepressant-like effects through its interaction with the monoaminergic system and regulation of glucocorticoids, as well as by potentially antagonizing the 5-HT receptor (Ma et al., 2018).

### 7.1 Gallic acid (GA)

Trihydroxybenzoic acid, often known as gallic acid, and its derivatives are natural polyphenol metabolites found primarily in repurposed beverages like red wine and green tea (Graham, 1992). It causes conditioning for antioxidant, anti-inflammatory, antimicrobial, and anticancer responses and safeguards brain cells from amyloid β peptide (Aβ)-induced cell death *in vitro* (Kubo et al., 2001; Bachrach and Wang, 2002; Bastianetto et al., 2006; Borges et al., 2013). Investigating its potential for alleviating chronic stress-induced cognitive decline, Salehi et al. (Bensalem et al., 2015) employed a rodent model. Mice were divided into nine groups: caged control (CC), food-water deprived (FWD), chronic restraint stress (CRS), CRS with GA supplementation (5, 10, and 20 mg/kg), and GA alone (5, 10, and 20 mg/kg). While GA treatment in stressed mice (CRS + GA) improved antioxidant capacity and mitigated brain damage, interestingly, it also induced anxiety-like behavior in healthy mice, *in vivo* and *in vitro*. This suggests that GA’s therapeutic benefits may be limited to individuals already experiencing oxidative stress. Further research is necessary to determine safe and effective dosages for potential clinical applications (Salehi et al., 2018).

A major route of exposure to sodium arsenite (iAS) in living organisms is through contaminated drinking water, with concentrations ranging from 0.01 to 3.7 mg/L. iAS is known to exert neurotoxic effects, including degradation of the myelin sheath, axonal degeneration, vacuolar breakdown, and disruption of synaptic connections (Piao et al., 2011). A study investigated the potential protective effect of gallic acid (GA) against iAS-induced memory impairment. Male rats were divided into six groups (n = 6/group) and received the following treatments for 4 weeks: (i) saline + saline, (ii) saline + GA (50 mg/kg), (iii) saline + GA (100 mg/kg), (iv) iAS + saline, (v) iAS + GA (50 mg/kg), and (vi) iAS + GA (100 mg/kg). The results demonstrated that co-administration of GA with iAS significantly protected against memory dysfunction. Furthermore, the study revealed that subchronic iAS exposure induced anxiety- and depression-like behaviors in control rats as assessed by two behavioral tests for anxiety (elevated plus maze and light-dark avoidance test). Interestingly, rats treated with both GA and saline (GA-saline) or GA and iAS (GA-iAS) displayed reduced anxiety-like behavior compared to their respective controls. Subchronic iAS administration significantly increased anxiety-like behavior, as measured by “pang exertion”. This finding suggests that GA may improve memory function by mitigating anxiety-related behaviors (Samad et al., 2019).

## 8 Hippocampal damage and memory loss

Research suggests that stress may damage the hippocampus through multiple mechanisms. One way is by regulating the production of neurotrophic factors, chemical messengers that support nerve cell growth and survival. A recent study found that stress alters the expression of these factors in the brain. Additionally, chronic exposure to stress hormones (glucocorticoids) can cause shrinkage of dendritic processes within the hippocampus in as little as 21 days. Notably, stress is associated with a decrease in BDNF messenger RNA (mRNA), which may contribute to stress-induced damage. However, the effects of stress on other neurotrophic factors, such as nerve growth factor and neurotrophin-3, are less clear. While some studies suggest an increase in these factors with stress, their role in protecting the hippocampus from damage remains unclear (Smith, 1996).

### 8.1 Ellagic acid (EA)

Ellagic acid (EA), a metabolite of berries, offers a multitude of benefits in the brain. It significantly reduces damaging molecules like reactive ROS and MDA in the hippocampus, a region crucial for memory. Conversely, EA significantly boosts the levels of beneficial antioxidants like GSH and manganese superoxide dismutase (MnSOD) in the same area. Further supporting its neuroprotective role, EA’s hypoglycemic effects (lowering blood sugar) appear to combat oxidative stress in diabetic rats. This effect is likely due to its ability to neutralize ROS and activate Nrf2, a protein that coordinates the body’s antioxidant defenses. As Alfaris et al. demonstrated, EA may prevent hippocampal damage and memory loss caused by diabetes. In their study, Adult male rats were divided into 4 groups (n = 12) as control, control + EA (50 mg/kg), STZ-DM, and STZ-DM + EA. Treatments were given orally and daily for 8 weeks. EA treatment preserved the structure of the hippocampus, particularly the CA1 region, and countered memory decline. Additionally, EA increased body weight gain, insulin levels, and beneficial proteins like BDNF and Bcl-2, while decreasing inflammatory markers (TNF-α and IL-6) and plasma glucose levels. Notably, EA’s effects were consistent across both control and diabetic rats. It significantly lowered ROS and MDA, boosted GSH and MnSOD, and activated Nrf2 in the hippocampus of both groups. Moreover, EA increased the activity of multiple signaling pathways crucial for neuronal health and survival. These findings suggest that EA exerts its neuroprotective effects through a complex interplay of processes, involving the activation of beneficial pathways and the suppression of detrimental factors (Alfaris et al., 2021).

### 8.2 Gallic acid

A variety of phenolic acids such as GA, protocatechuic acid, p-hydroxybenzoic acid, vanillic acid, caffeic acid, p-coumaric acid, ferulic acid, EA and cinnamic acid were present in the blueberry, blackberry and strawberry samples tested (flavone luteolin; flavonols rutin, myricetin, quercitrin and quercetin; flavanols gallocatechin, epigallocatechin, catechin and catechin gallate; anthocyanidins malvidin-3-galactoside, malvidin-3-glucoside and cyanidin). The large amounts of pro-anthocyanidins and anthocyanidins, particularly in blueberries, may be at the root of their powerful antioxidant conditioning (Huang et al., 2012). GA influences oxidative labels (such as lipid peroxidation and catalase activity) in the liver and hippocampus of subjects subchronically exposed to ketamine. After a seizure, treatment with GA protected for the brain’s feathers and hippocampus, but did not restore working memory, which had been damaged by blood loss (Brum et al., 2020).

## 9 Stress-induced memory deficits

Chronic stress can negatively impact the central nervous system by promoting oxidative stress, a process that damages cells. This damage, including neuron loss, is particularly detrimental to the hippocampus, a brain region crucial for memory (McEwen and Sapolsky, 1995). Studies have revealed a connection between elevated stress levels and accelerated cognitive decline in older adults (over 65) (Aggarwal et al., 2014). Furthermore, research suggests that women experiencing high levels of chronic stress may have a 60% increased risk of developing dementia (Johansson et al., 2010).

### 9.1 Ascorbic acid

**FIGURE 1:**
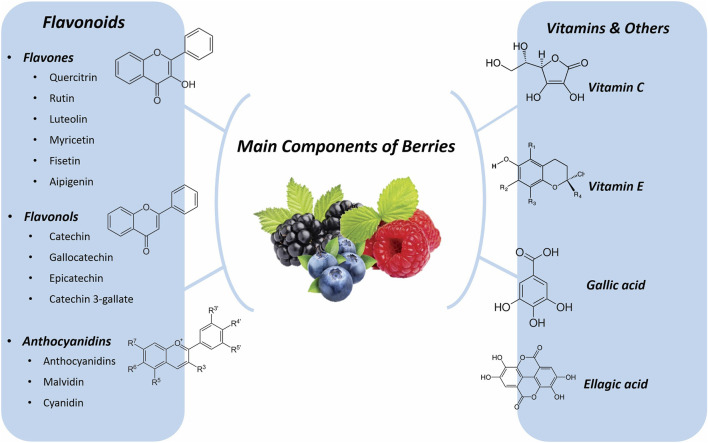
Main component of Berries.

Ascorbic acid, also known as vitamin C, is primarily produced in plants through the L-galactose pathway. Five genes play crucial roles in this pathway: guanosine diphosphate-mannose-3′,5′-epimerase (GME), guanosine diphosphate-L-galactose phosphorylase (GGP), L-galactono-1,4-lactone dehydrogenase (GLDH), and monodehydroascorbate reductase (MDHAR) (Ma et al., 2018). In a study, Kumar et al. explored the role of ascorbic acid and its protective impacts on memory loss caused by persistent restraint stress. The adult male Wistar rats were divided into the following groups: (I) Normal control, (II) Ascorbic acid treatment, (III) Vehicle control, (IV) Restraint stress (who were stressed in line mesh restrainers for 6 hours per day for 21 days), (V) Restraint-stress vehicle (These rats were stressed 6 hours per day for 21 days while entering an equal volume of a vehicle) and (VI) Restraint-Ascorbic acid treatment vehicle. These rats received oral ascorbic acid therapy daily at 100 mg/kg/body weight, while under stress 6 h a day for 21 days. After 21 days, the animals were tested for their memory using the Morris water maze and passive avoidance apparatus. The final results of the experimental groups also differed significantly. Compared to animals in other intervention groups, rats that underwent only restraint stress and those pretreated with vehicle results before restraint stress showed impairments in literacy and decreased memory retention in the memory tests. The same memory tests revealed a considerable improvement in memory retention in creatures that had undergone ascorbic acid pretreatment before being constrained. Restraint stress has been linked to oxidative brain damage and the emergence of free revolutionaries. When given orally under obsessive stress, ascorbic acid caused a substantial enhancement in the amount of dehydroascorbic acid entering the rat brain, which serves as a protective barrier against unrestrained revolutionaries. Ascorbic acid can therefore be used to successfully treat neurological problems associated with stress that have an impact on cognition (Kumar et al., 2009). (Figure 1 shows the main metabolite of berries.)

## 10 Neurodegenerative diseases

In the United States alone, over 6 million people battle neurodegenerative diseases like Alzheimer’s (AD), Parkinson’s (PD), Huntington’s, and Amyotrophic Lateral Sclerosis (ALS). Currently, no medications can halt or reverse these conditions. Age is the biggest risk factor, with age-related changes in the brain making it more susceptible (Maher, 2019b). These changes include increased oxidative stress, altered energy metabolism, reduced neurotrophic support (essential for neuron survival), and protein processing problems that lead to protein clumps. Additionally, the neurovascular system (supplying blood to the brain) weakens, the immune system overreacts, synapses (connections between neurons) malfunction, and inflammation rises. This combination triggers behavioral and cognitive decline (Bishop et al., 2010; Prior et al., 2014).

### 10.1 Flavonoids

While traditionally seen for their antioxidant properties (Jones et al., 2012; Duarte et al., 2017; de Andrade Teles et al., 2018; Kujawska and Jodynis-Liebert, 2018), recent research suggests factory-made flavonoids might be even more effective due to their broader impact on pathways linked to neurodegeneration. Fruits like apples (*Malus domestica Borkh.*, Rosaceae) and berries (various species from Rosaceae, Ericaceae, and Vitaceae families), red wine (made from grapes, *V. vinifera* L., Vitaceae), citrus fruits (various species from Rutaceae family), and tea (leaves from *Camellia sinensis* (L.) *Kuntze*, Theaceae) are all rich sources of these natural flavonoids. Preclinical studies are promising, encouraging further investigation into their potential for treating neurodegenerative diseases (Maher, 2019b). There are over 5,000 different flavonoids, categorized by their chemical structure. Interestingly, studies suggest that consuming flavonols, a specific type of flavonoid, may be linked to a reduced risk of dementia (Commenges et al., 2000; Beking and Vieira, 2010). One study found a nearly 50% lower risk of dementia in people with the highest flavonol intake compared to those with the lowest. Another study showed a negative association between total flavonoid intake and dementia risk. However, the link between flavonoids and PD seems less clear. While one study found a reduced risk of PD in men with high flavonoid intake, the effect was not observed in women (Gao et al., 2012). More research is needed to understand these sex-based differences.

## 11 Cerebral hypoxia and ischemia

Ischemic stroke, a major global health issue, claims millions of lives annually (over 6.6 million) and leaves many more disabled (Lo et al., 2003; Glantz et al., 2006; Sun et al., 2015; Writing Group et al., 2016). It occurs when a blood clot blocks blood flow to the brain, causing immediate damage (Silvestrelli et al., 2002; Rosamond et al., 2008). While age is the only unpreventable risk factor, other health conditions like high blood pressure and diabetes worsen its effects (Popa-Wagner et al., 2020). As the population ages, stroke cases and survivors with disabilities are rising, placing a significant social and economic burden on society (Popa-Wagner et al., 2020). This increase in stroke prevalence is largely attributed to growing risk factors like obesity, atrial fibrillation, and high cholesterol, which are more common in older adults (Roy-O’Reilly and McCullough, 2018).

### 11.1 Flavonoids

Flavonoids, a class of bioactive metabolites naturally occurring in berries, possess well-documented anti-aging, neuroprotective, antioxidative, and anti-neuroinflammatory properties (Cheng et al., 2022). Rivera et al. investigated the potential neuroprotective effects of quercetin (*Quercus* spp. L.), fisetin (*Rhus coriaria* L.), and catechin (*C. sinensis* (L.) *Kuntze*) against ischemic stroke damage using a rat model. To enhance brain bioavailability, these flavonoids were encapsulated in liposomal formulations for intraperitoneal (IP) administration. Localized ischemic injury was induced via middle cerebral artery occlusion (MCAO), and infarct volume was quantified spectrophotometrically using the 2,3,5-triphenyltetrazolium chloride (TTC) staining method. Additionally, hematoxylin-eosin (H&E) staining was employed to evaluate the integrity of brain tissue architecture. While no detectable levels of administered flavonoids were observed in the brain tissue, significant neuroprotective effects were evident with liposomal quercetin. Spectrophotometric analysis of TTC-stained brain sections revealed a reduction in infarct volume following permanent MCAO in rats treated with liposomal quercetin. Furthermore, H&E staining demonstrated improved cytoarchitecture preservation within the ischemic striatal and cortical regions of these animals. Similarly, fisetin administered in a liposomal formulation exhibited neuroprotective effects, although to a lesser extent compared to quercetin. Interestingly, catechin treatment did not offer any significant protection against ischemic damage. These findings suggest that early administration of liposomal quercetin and potentially other structurally similar flavonoids may provide a neuroprotective strategy in the context of experimental focal ischemia. Further studies are warranted to validate these observations and elucidate the underlying mechanisms of action for this promising therapeutic approach (Rivera et al., 2004).

Luo et al. investigated the neuroprotective potential of total flavonoids isolated from *Hibiscus esculentus* L. flowers against transient cerebral ischemia-reperfusion (I/R) injury in a mouse model. Mice receiving a daily oral administration of 300 mg/kg *Abelmoschus esculentus* (L.) *Moench* flower extract for 1 week exhibited significantly reduced infarct volume, neurological deficits, and histological alterations in brain tissue compared to the control group. Mechanistically, this treatment regimen was associated with increased levels of SOD, an antioxidant enzyme, and decreased levels of NO and MDA, markers of oxidative stress. The study employed a randomized design with five groups: a model group and treatment groups receiving *A. esculentus* flower extract at high (300 mg/kg), medium (150 mg/kg), and low (75 mg/kg) doses. The safety of the 300 mg/kg dose was established. These findings suggest that *A. esculentus* flower extract may protect against I/R injury by scavenging free radicals and activating the Nrf2/antioxidant response element (ARE) pathway, a key regulator of the cellular antioxidant response (Luo et al., 2018).

Flavonoids are believed to exert their neuroprotective effects through direct antioxidant activity within the brain parenchyma. Experimental studies have shown that flavonoid intake can mitigate brain damage caused by oxidative stress, a condition characterized by an imbalance between ROS and antioxidant defenses. To investigate the effects of chronic dietary flavonoid intake on ischemic stroke, researchers have employed models that mimic realistic dietary patterns. In one such study, mice were fed a soy-containing diet for 5 weeks before undergoing transient middle cerebral artery occlusion (MCAO) for 90 min. Chronic soy consumption led to a significant reduction in infarct volume and neurological deficits compared to the control group (Schreihofer et al., 2005; Burguete et al., 2006). Another study examined the neuroprotective potential of naringenin, a specific flavonoid found in citrus fruits. Rats subjected to bilateral carotid artery occlusion for 5 minutes displayed improved neurological and functional outcomes following 10 days of naringenin administration (50 or 100 mg/kg) starting 1 week before the ischemic challenge (Aggarwal et al., 2010).

### 11.2 Anthocyanin

A variety of phenolic acids (including GA, p-hydroxybenzoic acid, protocatechuic acid, caffeic acid, vanillic acid, p-coumaric acid, EA, ferulic acid, and cinnamic acid) and various flavonoids were present in the blueberry, strawberry, and blackberry samples tested (flavone: luteolin; flavonols: myricetin, quercitrin, rutin, and quercetin; flavanols: epigallocatechin, gallocatechin, catechin, and catechin gallate; anthocyanidins: cyanidin, malvidin-3-glucoside, and malvidin-3-galactoside). Anthocyanidins and proanthocyanidin levels were exceptionally high in blueberries, which may be the reason for their potent antioxidant properties (Huang et al., 2012).

Anthocyanins, a class of natural antioxidants, have demonstrated therapeutic potential in various inflammatory, cardiovascular, and neurological diseases (Zafra-Stone et al., 2007). Shin et al. investigated their neuroprotective effects in a rat model of ischemic stroke induced by middle cerebral artery occlusion (MCAO) [119]. Oral administration of anthocyanins (300 mg/kg) at 24 h and 30 min before MCAO significantly reduced brain damage and ameliorated neuronal injury. Mechanistically, anthocyanins were found to promote autophagic flux, a cellular recycling process, in SH-SY5Y cells exposed to simulated ischemic conditions (glucose and oxygen deprivation). This action likely contributed to the observed reduction of oxidative stress, inflammatory response, and ultimately, neuronal death. Additionally, anthocyanin treatment significantly decreased the number of p-JNK (phosphorylated c-Jun N-terminal kinase) and p53-positive cells in the infarct area, suggesting its ability to suppress JNK activation and p53 upregulation, both of which are detrimental signaling pathways in ischemic injury. These findings, along with the observed reduction in neurovascular damage, support the potential of anthocyanins as a neuroprotective agent in ischemic stroke (Shin et al., 2006).

Supporting the potential neuroprotective role of anthocyanins, Cai et al. demonstrated a link between cerebral ischemia and oxidative stress, suggesting that reducing oxidative stress may be a strategy for stroke prevention. Their *in vitro* study using SH-SY5Y cells exposed to simulated ischemia (oxygen and glucose deprivation) revealed a dose-dependent effect of anthocyanin (50–200 μg/mL) on inflammatory factors and ROS. Ischemia significantly increased the activation of NF-κB and the levels of ROS, NF-κB, TNF-α, IL-6, and IL-1β in these cells. Notably, anthocyanin treatment significantly inhibited these ischemia-induced increases. Additionally, anthocyanin suppressed the expression of hypoxia-inducible factor 1 (HIF-1), a key regulator of the cellular response to low oxygen conditions. These findings suggest that anthocyanins may exert their neuroprotective effects by mitigating oxidative stress and inflammatory responses triggered by ischemic conditions (Cai et al., 2020).

Min et al. investigated the neuroprotective effects of cyanidin-3-O-glucoside (CG), an anthocyanin, in a mouse model of localized cerebral ischemia induced by permanent middle cerebral artery occlusion (pMCAO). Mice were randomized into vehicle (n = 10) and CG treatment groups (1 mg/kg, n = 8; 2 mg/kg, n = 10; 5 mg/kg, n = 9) receiving oral administration. Neurological function was assessed using a scoring system 24 h after pMCAO. Pre-treatment with 2 mg/kg CG significantly reduced infarct volume by 27% compared to the vehicle group. Notably, a similar reduction (25%) in infarct size was observed even with delayed administration (post-treatment) of 2 mg/kg CG. Both pre- and post-treatment with CG resulted in significantly improved neurological function. These findings suggest that CG protects mice from pMCAO-induced ischemic damage. Mechanistically, the authors propose that CG’s neuroprotective effects may be attributed to its ability to decrease brain superoxide levels following ischemia and prevent the translocation of apoptosis-inducing factor (AIF) into the mitochondria, a pathway leading to cell death (Min et al., 2011).

### 11.3 Ascorbic acid

Berries are rich sources of bioactive metabolites, including ascorbic acid, phenolic acids, tannins, and various flavonoids like anthocyanins and flavonols (Skrovankova et al., 2015a). This review explores the antioxidant capacity and health benefits of these bioactive metabolites in commonly consumed berries. Ascorbic acid, in particular, has garnered interest for its potential neuroprotective role in acute cerebral ischemia, a major cause of stroke-related disability and death (Al-Majed et al., 2006; Rossi et al., 2007). Cerebral ischemia, typically induced in animal models or following cardiorespiratory arrest such as myocardial infarction, triggers widespread neuronal death leading to disability and dementia (Horn and Schlote, 1992; Böttiger et al., 1998; Zhu et al., 1998; Kudo et al., 2006; Ozacmak et al., 2007). During the initial stages of ischemia, the brain experiences a critical shortage of ascorbic acid alongside insufficient glucose and oxygen clearance (Dirnagl et al., 1999; Rice, 2000). This creates a perfect storm for oxidative stress, as excessive glutamate receptor stimulation leads to free radical and ROS production, ultimately causing brain damage (Margaill et al., 2005; Moro et al., 2005). Ascorbic acid, a potent antioxidant, is believed to counteract these ROS and exert neuroprotective effects (Rice, 2000). Studies have demonstrated a significant rise in ascorbic acid levels within the cortex following 2-vessel occlusion-induced global cerebral ischemia. Interestingly, the dorsal and ventral hippocampus also showed increased ascorbic acid levels, albeit to a lesser extent. These findings highlight the regional heterogeneity in extracellular ascorbic acid response to ischemia, despite a uniform reduction in cerebral blood flow (Liu et al., 2009).

### 11.4 Ellagic acid

Wang et al. investigated the neuroprotective potential of a bioactive metabolite, ellagic acid (EA), isolated from a traditional Tibetan medicine formulation (“Sibuyidian, Shanhu, and Ruyi Zhenbao”) in a rat model of transient cerebral ischemia-reperfusion (I/R) injury [135]. Following *in vivo* and *in vitro* analysis of the medicinal metabolites, the therapeutic effects of EA against I/R were evaluated by measuring infarct volume in pMCAO models. Rats were randomly allocated to nine groups: sham, I/R, nimodipine (positive control), and treatment groups receiving “Shanhu” pills, “Ruyi Zhenbao” pills, “Chenxiang” pills, or EA at low (10 mg/kg), medium (30 mg/kg), or high (50 mg/kg) doses. All drugs were administered orally in saline solution. The study identified EA as a promising candidate for ischemic stroke treatment. The authors propose that EA exerts its neuroprotective effects through antioxidant and anti-inflammatory mechanisms. Additionally, EA treatment was associated with reduced blood-brain barrier (BBB) permeability, indicated by decreased zonula occludens-1 (ZO-1) and matrix metalloproteinase-9 (MMP-9) expression and aquaporin-4 (AQP4) upregulation, potentially limiting further brain damage. Notably, EA did not activate coagulation factors, suggesting a safe therapeutic profile (Wang et al., 2019).

Ischemic brain disease affects approximately 2 million people annually, with a concerning rise in young individuals. Zhang et al. investigated the neuroprotective effects of Crataegus flavonoids (CF), a dietary antioxidant, against cerebral ischemia-reperfusion (I/R) injury in a Mongolian gerbil model. Animals were randomly assigned to four groups: I/R (ischemia for 5 min after 15 days of drinking water), sham (same treatment as I/R but without arterial occlusion), low-dose CF (0.5 mg/mL CF in drinking water), and high-dose CF (2.5 mg/mL CF in drinking water). Pretreatment with CF significantly reduced ROS production, thiobarbituric acid reactive substances (TBARS) content, and nitrite/nitrate levels in brain homogenates, while increasing total antioxidant capacity in a dose-dependent manner. Mechanistically, CF pretreatment was proposed to enhance bioavailable NO levels by scavenging superoxide anions, which can react with NO to form the detrimental peroxynitrite. Notably, while I/R injury led to increased nitrite/nitrate (NO degradation product) and decreased detectable NO (measured by electron spin resonance), CF pretreatment reversed these trends, suggesting improved NO bioavailability. This effect might be attributed to CF’s superoxide anion scavenging ability, preventing its reaction with NO. Additionally, CF pretreatment decreased protein levels of TNF-α and NF-κB, both pro-inflammatory mediators implicated in delayed neuronal death after ischemia. Conversely, CF treatment increased the mRNA level of endothelial nitric oxide synthase (eNOS), potentially promoting vasodilation and neuroprotection (Zhang et al., 2004). These findings suggest that Crataegus flavonoids can alleviate cerebral oxidative stress and inflammation following I/R injury, potentially by interacting with the blood-brain barrier.

### 11.5 Pyruvate, lactate, and ascorbic acid

Pyruvate, a key intermediate in cellular metabolism, has been shown to enhance cellular resilience against hypoxia through its anti-inflammatory and antioxidant properties (Ryou et al., 2012). Conversely, lactate, produced from pyruvate under anaerobic conditions, can be converted back to pyruvate by the brain and utilized as an energy substrate via mitochondrial oxidation (Berthet et al., 2009). Interestingly, direct administration of lactate to the brain following reperfusion has demonstrated neuroprotective effects against ischemia-induced cell death and disability.

Cheng et al. employed a novel *in vivo* approach utilizing microdialysis integrated with liquid chromatography to continuously monitor brain chemistry during cerebral ischemia in gerbils. This technique allowed for real-time monitoring and high spatiotemporal resolution of key metabolites throughout the ischemic and reperfusion phases. Focal cerebral ischemia was induced by occluding a common carotid artery for 60 min followed by 3 h of reperfusion. Microdialysis probes were inserted into the gerbils’ striatum to measure brain levels of lactate, pyruvate, and ascorbic acid. Notably, the study observed significant biphasic increases in both lactate and ascorbic acid levels during I/R injury. Standard L-lactate stock solutions (100 and 10 mM) were prepared in 4 mM sulfuric acid and stored at 4°C, while fresh ascorbic acid solutions (100 mM) were prepared daily (Cheng et al., 2000).

### 11.6 Gallic acid

Sun et al. investigated the neuroprotective effects of gallic acid (GA) against cerebral I/Rinjury using both *in vitro* and *in vivo* models. *In vitro*, human SH-SY5Y neuroblastoma cells were subjected to hypoxia/reoxygenation following pre-treatment with varying concentrations of GA (0.1, 1, and 10 μM) for 24 h. *In vivo*, male Sprague-Dawley rats received GA (25 or 50 mg/kg) intravenously 20 min before middle cerebral artery occlusion (MCAO) to induce ischemia. The study demonstrated that GA pretreatment mitigated oxidative stress by regulating mitochondrial ROS production. This, in turn, resulted in partial reversal of hypoxia/reoxygenation-induced damage and prevented mitochondrial dysfunction by inhibiting mitochondrial permeability transition pore (MPTP) opening (Sun et al., 2014).

## 12 Cerebral vasospasm

Cerebral vasospasm is an important part of the extreme changes in aneurysmal subarachnoid hemorrhage. The location and quantity of subarachnoid clots are immediately associated with the incidence, distribution, and severity of the vasospasm. This vasospasm may be triggered by spasmogenic materials launched from the clot (Nassar et al., 2019).

### 12.1 Urokinase and ascorbic acid

Kodama et al. investigated a novel therapeutic approach to prevent symptomatic vasospasm, a complication arising after surgery for acute subarachnoid hemorrhage (SAH). This method involves the intra-arterial infusion of a combination agent containing urokinase and ascorbic acid. Urokinase, a thrombolytic enzyme, aims to dissolve residual subarachnoid clots, while ascorbic acid, an antioxidant, scavenges oxyhemoglobin, a potent vasoconstrictor (Kodama et al., 2000; Kodama et al., 2001). The study evaluated the efficacy of this combination therapy in a cohort of 217 patients who underwent surgical intervention within 72 h of SAH onset. All patients received a continuous intra-arterial infusion of Lactated Ringer’s solution containing urokinase (120 IU/mL) and ascorbic acid (4 mg/mL) at a rate of 30 mL/hour per side for approximately 10 days. The study demonstrated promising outcomes for the combination therapy. Symptomatic vasospasm, a complication of concern, only occurred in a small subset of patients (6 out of 217, translating to 2.8%). Even among these cases, only two patients (0.9%) experienced lasting sequelae. The average amount of blood removed during the irrigation process was approximately 114 mL. Analysis of the drainage fluid confirmed the effectiveness of the treatment in eliminating oxyhemoglobin, a key contributor to vasospasm, as evidenced by the disappearance of the specific absorption peak at 576 nm. It is important to note that eight patients experienced complications during the irrigation therapy, including seizures (2 patients), meningitis (2 patients), and intracranial hemorrhage (4 patients). However, all patients recovered fully without any lasting neurological deficits (Kodama et al., 2000).

### 12.2 Ascorbic acid and Mg

Despite surgical intervention for ruptured aneurysms, the incidence of postoperative symptomatic vasospasm remains a significant concern, ranging from 15% to 30% according to recent reports (Suzuki et al., 2007; Yoneda et al., 2008; Liu et al., 2016). This complication can worsen neurological outcomes, highlighting the need for effective preventive measures. Promising strategies focus on eliminating or reducing oxyhemoglobin within subarachnoid clots and supplementing with magnesium (Mg2+) (Sonobe and Suzuki, 1978; Macdonald and Weir, 1991; Humphrey et al., 2007; Wong et al., 2008; Sehba et al., 2011). Satoh et al. investigated the efficacy of continuous cisternal irrigation with a mock CSF solution containing ascorbic acid and magnesium following surgery for a ruptured aneurysm. This mock CSF was prepared by adding ascorbic acid (4 mg/mL) and magnesium sulfate (3.0 mEq/L) to a lactate ringer’s solution, with the pH adjusted to 7.4 using sodium bicarbonate. Ten to 12 days post-surgery, cisternal irrigation was performed by infusing the mock CSF into the lateral ventricle at a rate of 20 mL/hour. The study demonstrated the effectiveness of this approach in preventing symptomatic vasospasm and even in mitigating its severity when it did occur (Satoh et al., 2010).

## 13 Cerebrovascular disease

The term “cerebrovascular disease” describes the disorder that affects blood flow in the brain due to vascular problems such as stenosis (blood vessel narrowing), thrombosis (clotting), embolism (artery blockage), and hemorrhage (blood vessel rupture) (Welch et al., 1997). The worldwide cost of CVA is approximately $721 billion (0.66% of the world economy). It is necessary to point out that the occurrence of cerebrovascular conditions rises along with age. Stroke is a primary cerebrovascular disease that impacts the patient’s wellbeing (Hollander et al., 2003).

### 13.1 Flavonoids

Research suggests that dietary flavonoids offer a range of health benefits, including neuroprotection and improved cardiovascular health (Mink et al., 2007; Mursu et al., 2008; Vauzour et al., 2008; Cassidy et al., 2011; McCullough et al., 2012; Peterson et al., 2012; Arranz et al., 2013; Wang et al., 2014; Bondonno et al., 2015). Epidemiological studies support this connection, demonstrating a positive association between a flavonoid-rich diet and heart health (Schroeter et al., 2006).

One key mechanism by which flavonoids may exert their cardiovascular benefits is through their antihypertensive effect (Grassi et al., 2015; Sansone et al., 2015). This may be mediated by an improvement in endothelial function, the proper functioning of the inner lining of blood vessels (Fraga et al., 2010). Additionally, flavonoids may directly influence blood pressure by inhibiting the activity of ACE, a key regulator (Guerrero et al., 2012).

Flavonoid consumption has also been shown to benefit cognitive function, potentially mitigating age-related cognitive decline (Williams and Spencer, 2012). This neuroprotective effect is believed to involve mechanisms such as increased bioavailability of NO and optimal blood flow to the brain (de la Torre and Aliev, 2005; Aliev et al., 2009).

Enhanced NO levels promote blood flow to the brain, supporting the growth of nerve cells and potentially influencing the dentate gyrus of the hippocampus, a key region for memory function (Pereira et al., 2007; Spencer, 2010). Furthermore, flavonoids may be able to enter the brain and exert their protective effects by reducing neuroinflammation and enhancing the plasticity of connections and synapses (Abd El Mohsen et al., 2002; Youdim et al., 2004; Sokolov et al., 2013; Rodriguez-Mateos et al., 2014; Chen et al., 2015). These combined effects may contribute to slowing the progression of cognitive decline.

So far, many studies have shown the beneficial effects of flavonoid foods on cerebral blood flow (CBF). Whose mechanism is based on the increasing peripheral blood flow (Francis et al., 2006; Lamport et al., 2012). Blueberries were found to increase regional perfusion, with an increase in CBF observed in the precentral and middle frontal gyrus of the frontal lobe and the angular gyrus of the parietal lobe following consumption of a flavonoid-rich (579 mg) blueberry beverage compared to a control (Dodd, 2012). Further, a more recent study in healthy adults also found an increase in regional perfusion, specifically in the parietal and occipital lobes, following 12 weeks of supplementation with a blueberry concentrate containing 387 mg of anthocyanins (Bowtell et al., 2017). In addition, regional perfusion in the frontal and melanic gyrus has been observed after consumption of citrus fruits (Rutaceae family) (Lamport et al., 2016).

## 14 Chronic cerebral hypoperfusion

Part of the aging process is brain hypoperfusion. However, in areas such as the hippocampus and anterior cingulate cortex, cardiovascular risk factors cause a more significant decrease in CBF (Grolimund and Seiler, 1988; Dai et al., 2008; de La Torre, 2012; Di Marco et al., 2015). Chronic cerebral hypoperfusion is a significant risk factor for vascular dementia and can also play an etiological role in AD. How chronic cerebral hypoperfusion leads to cognitive impairment and its contribution to Alzheimer’s pathology is not well understood (Zhao et al., 2014).

### 14.1 Mulberry fruit and ginger (PMG)

This study investigated the potential neuroprotective effects of a phytosomal complex containing white mulberry leaves (*Morus alba* L., Moraceae) and extract of ginger (*Zingiber officinale Roscoe,* Zingiberaceae) (PMG) against ischemic stroke in a metabolic syndrome model (Palachai et al., 2020). The findings revealed that PMG administration significantly improved neurological deficits and brain damage in a rat model of cerebral ischemia-reperfusion injury induced by high-carbohydrate, high-fat diet feeding. The authors propose that the observed benefits may be attributed to the combined effects of mulberry leaf extract and ginger extract, both known to possess antioxidant and anti-inflammatory properties. These properties are believed to enhance the body’s antioxidant defense system, thereby mitigating oxidative stress and reducing brain damage. Based on the reported antioxidant and anti-inflammatory properties of PMG, as well as the observed neuroprotective effects in this study, the authors suggest that the combined extracts of mulberry leaf and ginger in PMG may be responsible for the neuroprotective benefits.

### 14.2 Ellagic acid

Pang et al. designed a dose-response experiment. Rats were randomly assigned to a sham group or one of three EA-treated groups receiving varying doses (0.5, 1.0, or 1.5 mg/mL; n = 8–10). Intracerebral infusion of EA resulted in localized central cerebral necrosis in a dose-dependent manner. This correlated with a significant increase in neurological deficit scores and the brain weight to body weight ratio. Additionally, EA dose-dependently elevated serum lactate dehydrogenase activity and malondialdehyde levels, while decreasing serum superoxide dismutase activity. These findings provide the first evidence that EA can induce central cerebral ischemia in rats, potentially mimicking the pathophysiology of human ischemic stroke. This opens doors for further investigation of EA’s potential in preclinical, pharmacological, and clinical settings for ischemic stroke (Pang et al., 2014).

### 14.3 Ascorbic acid

In the study by Varshosaz et al. the physico-chemical characteristics of the formulas were evaluated under laboratory conditions to investigate the injection of ascorbic acid and α-tocopherol for the prevention of CVA. They used vesicular formulations containing ascorbic acid or α-tocopherol. Niosomes of ascorbic acid were prepared by dissolving 400 μmol surfactants (equal mole percent Tween/Span with the same hydrocarbon chain type and length)/chol in chloroform in a round bottom flask. Rats were ischemic for 30 min using the middle cerebral artery occlusion model, and the selected formulation was used to test its neuroprotective effect against cerebral ischemia *in vivo*. This study showed that niosomes had a more potent neuroprotective effect against cerebral ischemic injury in male rats than free ascorbic acid. In this study, two combinations of ascorbic acid and α-tocopherol were devised in niosomes composed of sorbitan esters and their ethoxylated derivatives. Cholesterol content and the hydrophilicity potential of condensate combinations were the main factors affecting the mean volume diameter of the prepared vesicles. It was deduced that the efficacy of the invented new drug delivery system in protecting brain tissue contra upgrade in the concentration of oxygen free radicals during the cerebral ischemia-reperfusion route was more than of free ascorbic acid. Furthermore, the niosomal formulation provides an appropriate possible way for i V-management of a water-insoluble drug like α-tocopherol (Varshosaz et al., 2014).

## 15 Epilepsy

Epilepsy is a chronic neurological disorder characterized by recurrent seizures. These seizures arise from abnormal electrical activity within the brain, often caused by an imbalance between excitatory and inhibitory neurotransmission. Glutamate, the major excitatory neurotransmitter, is thought to be overactive, while gamma-aminobutyric acid (GABA), the main inhibitory neurotransmitter, is believed to be insufficient (de Almeida et al., 2008; Grosso et al., 2013). Epilepsy affects 1%–2% of the global population, making it the most common neurological condition worldwide. The incidence varies geographically, with developed countries reporting around 50 cases per 100,000 people compared to 100 cases per 100,000 in developing countries (Ramalingam et al., 2013). It is a condition that transcends age, race, and socioeconomic background (Gupta et al., 2014). Notably, research suggests a link between epilepsy and oxidative stress, a cellular imbalance characterized by an excess of free radicals and a weakened antioxidant defense system. This oxidative stress may contribute to both the development and progression of epilepsy, mirroring its role in human aging (Dato et al., 2013).

### 15.1 Flavonoids

Several studies suggest that flavonoids possess antiepileptic properties. Their structural similarity to benzodiazepines allows them to modulate the GABAA-Cl-channel complex, potentially mimicking the effects of these medications (Choudhary et al., 2011). Additionally, the phenolic nature of flavonoids enables them to act as antioxidants, potentially interrupting harmful cellular oxidative processes within the central nervous system, which may be relevant in neurodegenerative diseases (Costa Marques et al., 2013). Research has explored the anticonvulsant effects of specific flavonoids, including rutin, quercetin, and isoquercitrin, in experimental epilepsy models (Orhan et al., 2012). For instance, Gupta et al. demonstrated that morusin (*M. alba* L.) treatment significantly reduced the severity of seizures induced by maximal electroshock (MES) (Gupta et al., 2014). Another study investigated the anticonvulsant activity of an ethanol extract from *Abelmoschus manihot* L. (Malvaceae) using the pentylenetetrazol (PTZ)-induced seizure model. The extract displayed anticonvulsant effects following oral administration, and further analysis revealed isoquercitrin, hyperoside, hibifolin, quercetin-3-O-glucoside, quercetin, and isorhamnetin as potential active metabolites within the extract (Guo et al., 2011).

Studies have investigated the anticonvulsant properties of several flavonoids. Apigenin, for example, reduced the latency of seizure onset and locomotor activity in rats with picrotoxin-induced convulsions (Jager et al., 2009). Vitexin, a C-glycosylated flavone, extended the time to seizure onset in models of pentylenetetrazole-induced seizures, possibly by interacting with the benzodiazepine site of the GABAA receptor complex (Abbasi et al., 2012). Hispidulin demonstrated anticonvulsant activity by decreasing glutamate release through mechanisms involving both voltage-gated calcium channels and the exocytotic machinery (Lin et al., 2012). Finally, linarin and its aglycone acacetin have also been shown to possess sedative and anticonvulsant effects in animal studies (Nugroho et al., 2013).

## 16 Atherosclerosis

Atherosclerosis is a chronic inflammatory disease of the large arteries expressed by plaque aggregation in the arterial wall. It is deliberated as the significant risk factor for the expansion of cardiovascular diseases (Libby et al., 2011). It is further associated with premature biological aging, as atherosclerotic plaque appearance evidence of cellular old age characterized by abated cell proliferation, irrevocable growth arrest, apoptosis, epigenetic modifications, elevated DNA damage, and telomere shortening (Wang and Bennett, 2012).

### 16.1 Flavonoids

Researchers investigated how a flavonoid-rich extract from *Polygonum capitatum* (FPC) affects blood vessel inflammation, oxidative stress, and blood fat levels in rats fed a high-fat diet (Wang and Jiang, 2018). The rats were given two different doses of FPC (90 and 180 mg/kg). Compared to untreated rats, both FPC doses significantly increased levels of “good” cholesterol (HDL-C and ApoA) while decreasing levels of “bad” cholesterol (LDL-C, ApoB, triglycerides, and total cholesterol). FPC also improved the body’s natural antioxidant defenses (SOD, CAT, GSH-Px) and reduced markers of inflammation (TNF-α, IL-6, MDA). Furthermore, FPC increased the activity of genes involved in removing cholesterol from the blood (LDLR, PPARα) and decreased the activity of genes involved in fat storage and inflammation (IL-6, TNF-α, ACC, SREBP-1C) in the liver. These findings suggest that FPC protects against atherosclerosis (artery hardening) in rats fed a high-fat diet. This effect may be due to its ability to improve antioxidant function, regulate blood fat levels, and reduce inflammation. The researchers believe the specific flavonoids rutin, luteolin-7-O-glucoside, and quercitrin in FPC may be responsible for these beneficial effects.

## 17 High blood viscosity syndrome

Blood flow properties are crucial for delivering oxygen and nutrients to tissues throughout the body. These properties are influenced by factors like blood viscosity and red blood cell deformability. Several studies have shown a connection between impaired blood flow and aging in humans. This can manifest as increased viscosity of plasma and whole blood, reduced red blood cell flexibility, and a tendency for red blood cells to clump together (Simmonds et al., 2013).

### 17.1 Ascorbic acid and diquertin

This section focuses on the potential benefits of two berry metabolites, diuretic and ascorbic acid (likely referring to vitamin C), on blood flow properties in a rat model of cerebral ischemia (stroke). Researchers, including Plotnikov et al., have investigated these effects. The study involved male Wistar rats weighing 200–250 g. Blood samples were collected from the carotid artery. Diquertin, a metabolite potentially derived from the berries, significantly reduced blood viscosity (by 12%) and erythrocyte aggregation (clumping) while increasing their deformability (flexibility) by 22%. These findings suggest that diquertin may improve blood flow by reducing viscosity and enhancing red blood cell function. *In vitro* (laboratory) experiments, diquertin also displayed similar effects, further supporting its potential role in improving blood flow properties. Additionally, the study explored the combined effects of diquertin and ascorbic acid *in vivo* (in living rats) during cerebral ischemia. This combination appeared to lessen the severity of high blood viscosity syndrome, potentially contributing to improved blood flow and red blood cell function (Plotnikov et al., 2003).

## 18 Cognitive loss

A body of literature presents the adverse influences of aging on cognitive function and memory. Age is one of the most decisive risk factors for cognitive disorders (Siebert et al., 2018). Besides routine chemical medications, other therapeutic choices, especially metabolites of plant origin, should be explored for cognitive disorders therapy (Park et al., 2012). Lifelong use of flavonoid-rich fruits, namely, berries, can uniquely improve age-related cognition decline (Thiébot, 1985).

### 18.1 Ellagic acid

Ellagic acid (EA), a natural metabolite abundant in red berries like strawberries, blueberries, and raspberries (Reeta et al., 2009), has been investigated for its potential to combat age-related cognitive decline. Studies suggest that EA exerts various beneficial effects, including ROS, chelating iron, and exhibiting antidepressant, anxiolytic, antioxidant, and anti-inflammatory properties (Festa et al., 2001; Buniatian, 2003; Townsend and Tew, 2003; Mansouri et al., 2013; Mansouri et al., 2016). Additionally, EA may inhibit tumor formation and the production of inflammatory molecules in immune cells. Research has explored the neuroprotective potential of EA in various neurological conditions. It may be beneficial in stroke, traumatic brain injury, and brain damage caused by other factors (Uzar et al., 2012; Rafieirad and Ghasemzadeh, 2014; Farbood et al., 2015). Furthermore, EA shows promise in reducing the toxicity of amyloid beta-42 (Aβ42), a protein implicated in AD, and preventing cell death in nerve cells (Pavlica and Gebhardt, 2005; Hartman et al., 2006; Feng et al., 2009). These findings suggest that EA could be a valuable therapeutic strategy for managing neurodegenerative diseases. Supporting this potential, Mansouri et al. demonstrated that EA administration significantly improved cognitive function in animal models treated with scopolamine and diazepam, medications known to impair memory (Mansouri et al., 2016). These results highlight EA as a promising candidate for the treatment of cognitive disorders.

## 19 Parkinson disease

### 19.1 Flavonoids

Baicalein has been tested in several different rodent models of PD. This includes the MPTP model with both intraperitoneal (IP) injection in rats (40 mg/kg/day) (Hung et al., 2016) and oral administration in mice (200 mg/kg/day) (Cheng et al., 2008), and the rotenone model using IP injection in rats (2.5 mg/kg/day) (Zhang et al., 2017). In all cases, baicalein moderated the loss of dopaminergic neurons, while also reducing behavioral disorders and markers of oxidative stress in the mouse MPTP model and the rotenone model (Rafieirad and Ghasemzadeh, 2014). Notably, it also diminished markers of inflammation in the MPTP models (Cheng et al., 2008; Hung et al., 2016). Several other flavones have also shown promise in PD models. Apigenin was administered using IP injection (10 and 20 mg/kg/day) in the rotenone model in rats (Anusha et al., 2017). Likewise, chrysin (10 mg/kg/day) was given orally in the 6-hydroxydopamine model in mice (Goes et al., 2018), nobiletin (only 10 mg/kg/day) was given orally in the MPTP model in rats (Jeong et al., 2015), and morin (50 mg/kg) was injected daily via IP in the MPTP model in mice (Lee et al., 2016). All five flavones helped preserve dopaminergic neurons and reduced markers of inflammation. Furthermore, apigenin, chrysin, and luteolin prevented the toxin-induced reduction in neurotrophic factor gene expression. These three flavones, along with morin, also ameliorated the behavioral changes caused by the toxins (Lee et al., 2016).

Quercetin also lessens rotenone-induced behavioral alterations and loss of tyrosine hydroxylase immunoreactivity in both the SN and the striatum. Tyrosine hydroxylase immunoreactivity is a marker for the integrity of the nigrostriatal pathway. Oral administration of quercetin (100 and 200 mg/kg/day) before the administration of the toxin enhanced motor function in MPTP-treated mice in a dose-dependent way (Lv et al., 2012), which correlated with a significant gain in striatal dopamine levels and a significant reduction in a marker of lipid peroxidation. Recently, quercetin was tested in the MitoPark transgenic mouse model of PD (Ay et al., 2017). These mice have a limited disruption of mitochondrial transcription factor A, specifically in dopaminergic neurons, and recapitulate several aspects of human PD, including adult beginning, slow defect of motor function, and deterioration in nigrostriatal pathway (Ekstrand and Galter, 2009). Oral administration of quercetin (25 mg/kg/day) to these mice for 6–8 weeks beginning at 12 weeks of age moderately but significantly lessened behavioral deficits, striatal dopamine loss and nigrostriatal degeneration. The quercetin glycoside rutin was also tested in the oxidopamine model in rats, where daily IP injection (10 and 30 mg/kg) was shown to partially lessen motor deficits during treatment launched 3 weeks before administration of the toxin. This correlated with a moderate but significant increase in striatal dopamine levels as well as an increase in brain glutathione levels. In contrast, markers of both lipid and protein oxidation were reduced (Khan et al., 2012). Oxidative stress is critical within the progressive neurodegeneration in Parkinson’s disease. Over the top responsive oxygen species advance cell death pathways such as apoptosis, and cytoplasmic and autophagic cell death proposing that unused restorative choices ought to include focusing on oxidative stress (Panchal and Brown, 2022).

Although the flavonol fisetin (20 mg/kg/day) did not show positive effect in the same oxidopamine study in rats where quercetin also failed to show a beneficial effect (Zbarsky et al., 2005), a more recent study using MPTP-treated mice found that oral administration of fisetin (10–25 mg/kg/day) before treatment with the toxin dose-dependently increased striatal dopamine levels and largely prevented the loss of tyrosine hydroxylase immunoreactivity in the striatum (Maher, 2017). Both the flavonol myricetin (2.5 µg/day) and its glycoside myricitrin (60 mg/kg/day) maintained tyrosine hydroxylase -positive neurons in the oxidopamine model in rodents when operated by myricitrin injection. Myricitrin also lessens markers of inflammation and improves motor function, while myricetin increases dopamine levels (Kujawska and Jodynis-Liebert, 2018).

Flavanones have also shown benefits in PD models, including naringenin in the MPTP model in mice (Mani et al., 2018) and the oxidopamine model in rats (oral; 50 mg/kg/day) (Zbarsky et al., 2005) and hesperetin in the oxidopamine model in rats (Kiasalari et al., 2016). Oral leadership of naringenin (25, 50, and 100 mg/kg/day) enlarged dopamine levels and diminished the loss of tyrosine hydroxylase immunoreactivity while also lowering markers of inflammation and oxidative stress (Mani et al., 2018). The glycoside of naringenin, naringin (IP; 80 mg/kg/day), was tested using both pre-and post-treatment in the oxidopamine model in rats (Kim et al., 2016). While pre-treatment protected *versus* the toxin-induced loss of DA neurons and averted microglial activation, post-treatment had no beneficial effects (Kim et al., 2016). Oral administration of hesperetin (50 mg/kg/day) diminished oxidopamine-induced behavioral deficits and prevented the loss of DA neurons (Kiasalari et al., 2016). These effects correlate with reductions in some, but not all, inflammatory markers and lower indices of oxidative stress, and increases in glutathione levels. Similarly, oral delivery of the hesperetin glycoside hesperidin (50 mg/kg/day) reversed behavioral disorders, decreased striatal dopamine loss, and reduced oxidative stress markers in the brain of oxidopamine-treated old mice (Antunes et al., 2014).

In summary, a wide range of avonoids have shown significant benefits in various rodent toxin-based models of PD. Where they have been studied, they reduced markers of inflammation and oxidative stress and increased markers of neurotrophic factor signaling. Together, these effects resulted in the prevention of nerve cell death and the reduction in behavioral disorders. In addition, many prevented the expansion of -synuclein, a protein implicated in neuronal damage and death in Parkinson’s disease. Therefore, similar to AD, the avonoids appear to have multiple targets in the PD models, further supporting the idea that multi-targeted metabolites are likely to offer the best treatments for this neurodegenerative disease. However, as genetic models of PD become more reproducible, it will be important to test some of the most promising avonoids in these models to provide further evidence of potential clinical efficacy.

## 20 Neurogenesis

While neurogenesis, the process of generating new neurons, is most active during embryonic development and early postnatal growth, research suggests it persists throughout adulthood in specific regions of the mammalian CNS, including humans. This ongoing neurogenesis is believed to contribute to both normal brain function and the potential for recovery from neurological disorders. However, the rate of neurogenesis naturally declines with age, raising questions about its therapeutic potential in neurodegenerative diseases (Galvan and Jin, 2007).

### 20.1 Flavonoids

Shengkai et al. investigated the potential of flavonoids derived from *cutellaria baicalensis Georgi* (Scrophulariaceae) to promote neurogenesis and improve memory in male mice (Zbarsky et al., 2005). The study focused on the bdnf-erk-creb signaling pathway, believed to be crucial for these processes. The researchers established an AD model in the mice by injecting a combination of amyloid beta protein (Aβ25-35), aluminum trichloride, and human growth factor into their ventricles. To assess the effects of the flavonoids (SSF), the researchers measured learning and memory function in the mice. This Aβ injection impaired the rats’ memory and reduced the levels of a protein called Ki67 in the hippocampus, a brain region crucial for memory. Interestingly, the Aβ injection also increased the levels of mRNA and proteins involved in a signaling pathway essential for neurogenesis, known as the BDNF-ERK-CREB pathway. The study also investigated a substance called SSF. When given to rats along with the Aβ injection, SSF significantly improved their memory function. Furthermore, SSF lowered the levels of the Ki67 protein but also regulated the mRNA and protein levels of molecules in the BDNF-ERK-CREB pathway that were abnormally increased by Aβ (Shengkai et al., 2022). In conclusion, the Aβ injection impaired memory, suppressed neurogenesis, and disrupted the BDNF-ERK-CREB signaling pathway. SSF, however, appeared to counteract these negative effects, potentially by regulating the activity of this signaling pathway. Their findings suggest that SSF may be a potential therapeutic candidate for promoting neurogenesis and improving memory function, possibly through modulation of the bdnf-erk-creb signaling pathway.

## 21 Amyotrophic lateral sclerosis

### 21.1 Flavonoids

Xu et al. investigated the potential neuroprotective effects of epigallocatechin-3-gallate (EGCG) in mice genetically predisposed to amyotrophic lateral sclerosis (ALS). The study involved two groups of mice: one with the ALS-causing gene mutation (SOD1-G93A) and another healthy control group (wild-type). Within each group, the mice were further divided into those receiving EGCG orally (10 mg/kg) and those receiving a control solution. The study showed that giving EGCG to the ALS mice before symptoms appeared significantly delayed the onset of the disease and extended their lifespan. Furthermore, the EGCG-treated mice had several positive changes in their spinal cords, including a greater number of motor neurons (the nerve cells that degenerate in ALS), reduced activation of immune cells called microglia, and lower levels of proteins involved in inflammation and cell death (Xu et al., 2006). These findings suggest that EGCG may have multiple therapeutic benefits in this ALS mouse model.

## 22 Neurodevelopmental disorders

The development of NDDs is widespread in older adults (Tsunemi and La Spada, 2012). There are many studies that proposed that age-related NDDs could result in cell aging and lead to apoptosis. During the process of human aging, ROS development matters the most (Nunomura et al., 2012; Maher, 2019b). It is proposed that AMPK can be activated by existing flavonoids in the diet (Gasparrini et al., 2016). By decreasing mitochondria function due to age-related reductions in AMPK activity (Reznick et al., 2007) simply results in impairment from oxidative stress. Functional activity of SIRT1 is induced by AMPK stimulation (Salminen and Kaarniranta, 2012), and mitochondrial biogenesis is stimulated by PGC-1α (Nakahata et al., 2009).

### 22.1 Flavonoids

**FIGURE 2:**
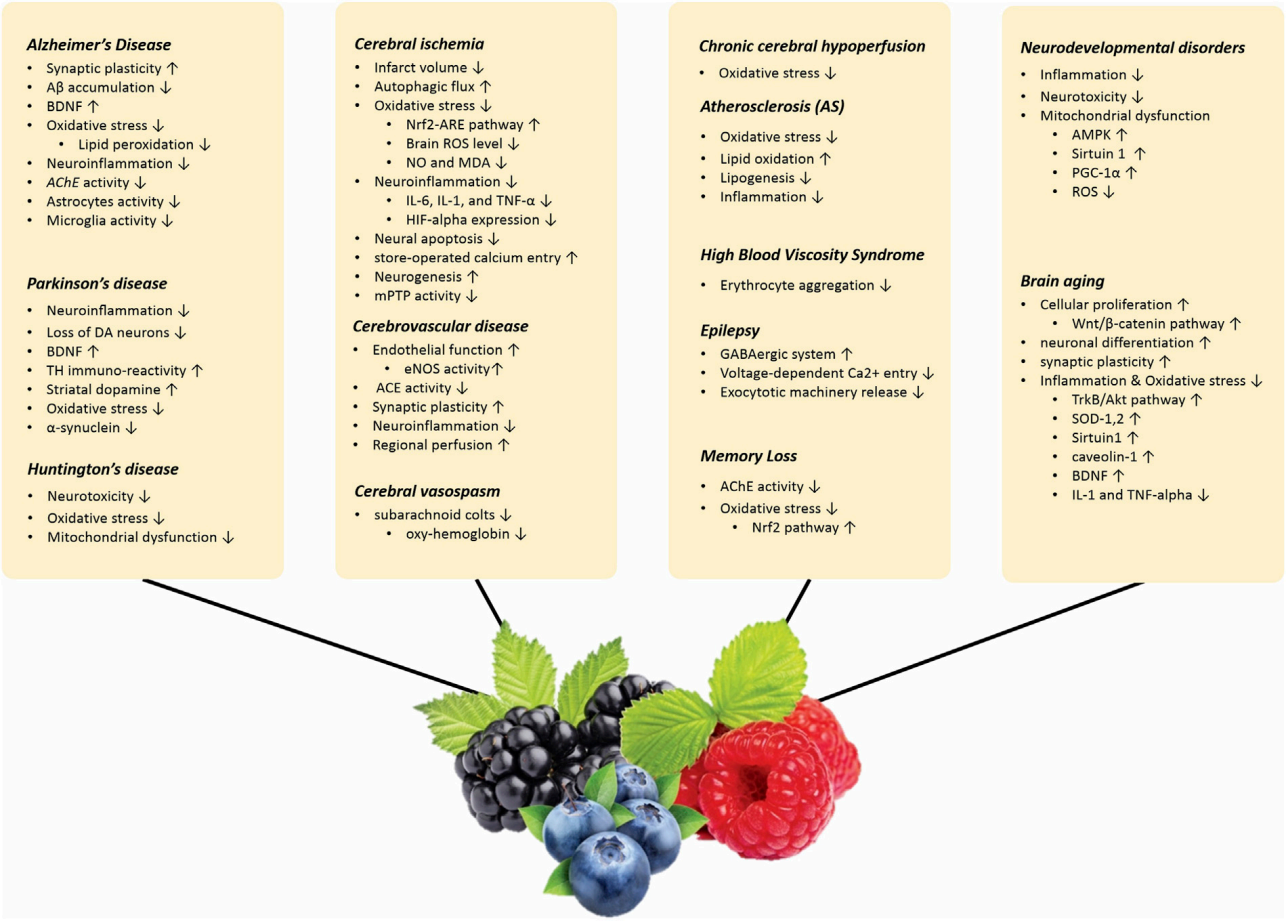
The underlying mechanism of therapeutic effects of Berries on Age-related neurological conditions; Alzheimer’s disease, Parkinson’s disease, Huntington’s disease, Cerebral ischemia, Cerebrovascular diseases, Atherosclerosis, High Blood Viscosity Syndrome, Epilepsy, Memory loss, Neurodevelopmental disorders, Brain aging. symbols " ↑ " and "↓" are to demonstrate increase/stimulation and decrease/suppression respectively. Abbreviations: Aβ: amyloid-β, BDNF: Brain-derived neurotrophic factor, AChE: acetylcholinesterase, DA: Dopaminergic, ROS: Reactive oxygen species, NRF: The nuclear factor-erythroid–related factor 2, ARE: antioxidant responsive element, NO: Nitric oxide, MDA: Malondialdehyde, IL: Interleukin, TNF: Tumor necrosis factor, HIF: Hypoxia-inducible factor, mPTP: Mitochondrial permeability transition pore, eNOS: Endothelial nitric oxide synthase, ACE: Angiotensin-converting enzyme, AMPK: Adenosine monophosphate (AMP)-activated protein kinase, PGC-1α: Peroxisome proliferator-activated receptor gamma coactivator 1-alpha, TrKB: Tropomyosin-related kinase receptor B, Akt: Protein kinase B.

In a recent survey, quercetin, luteolin, and kaempferol are introduced as the main active flavonoids in *Ginkgo biloba* L. (Yang S. et al., 2019). Quercetin can avert inflammatory mediator’s development in older people (Qureshi et al., 2011). The cytotoxic activity of 7-ketocholesterol is reduced by quercetin along with apigenin. The dysfunction of mitochondrial is averted by these flavonoids by modulating AMPK, SIRT1, and PGC-1α gene expression (Xiong et al., 2018; Yammine et al., 2020). *Humulus japonicas* Siebold & Zucc. contain luteolin, luteolin-7-glucoside, quercetin and quercitrin; Thus, it can be used to provide resources for the production of pharmacological or nutraceutical therapies (Sung et al., 2015). Ingestion of strawberries included flavonoid content is available by ingesting strawberries which is related to *in vivo* upregulation of AMPK and reduced levels of intracellular ROS during aging (Giampieri et al., 2017; Lewinska et al., 2020). (Figure 2 shows the underlying mechanism of therapeutic effects of berries.)

## 23 Conclusion

In conclusion, a growing body of evidence suggests that berries, including blackberry, blueberry, and mulberry, harbor a diverse array of bioactive metabolites with potential neuroprotective properties. These metabolites encompass flavonoids, carotenoids, vitamins C and E, and anthocyanins. Findings from pre-clinical studies indicate their ability to modulate processes implicated in neurodegenerative diseases (Alzheimer’s and Parkinson’s diseases), cerebrovascular accidents, amnesia, and age-related cognitive decline. For example, ascorbic acid (vitamin C) demonstrates potent antioxidant activity, potentially mitigating free radical production and subsequent neuronal damage during ischemic stroke. Similarly, ellagic acid (EA) may offer protection against memory deficits by preventing the reduction of key signaling molecules in the brain. Moreover, preliminary evidence suggests that certain berry metabolites, like gallic acid (GA) and lactic acid, may exert neuroprotective effects through anti-inflammatory mechanisms and improved cellular resilience following injuries. However, it is crucial to acknowledge limitations in the current body of research. The majority of studies have been conducted in animal models, necessitating further investigations to elucidate the precise mechanisms underlying the observed neuroprotective effects of these berry metabolites. Additionally, the specific contributions of individual metabolites within berries and their potential synergistic interactions require further exploration. Future research priorities should focus on (Campisi et al., 2019): Clinical trials (Harman, 2001), Mechanistic studies, and (Li et al., 2021) Dosage optimization. By addressing these limitations and pursuing these research avenues, we can gain a deeper understanding of the potential of berries to promote brain health and ultimately translate this knowledge into evidence-based dietary or therapeutic recommendations.
