# Supplementary material for: Therapeutic potential of berries in age-related neurological disorders
Source: Front Pharmacol. 2024 May 9;15:1348127. doi: 10.3389/fphar.2024.1348127 (PMC11112503; doi:10.3389/fphar.2024.1348127)
Supplement: Supplementary file 1 [file Table1.DOCX]

| **Author (reference) Year** | **Design** | **Number of Participants** | **Disease** | **Berry or its active constituents** | **Dose** | **Outcomes** |
| --- | --- | --- | --- | --- | --- | --- |
| Natalie S. Werner (1), 2009 | Randomized, placebo controlled double blind study | 80  Intervention=40  Placebo=40 | Cognitive function in the elderly aged 50-80 y/o | D-camphor crataegus berry extract | 25drops of Korodins in a single dose (1 drop = 1 mg D-camphor and 38.62 mg crataegus berry extract) | Significant immediate and short-time improvement of cognitive performance (Processing time in the Number Connection-Test, Digit-Symbol-Test), cerebral metabolism and direct effects of neural activation processes |
| Adrian R. Whyte, & Nancy Cheng (2), 2019 | Single-blind, randomized, placebo controlled, between-subjects design | 40  Intervention=20  Placebo=20 | Cognitive Function in young healthy adults aged 20–30 y/o | Flavonoid-rich mixed berries containing strawberries, blueberries, blackberries, and raspberries | A single dose of 400 mL ‘smoothie’ consisting 75g each of whole strawberries, blueberries, blackberries, and raspberries, blended with 100 mL water | Significant improvement of executive function (EF) and cognitive functions including Modified Attention Network Task (MANT), and Task Switch Task (TST) Over 6 h |
| Adrian R. Whyte (3), 2021 | Randomized investigator- and subject- blinded, 2-arm, placebo-controlled, cross-over trial | 35 | Cognitive performance in cognitively healthy individuals aged 40–65 y/o | Wild blueberry (WBB) | A single dose WBB beverage(348g) consisting 25g freeze-dried whole WBB powder (~ 1-cup fresh weight) with a breakfast meal on two occasions separated by at least 7 days | Significant acute enhancement of cognitive function, episodic memory on the memory-related Auditory Verbal Learning Task (AVLT) word recognition, and EF on the Go/No-Go task |
| Carol L. Cheatham (4), 2022 | Double-blind, randomized placebo-controlled trial | 86  Intervention=44  Placebo=42 | Mild cognitive decline in older adults aged 65–80 y/o | WBB | 35g/day of lyophilized WBB powder for 6months | Improved cognitive aging sequelae by improving the speed of information processing in older adults on the Cambridge Neurological Test Automated Battery (CANTAB) |
| Marshall G. Miller (5), 2018 | Randomized, double-blind, placebo-controlled trial | 37  Intervention=19  Placebo=19 | Cognition function among older adults aged 60-75 y/o | Blueberry | Freeze-dried blueberry (24 g/day, equivalent to 1 cup of fresh blueberries) for 90 days | Significant fewer repetition errors in the California Verbal Learning test (CVLT), and reduced switch cost on the TST |
| Anthony.W. WATSON (6), 2012 | Double blind placebo-controlled study | 35 | Cognitive performance and mood in healthy young participants | Two berry fruit extracts | A powdered berry extract (521mg polyphenols /60kg body weight), juiced extract (528mg polyphenols /60 kg) | Improved attention task performance, increased accuracy during a rapid visual information processing task, and improved reaction times during the digit vigilance task |
| Grant Rutledge (7), 2021 | Randomized, double-blind, placebo-controlled, 2-arm, parallel study | 38  Intervention=19  Placebo=19 | Cognition function in healthy older adults aged 60–75 y/o | Blueberry and strawberry polyphenols and anthocyanins | 24 g/day freeze-dried strawberry or lyophilized, cultivated blueberry (equivalent to 1 cup/day of BB; 12g powder in ~1 cup water taken with each morning and evening meal) for 90-days | Significant alters levels of circulating phenolic compounds related to improvements in cognition, EF, and memory on the TST, and the CVLT-II |
| Elizabeth E. Devore (8), 2012 | Large, prospective cohort of older women in the Nurses’ Health Study | 16,010 | Cognitive decline in older women aged ≥70 years | Blueberries and strawberries | Blueberries serving ($\sim\frac{1}{2}$cup) /week ≥1, and strawberries servings/ week ≥2 | Significant slower rates of cognitive decline, and delay cognitive aging by up to 2.5years in older women on the Telephone Interview of Cognitive Status |
| Wipawee Thukham-mee (9), 2020 | Randomized, Placebo-Controlled, Crossover Study | 46  Intervention=23  Placebo=23 | Cognitive function of healthy children aged 6–12 y/o | Mulberry | Single administration of mulberry milk containing mulberry 10 g, each serving = 50 mL | Improvement of attention and cognitive function on the auditory odd ball paradigm of event-related potential, working memory on a computerized battery test, decreased response time of digit updating at 1.5 and 3 hours after dosing, and decreased response time of picture updating at 3 hours after dosing |

**References:**

1. Werner NS, Duschek S, Schandry R. D-camphor-crataegus berry extract combination increases blood pressure and cognitive functioning in the elderly - A randomized, placebo controlled double blind study. Phytomedicine. 2009;16(12):1077-82.

2. Whyte AR, Cheng N, Butler LT, Lamport DJ, Williams CM. Flavonoid-rich mixed berries maintain and improve cognitive function over a 6 h period in young healthy adults. Nutrients. 2019;11(11).

3. Whyte AR, Rahman S, Bell L, Edirisinghe I, Krikorian R, Williams CM, et al. Improved metabolic function and cognitive performance in middle-aged adults following a single dose of wild blueberry. European Journal of Nutrition. 2021;60(3):1521-36.

4. Cheatham CL, Canipe LG, Millsap G, Stegall JM, Chai SC, Sheppard KW, et al. Six-month intervention with wild blueberries improved speed of processing in mild cognitive decline: a double-blind, placebo-controlled, randomized clinical trial. Nutritional Neuroscience. 2022:1-15.

5. Miller MG, Hamilton DA, Joseph JA, Shukitt-Hale B. Dietary blueberry improves cognition among older adults in a randomized, double-blind, placebo-controlled trial. European Journal of Nutrition. 2018;57(3):1169-80.

6. Watson AW, Okello EJ, Brooker HJ, Lester S, McDougall GJ, Wesnes KA. The impact of blackcurrant juice on attention, mood and brain wave spectral activity in young healthy volunteers. Nutritional Neuroscience. 2019;22(8):596-606.

7. Rutledge G, Miller M, Sandhu A, Edirisinghe I, Burton-Freeman B, Shukitt-Hale B. Berry Phenolics Are Associated With Cognitive Enhancement in Blueberry- and Strawberry-Supplemented Older Adults. Current Developments in Nutrition. 2021;5(Supplement_2):921-.

8. Devore EE, Kang JH, Breteler MM, Grodstein F. Dietary intakes of berries and flavonoids in relation to cognitive decline. Annals of neurology. 2012;72(1):135-43.

9. Thukham-mee W, Wattanathorn J, Kirisattayakul W, Wannanon P. Effect of Single Administration of Mulberry Milk on the Cognitive Function of 6–12-Year-Old Children: Results from a Randomized, Placebo-Controlled, Crossover Study. Oxidative Medicine and Cellular Longevity. 2020;2020:6123759.
