# Supplementary material for: Therapeutic potential of berries in age-related neurological disorders
Source: Front Pharmacol. 2024 May 9;15:1348127. doi: 10.3389/fphar.2024.1348127 (PMC11112503; doi:10.3389/fphar.2024.1348127)
Supplement: Supplementary file 2 [file Table2.docx]

| **Table S2. Impact of berries in various age-related neurological disorders: insights from in vivo, in vitro, and clinical studies**. | | | | | | | | | | | | |
| --- | --- | --- | --- | --- | --- | --- | --- | --- | --- | --- | --- | --- |
| Improve antioxidant level | | Neuroprotection | Improve learning and memory | Groups, Methods, and Doses | | | | Models | Type of Berries | Type of Study | Disease | Author/Year |
|  |  |  |  | Time of measurments | Duration | Treatment | Group |  |  |  |  |  |
| 🗸 | |  | 🗸 | eight  groups (ten mice per group) | | | | Pb-induced mice | ME | In vivo | AD | Yao et al. (1)/2014 |
|  | |  | 🗸 | 45 min, 24 h (9th day) | 8 days | Saline | I(Control - Young Mice) | Swiss mice | Ascorbic Acid | In vivo | AD | Parle et al. (2) /2003 |
|  |  |  |  | 45 min, 24 h | - | Scopolamine (0.4 mg/kg) | II |  |  |  |  |  |
|  |  |  |  | 45 min, 24 h | - | Diazepam (1 mg/kg) | III |  |  |  |  |  |
|  |  |  |  | 8th day, 24 h (9th day) | 8 days | Piracetam (200 mg/kg) | IV |  |  |  |  |  |
|  |  |  |  | 8th day, 24 h (9th day) | 8 days | Piracetam (400 mg/kg) | V |  |  |  |  |  |
|  |  |  |  | 45 min after Scopolamine, 24 h (9th day) | 8 days (Piracetam) then Scopolamine at 60 min before test | Piracetam (400 mg/kg) + Scopolamine (0.4 mg/kg) | VI |  |  |  |  |  |
|  |  |  |  | 3rd day, 24 h (4th day) | 3 days | Ascorbic Acid (60 mg/kg) | VII |  |  |  |  |  |
|  |  |  |  | 3rd day, 24 h (4th day) | 3 days | Ascorbic Acid (120 mg/kg) | VIII |  |  |  |  |  |
|  |  |  |  | 8th day, 24 h (9th day) | 8 days | Ascorbic Acid (60 mg/kg) | IX |  |  |  |  |  |
|  | |  |  | 8th day, 24 h (9th day) | 8 days | Ascorbic Acid (120 mg/kg) | X |  |  |  |  |  |
|  |  |  |  | 45 min after Scopolamine, 24 h (4th day) | 3 days (Ascorbic Acid) then Scopolamine at 60 min before test | Ascorbic Acid (60 mg/kg) + Scopolamine (0.4 mg/kg) | XI |  |  |  |  |  |
|  |  |  |  | 45 min after Diazepam, 24 h (4th day) | 3 days (Ascorbic Acid) then Diazepam at 60 min before test | Ascorbic Acid (60 mg/kg) + Diazepam (1 mg/kg) | XII |  |  |  |  |  |
|  |  |  |  | 45 min after injection on 8th day, 24 h (9th day) | 8 days | Saline | XIII (Control - Aged Mice) |  |  |  |  |  |
|  |  |  |  | 8th day, 24 h (9th day) | 8 days | Piracetam (400 mg/kg) | XIV |  |  |  |  |  |
|  |  |  |  | 3rd day, 24 h (4th day) | 3 days | Ascorbic Acid (60 mg/kg) | XV |  |  |  |  |  |
|  |  |  |  | 3rd day, 24 h (4th day) | 3 days | Ascorbic Acid (120 mg/kg) | XVI |  |  |  |  |  |
|  |  |  |  | 8th day, 24 h (9th day) | 8 days | Ascorbic Acid (60 mg/kg) | XVII |  |  |  |  |  |
|  |  |  |  | 8th day, 24 h (9th day) | 8 days | Ascorbic Acid (120 mg/kg) | XVIII |  |  |  |  |  |
|  |  |  |  | 3rd day, 24 h (4th day) | 3 days | Saline | XIX (Control - Young Mice) |  |  |  |  |  |
|  |  |  |  | 3rd day, 24 h (4th day) | 3 days | Ascorbic Acid (60 mg/kg) | XX |  |  |  |  |  |
|  |  |  |  | 3rd day, 24 h (4th day) | 3 days | Ascorbic Acid (120 mg/kg) | XXI |  |  |  |  |  |
|  |  |  |  | 45 min after injection (on day 2) | - | Scopolamine (0.4 mg/kg) | XXII |  |  |  |  |  |
|  |  |  |  | After training sessions (days 2 & 3), 45 min after Scopolamine (day 4) | 3 days (Ascorbic Acid) then Scopolamine at 45 min before test (on day 4) | Ascorbic Acid (60 mg/kg) + Scopolamine (0.4 mg/kg) | XXIII |  |  |  |  |  |
|  |  |  |  | 3rd day, 24 h (4th day) | 3 days | Saline | XXIV (Control - Aged Mice) |  |  |  |  |  |
|  |  |  |  | 3rd day, 24 h (4th day) | 3 days | Ascorbic Acid (60 mg/kg) | XXV |  |  |  |  |  |
|  |  |  |  | 3rd day, 24 h (4th day) | 3 days | Ascorbic Acid (120 mg/kg) | XXVI |  |  |  |  |  |
|  |  |  |  | Observed for emotional state and writhing syndrome | - | Observation Only | XXVII-XXVIII |  |  |  |  |  |
|  | |  | 🗸 | NR | NR | BB extracts (Vaccinium uliginosum L) | AD+BB | APP/PS1 transgenic mice | ellagic acid | In vivo | AD | Tan et al. (3)/2017 |
|  |  |  |  | NR | NR | Saline water (0.9%) | AD (Positive Control) |  |  |  |  |  |
|  |  |  |  | NR | NR | Saline water (0.9%) | CT (Negative Control) |  |  |  |  |  |
| 🗸 | |  | 🗸 | 3rd week post-surgery | - | - | Sham | Rat | ellagic acid | In vivo | AD | Kiasalari et al. (4) /2017 |
|  |  |  |  | 3rd week post-surgery | 1 week | 100 mg/kg | Ellagic Acid-pretreated Sham |  |  |  |  |  |
|  |  |  |  | 3rd week post-surgery | - (single injection) | 10 μg | Aβ25-35 |  |  |  |  |  |
|  |  |  |  | 3rd week post-surgery | 1 week (ellagic acid) | 10 mg/kg ellagic acid + 10 μg Aβ25-35 | Ellagic Acid + Aβ25-35 |  |  |  |  |  |
|  |  |  |  | 3rd week post-surgery | 1 week (ellagic acid) | 50 mg/kg ellagic acid + 10 μg Aβ25-35 | Ellagic Acid + Aβ25-35 (medium dose) |  |  |  |  |  |
|  |  |  |  | 3rd week post-surgery | 1 week (ellagic acid) | 100 mg/kg ellagic acid + 10 μg Aβ25-35 | Ellagic Acid + Aβ25-35 (high dose) |  |  |  |  |  |
|  | |  | 🗸 | - Before treatment (baseline)/ Throughout behavioral tests/ 24 h before sacrifice | 2 months | 25 mg/kg Quercetin | 1 (SAMP8) | SAMP8 and SAMR1 mice | quercetin | In vivo | AD | Puerta et a. (5)/ 2017 |
|  |  |  |  | - Before treatment (baseline)/ Throughout behavioral tests/ 48 h before sacrifice | 2 months | 25 mg/kg Quercetin-loaded nanoparticles | 2 (SAMP8) |  |  |  |  |  |
|  |  |  |  | - Before treatment (baseline)/ Throughout behavioral tests | 2 months | 1 mL Saline | 3 (Control - SAMP8) |  |  |  |  |  |
|  |  |  |  | - Before treatment (baseline)/ Throughout behavioral tests | 2 months | 1 mL Saline | 4 (Control - SAMR1) |  |  |  |  |  |
|  | |  | 🗸 | NR | 3 months (every 48 hours) | 25 mg/kg | Quercetin | Homozygous 3xTg-AD and non-transgenic mice | quercetin | In vivo | AD | Sabogal-Guáqueta et al. (6) /2015 |
|  |  |  |  | NR | 3 months (every 48 hours) | 0.1% DMSO | Control |  |  |  |  |  |
| 🗸 | |  |  | NR | 3 weeks (daily after Aβ injection) | Saline (10 ml/kg, i.p.) | Control Group | Wistar Rat | Rutin | In vivo | AD | Moghbelinejad et al. (7)/ 2014 |
|  |  |  |  | - After habituation trial (latency to enter dark compartment) - 30 minutes after acquisition trial (step-through latency) - 1 day after training (retention test - latency to enter dark compartment or 300 seconds) | 3 weeks (daily after Aβ injection) | Rutin (100 mg/kg, i.p.) | Treatment Group |  |  |  |  |  |
|  | |  | 🗸 | NR | NR | 0.9% saline | Control | C57BL/6N mice | anthocyanin-loaded polyethylene glycol-gold nanoparticles | In vivo and in vitro | AD | Ali et al. (8)/ 2016 |
|  |  |  |  | NR | 7 days (Aβ1-42 injection) | Aβ1-42 peptide (aggregated) | Aβ1-42 |  |  |  |  |  |
|  |  |  |  | NR | 14 days (after Aβ1-42 injection) | 12 μg/g/day | Aβ1-42 + Anthocyanins |  |  |  |  |  |
|  |  |  |  | NR | 14 days (after Aβ1-42 injection) | 12 μg/g/day | Aβ1-42 + Anthocyanin-loaded PEG-AuNPs |  |  |  |  |  |
| 🗸 | |  |  | NR | | | | Aβ1-42-injected mouse | anthocyanin-loaded polyethylene glycol-gold nanoparticles | In vivo and in vitro | AD | Kim et al. (9) / 2017 |
| 🗸 | |  |  | NR | Three injections with 6 hours interval between each injection | 0.5 mg/kg | Epigallocatechin-3-gallate | C57BL/6 mice | Epigallocatechin-3-gallate | In vivo | AD | Seong et al. (10)/2016 |
| 🗸 | |  | 🗸 | After behavioral tests | 3 months | Saline | Control | ICR mice | Dihydromyrcetin | In vivo | AD | Liu et al. (11)/ 2018 |
|  |  |  |  | After behavioral tests | 3 months | 250 mg Pb/L drinking water | Pb |  |  |  |  |  |
|  |  |  |  | After behavioral tests | 3 months | 250 mg Pb/L drinking water + 125 mg/kg DMH (daily oral gavage) | Pb+DHM(125 mg/kg) |  |  |  |  |  |
|  |  |  |  | After behavioral tests | 3 months | 250 mg Pb/L drinking water + 250 mg/kg DMH (daily oral gavage) | Pb+DHM(250 mg/kg) |  |  |  |  |  |
| 🗸 | |  |  | NR | 4 weeks | Saline (0.9% NaCl) | 1 (Control) | ICR mice | fiestin | In vivo | AD | Yang et al. (12)/ 2019 |
|  |  |  |  | NR | 4 weeks | Lead acetate (200 mg/L) in drinking water | 2 (Pb) |  |  |  |  |  |
|  |  |  |  | NR | 4 weeks | Lead acetate (200 mg/L) in drinking water + Fisetin (25 mg/kg b.w.) intragastrically | 3 (Pb + Fisetin) |  |  |  |  |  |
|  |  |  |  | NR | 4 weeks | Lead acetate (200 mg/L) in drinking water + Fisetin (50 mg/kg b.w.) intragastrically | 4 (Pb + Fisetin) |  |  |  |  |  |
| 🗸 | |  | 🗸 | NR | NR | Saline | Sham | Sprague-Dawley rats | Icariside II | In vivo | AD | Yin et al. (13)/ 2018 |
|  |  |  |  | NR | 28 days | 16 mg/Kg ICS II | Sham + ICS II 16 |  |  |  |  |  |
|  |  |  |  | NR | NR | Saline | BCCAO |  |  |  |  |  |
|  |  |  |  | NR | 28 days | 4 mg/Kg ICS II | BCCAO + ICS II 4 |  |  |  |  |  |
|  |  |  |  | NR | 28 days | 8 mg/Kg ICS II | BCCAO + ICS II 8 |  |  |  |  |  |
|  |  |  |  | NR | 28 days | 16 mg/Kg ICS II | BCCAO + ICS II 16 |  |  |  |  |  |
| 🗸 | |  |  | Curcumin (0.1, 1.0 and 10 μM) | | | | ICR mice, Sprague–Dawley rats, Human neuroblastoma SH-SY5Y cells | Curcumin | In vivo and in vitro | AD | Li et al. (14)/2015 |
| 🗸 | |  |  | genistein (0.5 μM) | | | | rat cortical astrocytes | Genistein | In vitro | AD | Valles et al. (15)/ 2010 |
| 🗸 | |  | 🗸 | NR | NR | None | Wild Type | APPswe/PS1dE9 mice | Genistein |  | AD | Bonet-Costa et al. (16)/ 2016 |
|  |  |  |  | NR | 3 days | 100 mg/kg/day Bexarotene | APP/PS1 |  |  |  |  |  |
|  |  |  |  | NR | 6 days | Genistein: 0.022 mg/kg/day (followed by combination) | APP/PS1 |  |  |  |  |  |
|  |  |  |  | NR | 3 days | Genistein + Bexarotene | APP/PS1 |  |  |  |  |  |
|  |  |  |  | NR | 6 days | 0.022 mg/kg/day Genistein | APP/PS1 |  |  |  |  |  |
| 🗸 | |  |  | 45 min after saline on Day 6 | 7 days | Saline | 1 (Control) | Swiss mice | Gallic acid -loaded chitosan nanoparticles | In vivo | Amnesia | Nagpal et al. (17)/ 2013 |
|  |  |  |  | 45 min after SC on Day 7 | 6 days (Saline) + SC 45 min before measurement | Saline (initial) + 0.4 mg/kg Scopolamine | 2 (SC) |  |  |  |  |  |
|  |  |  |  | 45 min after dummy NP on Day 6 | 7 days | Equivalent to Group 8 | 3 (SC þ cNP) |  |  |  |  |  |
|  |  |  |  | 45 min after GA on Day 6 | 7 days | 10 mg/kg Gallic Acid | 4 (GA) |  |  |  |  |  |
|  |  |  |  | 45 min after SC on Day 7 | 7 days (Saline & PT) + SC 45 min before measurement | Saline (initial) + 400 mg/kg Piracetam + 0.4 mg/kg Scopolamine | 5 (SC þ PT) |  |  |  |  |  |
|  |  |  |  | 45 min after SC on Day 7 | 7 days (Saline & GA) + SC 45 min before measurement | Saline (initial) + 10 mg/kg Gallic Acid + 0.4 mg/kg Scopolamine | 6 (SC þ GA) |  |  |  |  |  |
|  |  |  |  | 45 min after SC on Day 7 | 7 days (Saline & GANP) + SC 45 min before measurement | Saline (initial) + 10 mg/kg Gallic Acid nanoparticles + 0.4 mg/kg Scopolamine | 7 (SC þ GANP) |  |  |  |  |  |
|  |  |  |  | 45 min after SC on Day 7 | 7 days (Saline & cGANP) + SC 45 min before measurement | Saline (initial) + 10 mg/kg Coated Gallic Acid nanoparticles + 0.4 mg/kg Scopolamine | 8 (SC þ cGANP) |  |  |  |  |  |
|  | | 🗸 |  | NR | NR | Resveratrol (30 mg/kg body weight)+ corn oil | Sham controls | Mongolian gerbils | Resveratrol | In vivo | Cerebral Ischemia | Wang et al. (18)/2002 |
|  |  |  |  | NR | NR | None | Ischemia |  |  |  |  |  |
|  |  |  |  | 24 h after ischemic treatment | During or immediately after occlusion + 24 h later | Resveratrol (30 mg/kg body weight) | Ischemia treated with resveratrol |  |  |  |  |  |
|  | | 🗸 | 🗸 | NR | NR | Curcumin (30 mg/kg body weight)+ corn oil | Sham controls | Mongolian gerbils | Curcumin | In vivo | Cerebral Ischemia | Wang et al. (19)/2005 |
|  | |  |  | NR | NR | None | Ischemia |  |  |  |  |  |
|  | |  |  | 24 h after ischemic treatment | During or immediately after occlusion + 24 h later | Curcumin (30 mg/kg body weight) | Ischemia treated with curcumin |  |  |  |  |  |
| 🗸 | | 🗸 |  | NR | NR | AIN93G control diet | Sham/CD | C57BL/6 J mice | Sutherlandia | In vivo | Cerebral Ischemia | Chuang et al. (20) /2014 |
|  |  |  |  | NR | 30 min occlusion + 3 days reperfusion | BCCA occlusion-induced ischemia with AIN93G diet | Isch/CD |  |  |  |  |  |
|  |  |  |  | NR | 30 min occlusion + 3 days reperfusion | BCCA occlusion-induced ischemia with AIN93G diet containing 1% Sutherlandia | Isch/SD |  |  |  |  |  |
|  |  |  |  | NR | 30 min occlusion + 3 days reperfusion | BCCA occlusion-induced ischemia with AIN93G diet containing 2% elderberry | Isch/ED |  |  |  |  |  |
| 🗸 | |  | 🗸 | NR | 8 weeks | Control Diet | Control | Sprague–Dawley rat | Elderberry | In vivo | Huntington disease | Moghaddam et al. (21) /2021 |
|  |  |  |  | NR | 5 days | 3-NP (30 mg/kg i.p. for 5 days) | 3-NP |  |  |  |  |  |
|  |  |  |  | NR | 8 weeks | Control Diet + 2% ED for 8 weeks | 3-NP + ED |  |  |  |  |  |
| 🗸 | |  |  | NORT & EPM (days 19, 20, 21); Beam walk, Footprint, Hanging wire (days 1, 7, 14, 21) | 14 days (3-NP) + 21 days (Saline) | 3-NP (10 mg/kg) for 14 days + Saline (0.1% gum acacia) for 21 days (p.o.) | Disease Control (3-NP) | Wistar rats | Ellagic Acid | In vivo | Huntington disease | Sharma et al. (22)/ 2021 |
|  |  |  |  | NORT & EPM (days 19, 20, 21); Beam walk, Footprint, Hanging wire (days 1, 7, 14, 21) | 21 days (EA & 3-NP) | EA (25 mg/kg) for 21 days (p.o.) + 3-NP (10 mg/kg) daily for 14 days | Ellagic Acid (EA 25 mg/kg) |  |  |  |  |  |
|  |  |  |  | NORT & EPM (days 19, 20, 21); Beam walk, Footprint, Hanging wire (days 1, 7, 14, 21) | 21 days (EA & 3-NP) | EA (50 mg/kg) for 21 days (p.o.) + 3-NP (10 mg/kg) daily for 14 days | Ellagic Acid (EA 50 mg/kg) |  |  |  |  |  |
|  |  |  |  | NORT & EPM (days 19, 20, 21); Beam walk, Footprint, Hanging wire (days 1, 7, 14, 21) | 21 days (EA & 3-NP) | EA (100 mg/kg) for 21 days (p.o.) + 3-NP (10 mg/kg) daily for 14 days | Ellagic Acid (EA 100 mg/kg) |  |  |  |  |  |
|  |  |  |  | NORT & EPM (days 19, 20, 21); Beam walk, Footprint, Hanging wire (days 1, 7, 14, 21) | 14 days (Saline) + 21 days (Saline) | Saline (i.p.) for 14 days + Saline (0.1% gum acacia) for 21 days (p.o.) | Vehicle Control |  |  |  |  |  |
|  |  |  |  | NORT & EPM (days 19, 20, 21); Beam walk, Footprint, Hanging wire (days 1, 7, 14, 21) | 21 days (EA) | EA (100 mg/kg) for 21 days (p.o.) | Ellagic Acid per se |  |  |  |  |  |
| 🗸 |  | | 🗸 | MFE (0, 50, 100, or 200 mg/kg) for 3 weeks | | | | ICR mice and hippocampal neuronal HT-22 cell lines | Mullberry fruit extract | In vivo and in vitro | scopolamine-induced memoty-impairment | Shin et al. (23)/2019 |
| 🗸 | |  | 🗸 | Weeks 7 & 8 on diet | 10 weeks (19-21 months old) | Dried Corn (2% of diet) | Control | F344 rats | Anthocyanins | In vivo |  | Andres-Lacueva et al. (24)/2005 |
|  |  |  |  | Weeks 7 & 8 on diet | 10 weeks (19-21 months old) | Blueberries extract (2% of diet) | BBS |  |  |  |  |  |
| 🗸 | |  | 🗸 | After treatment (behavioral analysis) | 7 weeks | 0.9% saline | Control | Sprague-Dawley rats | Anthocyanins | In vivo | neuroinflammation | Rehman et al. (25)/ 2016 |
|  |  |  |  | After treatment (behavioral analysis) | 7 weeks | 100 mg/kg | D-galactose |  |  |  |  |  |
|  |  |  |  | After treatment (behavioral analysis) | 7 weeks | 100 mg/kg | Anthocyanins |  |  |  |  |  |
|  |  |  |  | After treatment (behavioral analysis) | 7 weeks | 100 mg/kg each | D-gal + Anthocyanins |  |  |  |  |  |
| 🗸 | |  | 🗸 | NR | NR | Saline | Control (Young) | Kunming mice | Anthocyanins | In vivo | Brain aging | Wei et al. (26)/ 2017 |
|  |  |  |  | NR | 8 weeks D-galactose + gavage at week 8 | D-galactose (150 mg/kg/day) + Saline (at 8 weeks) | Negative Model (Aged) |  |  |  |  |  |
|  |  |  |  | NR | 8 weeks D-galactose + gavage at week 8 | D-galactose (150 mg/kg/day) + Anthocyanins (15 mg/kg) (at 8 weeks) | Anthocyanins Low Dose |  |  |  |  |  |
|  |  |  |  | NR | 8 weeks D-galactose + gavage at week 8 | D-galactose (150 mg/kg/day) + Anthocyanins (30 mg/kg) (at 8 weeks) | Anthocyanins High Dose |  |  |  |  |  |
|  |  |  |  | NR | 8 weeks D-galactose + gavage at week 8 | D-galactose (150 mg/kg/day) + EGCG (dose not mentioned) (at 8 weeks) | Positive Control (EGCG-15) |  |  |  |  |  |
| 🗸 | |  |  | pretreated with blueberry extract (0, 0.25, 0.50, 1.0, 2.0 mg/mL) or del-3-gluc, mal-3-gluc, pterostilbene, or resveratrol (0, 1, 10, 20, and 30 μM) for 1 hour | | | | BV-2 murine microglial cells | Stilbenes and Anthocyanins | In vitro | Neuroinflamamtion | Carey et al. (27)/2013 |
| 🗸 | |  | 🗸 | NR | 10 weeks (entire study) | Saline | Control | C57BL/6 mice | ascorbic acid | In vivo | Brain aging | Nam et al. (28)/2019 |
|  | |  |  | NR | 4 weeks (after 6 weeks of D-gal) | Ascorbic Acid (150 mg/kg/day) | Ascorbic Acid |  |  |  |  |  |
|  |  |  |  | NR | 10 weeks | D-galactose (150 mg/kg/day) | D-gal |  |  |  |  |  |
|  |  |  |  | NR | 10 weeks | D-galactose (150 mg/kg/day) for 6 weeks, then co-administered with Ascorbic Acid (150 mg/kg/day) for 4 weeks | D-gal-AA |  |  |  |  |  |
|  | | 🗸 |  | 24h after treatment | 24h | 1-200 µM EGCG or 10 µM gallic acid |  | Mixed (glial / neuronal) hippocampal cells from rat fetuses | gallic acid and epigallocatechin gallate | In vitro | β-amyloid-induced toxicity | Bastianetto et al. (29)/2006 |
|  | |  | 🗸 | After 21 days: behavior tests, blood & brain tissue collection | 21 days | Saline | 1 (Caged Control) | BALB/c mice | gallic acid | In vivo and vitro | chronic restraint stress-induced anxiety and memory loss | Salehi et al. (30)/ 2018 |
|  | |  |  | After 21 days: behavior tests, blood & brain tissue collection | 21 days | Saline | 2 (Food-Water Deprived) |  |  |  |  |  |
|  |  |  |  | After 21 days: behavior tests, blood & brain tissue collection | 21 days | Saline | 3 (Chronic Restraint Stress) |  |  |  |  |  |
|  |  |  |  | 30 min before daily restraint stress for 21 days; After 21 days: behavior tests, blood & brain tissue collection | 21 days | Gallic Acid (5 mg/kg) | 4 |  |  |  |  |  |
|  |  |  |  | 30 min before daily restraint stress for 21 days; After 21 days: behavior tests, blood & brain tissue collection | 21 days | Gallic Acid (10 mg/kg) | 5 |  |  |  |  |  |
|  |  |  |  | 30 min before daily restraint stress for 21 days; After 21 days: behavior tests, blood & brain tissue collection | 21 days | Gallic Acid (20 mg/kg) | 6 |  |  |  |  |  |
|  |  |  |  | Daily for 21 days; After 21 days: behavior tests, blood & brain tissue collection | 21 days | Gallic Acid (5 mg/kg) | 7 |  |  |  |  |  |
|  |  |  |  | Daily for 21 days; After 21 days: behavior tests, blood & brain tissue collection | 21 days | Gallic Acid (10 mg/kg) | 8 |  |  |  |  |  |
|  |  |  |  | Daily for 21 days; After 21 days: behavior tests, blood & brain tissue collection | 21 days | Gallic Acid (20 mg/kg) | 9 |  |  |  |  |  |
| 🗸 | |  | 🗸 | Next day after 4 weeks treatment | 4 weeks | Saline + Saline (Control) | (i) | Rat | gallic acid | In vivo | Arsenic induced- neurotoxicity | Samad et al. (31)/ 2019 |
|  |  |  |  | Next day after 4 weeks treatment | 4 weeks | Saline + GA (50 mg/kg/ml) | (ii) |  |  |  |  |  |
|  |  |  |  | Next day after 4 weeks treatment | 4 weeks | Saline + GA (100 mg/kg/ml) | (iii) |  |  |  |  |  |
|  |  |  |  | Next day after 4 weeks treatment | 4 weeks | iAS + Saline | (iv) |  |  |  |  |  |
|  |  |  |  | Next day after 4 weeks treatment | 4 weeks | iAS + GA (50 mg/kg/ml) | (v) |  |  |  |  |  |
|  |  |  |  | Next day after 4 weeks treatment | 4 weeks | iAS + GA (100 mg/kg/ml) | (vi) |  |  |  |  |  |
| 🗸 | | 🗸 | 🗸 | NR | 8 weeks | Vehicle (0.1% DMSO) | 1 (Control) | Wistar rats | ellagic acid | In vivo | streptozotocin -induced hippocampal damage  and memory loss | Alfaris et al. (32)/2021 |
|  |  |  |  | NR | 8 weeks | EA solution (50 mg/kg/day) | 2 (Control + EA) |  |  |  |  |  |
|  |  |  |  | NR | 8 weeks | 0.1% DMSO | 3 (STZ-T1DM) |  |  |  |  |  |
|  |  |  |  | NR | 8 weeks | EA solution (50 mg/kg/day) | 4 (STZ-T1DM + EA) |  |  |  |  |  |
| 🗸 | |  | 🗸 | 12 h after last KET + Y-maze test | 24 h (5 administrations, 3 h intervals) | Saline (0.9% NaCl, i.p.) | Control (i) | Wistar rats | Gallic acid | In vivo | Ketamin-induced toxicity | Brum et al. (33)/ 2020 |
|  |  |  |  | 12 h after last KET + Y-maze test | 24 h (5 administrations, 3 h intervals) | Ketamine (KET, 10 mg/kg, i.p.) | KET (iii) |  |  |  |  |  |
|  |  |  |  | 12 h after last KET + Y-maze test | GA (13.5 mg/kg, gavage) - 3 administrations, 2 h intervals | Saline (control for GA) | GA (ii) |  |  |  |  |  |
|  |  |  |  | 12 h after last KET + Y-maze test | Ket: 24 h (5 administrations, 3 h intervals)  GA (13.5 mg/kg, gavage) - 3 administrations, 2 h intervals | Ketamine (KET, 10 mg/kg, i.p.) | KET+GA (iv) |  |  |  |  |  |
|  | |  | 🗸 | End of 21 days (after treatment period) | 21 days | None | Normal Control | Wistar rats | Ascorbic Acid | In vivo | Stress-Induced Memory Deficits | Kumar et al. (34)/2009 |
|  |  |  |  | End of 21 days (after treatment period) | 21 days | 100 mg/kg/body weight (oral) | AA |  |  |  |  |  |
|  |  |  |  | End of 21 days (after treatment period) | 21 days | Equivolume of vehicle solution (0.9% sodium chloride solution) (oral) | Vehicle Control |  |  |  |  |  |
|  |  |  |  | End of 21 days (after treatment period) | 21 days | Stressed in wire mesh restrainers for 6 hours/day | Restraint Stress |  |  |  |  |  |
|  |  |  |  | End of 21 days (after treatment period) | 21 days | Equivolume of vehicle solution (0.9% sodium chloride solution) (oral) + Stressed in wire mesh restrainers for 6 hours/day | Restraint Stress + Vehicle |  |  |  |  |  |
|  |  |  |  | End of 21 days (after treatment period) | 21 days | 100 mg/kg/body weight Ascorbic Acid (oral) + Stressed in wire mesh restrainers for 6 hours/day | Restraint Stress + Ascorbic Acid |  |  |  |  |  |
|  | | 🗸 |  | (30 min, 1 hour, 2 hours, and 4 hours) after the intraperitoneal (IP) administration of the flavonoid solutions | 30 min, 1 hour, 2 hours, and 4 hours | NR | NR | Sprague-Dawley  rats | Quercetin, fisetin and catechin | In vivo | Focal ischemia | Rivera et al. (35)/2004 |
| 🗸 | |  |  | 24 h after reperfusion (neurological damage evaluation, brain tissue collection) | 7 days | Water | Normal | Kunming mice | Abelmoschus esculentus L. | In vivo | Transient Cerebral Ischemia-Reperfusion Injury | Luo et al. (36)/2018 |
|  |  |  |  | 24 h after reperfusion (neurological damage evaluation, brain tissue collection) | 7 days | Water | Model |  |  |  |  |  |
|  |  |  |  | 1 hour after final dose (anesthesia), 24 h after reperfusion (neurological damage evaluation, brain tissue collection) | 7 days (daily) | AFF (300 mg/kg) | High Dose AFF |  |  |  |  |  |
|  |  |  |  | 1 hour after final dose (anesthesia), 24 h after reperfusion (neurological damage evaluation, brain tissue collection) | 7 days (daily) | AFF (150 mg/kg) | Medium Dose AFF |  |  |  |  |  |
|  |  |  |  | 1 hour after final dose (anesthesia), 24 h after reperfusion (neurological damage evaluation, brain tissue collection) | 7 days (daily) | AFF (75 mg/kg) | Low Dose AFF |  |  |  |  |  |
|  | | 🗸 |  | Daily weight | 5 weeks (3 to 8 weeks old) | Soy-based chow (A03 diet) | Soy-based Diet | Wistar rats | phytoestrogens | In vivo | middle  cerebral artery occlusion | Burguete et al. (37)/ 2006 |
|  |  |  |  | Daily weight | 5 weeks (3 to 8 weeks old) | Isoflavone-free chow (TD96155 diet) | Isoflavone-free Diet |  |  |  |  |  |
|  | | 🗸 |  | 96 h after I/R surgery | 10 days | Vehicle | 1 (Sham) | Laca mice | Naringin | In vivo | post-stroke  depression | Aggarwal et al. (38)/2010 |
|  |  |  |  | 96 h after I/R surgery | 10 days | Vehicle | 2 (I/R Control) |  |  |  |  |  |
|  |  |  |  | 96 h after I/R surgery | 10 days (7 days pre-surgery + 1 day pre-I/R) | 50 mg/kg Naringin | 3 (Naringin + I/R) |  |  |  |  |  |
|  |  |  |  | 96 h after I/R surgery | 10 days (7 days pre-surgery + 1 day pre-I/R) | 100 mg/kg Naringin | 4 (Naringin + I/R) |  |  |  |  |  |
|  |  |  |  | 96 h after I/R surgery | 10 days (7 days pre-surgery + 1 day pre-I/R) | 100 mg/kg L-Arginine + 5 mg/kg Sildenafil | 5 (L-Arginine + Sildenafil + I/R) |  |  |  |  |  |
|  |  |  |  | 96 h after I/R surgery | 10 days (7 days pre-surgery + 1 day pre-I/R) | 10 mg/kg L-NAME + 10 mg/kg 7-NI | 6 (L-NAME + 7-NI + I/R) |  |  |  |  |  |
|  |  |  |  | 96 h after I/R surgery | 10 days (7 days pre-surgery + 1 day pre-I/R) | 10 mg/kg L-NAME | 7 (L-NAME + I/R) |  |  |  |  |  |
|  |  |  |  | 96 h after I/R surgery | 10 days (7 days pre-surgery + 1 day pre-I/R) | 10 mg/kg 7-NI | 8 (7-NI + I/R) |  |  |  |  |  |
|  |  |  |  | 96 h after I/R surgery | 10 days (7 days pre-surgery + 1 day pre-I/R) | 50 mg/kg Naringin (1h before L-Arginine) + 100 mg/kg L-Arginine | 9 (Naringin (50 mg/kg) + L-Arginine (100 mg/kg) + I/R) |  |  |  |  |  |
|  |  |  |  | 96 h after I/R surgery | 10 days (7 days pre-surgery + 1 day pre-I/R) | 50 mg/kg Naringin (1h before Sildenafil) + 5 mg/kg Sildenafil | 10 (Naringin (50 mg/kg) + Sildenafil (5 mg/kg) + I/R) |  |  |  |  |  |
|  |  |  |  | 96 h after I/R surgery | 10 days (7 days pre-surgery + 1 day pre-I/R) | 50 mg/kg Naringin (1h before L-NAME/7-NI) + 10 mg/kg L-NAME + 10 mg/kg 7-NI | 11 (Naringin (50 mg/kg) + L-NAME (10 mg/kg) + 7-NI (10 mg/kg) + I/R) |  |  |  |  |  |
|  |  |  |  | 96 h after I/R surgery | 10 days (7 days pre-surgery + 1 day pre-I/R) | 50 mg/kg Naringin (1h before L-NAME) + 10 mg/kg L-NAME | 12 (Naringin (50 mg/kg) + L-NAME (10 mg/kg) + I/R) |  |  |  |  |  |
|  | | 🗸 |  | 24 h after reperfusion | Not Applicable | None | Control | Sprague–Dawley rats | anthocyanins | In vivo | cerebral ischemia | Shin et al. (39)/2006 |
|  |  |  |  | 24 h after reperfusion | Twice: 24 h and 30 min before MCAo | Medox-75 mg anthocyanin/capsule (300 mg/kg, p.o.) | Treatment |  |  |  |  |  |
| 🗸 | |  |  | 12 h after OGD (Cell viability assay) |  | None | Control | SH-SY5Y cells | Anthocyanin | In vitro | Hypoxia and ischemia | Cai et al. (40)/2020 |
|  |  |  |  | 12 h after OGD (Cell viability assay) | 12 h | Various concentrations (not specified) | Anthocyanin |  |  |  |  |  |
|  | | 🗸 |  | 24 hours after pMCAO (infarct volume) | 1 hour before pMCAO | Vehicle (control) | 1 (pre-treatment) | C57BL/6 mice | Anthocyanin | In vivo | Focal cerebral ischemia | Min et al. (41)/2011 |
|  |  |  |  | 24 hours after pMCAO (infarct volume) | 1 hour before pMCAO | CG (1 mg/kg) | 2 (pre-treatment) |  |  |  |  |  |
|  |  |  |  | 24 hours after pMCAO (infarct volume) | 1 hour before pMCAO | CG (2 mg/kg) | 3 (pre-treatment) |  |  |  |  |  |
|  |  |  |  | 24 hours after pMCAO (infarct volume) | 1 hour before pMCAO | CG (5 mg/kg) | 4 (pre-treatment) |  |  |  |  |  |
|  |  |  |  | 24 hours after pMCAO (infarct volume & neurological outcomes) | 3 hours after pMCAO (total 3 doses) | Vehicle (control) | 1 (delayed treatment) |  |  |  |  |  |
|  |  |  |  | 24 hours after pMCAO (infarct volume & neurological outcomes) | 3 hours after pMCAO (initial), then 2 additional doses 3h apart | CG (2 mg/kg x 3 doses) | 2 (delayed treatment) |  |  |  |  |  |
| 🗸 | |  |  | After sacrifice (brain and hippocampus) | - | Saline | Control | Wistar rats | thymoquinone | In vitro | transient forebrain  ischemia | Al-Majed et al. (42)/2006 |
|  |  |  |  | After sacrifice (brain and hippocampus) | 10 min ischemia + 7 days reperfusion | Sham surgery | Sham |  |  |  |  |  |
|  |  |  |  | After sacrifice (brain and hippocampus) | 10 min ischemia + 7 days reperfusion |  | Ischemia |  |  |  |  |  |
|  |  |  |  | After sacrifice (brain and hippocampus) | 5 days before ischemia + 7 days reperfusion | Thymoquinone (5 mg/kg/day p.o.) | Thymoquinone |  |  |  |  |  |
|  |  |  |  | After sacrifice (brain and hippocampus) |  | Thymoquinone (5 mg/kg/day p.o.) | Ischemia+Thymoquinone |  |  |  |  |  |
| 🗸 | |  |  | Body weight: before surgery, day 7, day 14; Blood pressure: day of surgery, day 14 | 14 days | None | Sham-Operated Control | Wistar rats | Candesartan | In vivo and vitro | chronic  cerebral hypoperfusion | Ozacmak et al. (43)/ 2007 |
|  |  |  |  | Body weight: before surgery, day 7, day 14; Blood pressure: day of surgery, day 14; Brain tissue MDA, GSH, AA levels: day 14 | 14 days | Vehicle | Ischemic Control |  |  |  |  |  |
|  |  |  |  | Body weight: before surgery, day 7, day 14; Blood pressure: day of surgery, day 14; Brain tissue MDA, GSH, AA levels: day 14 | 13 days (started 1 day after surgery) | Candesartan (0.5 mg/kg/day) | Candesartan Treatment |  |  |  |  |  |
|  | | 🗸 |  | NR | NR | Saline (0.9% NS) | Sham | Sprague-Dawley rats | Ellagic Acid | In vivo and in vitro | Cerebral Ischemia/  Reperfusion Injury | Wang et al. (44)/2019 |
|  |  |  |  | NR | NR | Ischemia-Reperfusion (surgical procedure) | I/R |  |  |  |  |  |
|  |  |  |  | NR | NR | Nimodipine (dose not mentioned) | Nimodipine |  |  |  |  |  |
|  |  |  |  | 2 hours after administration | NR | Shanhu Pill (oral) | Shanhu Pill |  |  |  |  |  |
|  |  |  |  | 2 hours after administration | NR | Ruyi Zhenbao Pill (oral) | Ruyi Zhenbao Pill |  |  |  |  |  |
|  |  |  |  | 2 hours after administration | NR | Chenxiang Pill (oral) | Chenxiang Pill |  |  |  |  |  |
|  |  |  |  | NR | NR | Extract A (10 mg/kg) | EA (low) |  |  |  |  |  |
|  |  |  |  | NR | NR | Extract A (30 mg/kg) | EA (medium) |  |  |  |  |  |
|  |  |  |  | NR | NR | Extract A (50 mg/kg) | EA (high) |  |  |  |  |  |
| 🗸 | |  |  | 1 hour after ischemia | 15 days | Ischemia (5 min) + Drinking water (15 days) | IR | Mongolian  gerbils | Crataegus flavonoids | In vivo | Brain Ischemia | Zhang et al. (45)/2004 |
|  |  |  |  | 1 hour after sham ischemia | 15 days | Sham Ischemia (no occlusion) + Drinking water (15 days) | Sham |  |  |  |  |  |
|  |  |  |  | 1 hour after ischemia | 15 days | Ischemia (5 min) + CF (0.5 mg/mL) in drinking water (15 days) | Low-Dose CF |  |  |  |  |  |
|  |  |  |  | 1 hour after ischemia | 15 days | Ischemia (5 min) + CF (2.5 mg/mL) in drinking water (15 days) | High-Dose CF |  |  |  |  |  |
| 🗸 | |  |  | Blood samples every 10 min before, during, and after 30 min infusion |  | Normal Saline | Control | GFAP-positive rat C6 glioma cells | Pyruvate | In vitro | Brain Ischemia-Reperfusion Injury | Ryou et al. (46)/2013 |
|  |  |  |  | Blood samples every 10 min before, during, and after 30 min infusion; Brain harvest and analysis at 24h reperfusion | 90 min | Sodium pyruvate (1M) | Pyruvate |  |  |  |  |  |
|  | | 🗸 |  | NR | 40 min (initial) + additional doses | Xylazine (8 mg/kg) + Ketamine (100 mg/kg) | 1 (Anesthesia) | ICR-CD1 mice | lactate | In vivo and in vitro | Cerebral ischemia | Berthet et al. (47)/2009 |
|  |  |  |  | NR | Throughout surgery | Isoflurane (1.5-2%) in O2/N2O | 2 (Anesthesia) |  |  |  |  |  |
|  |  |  |  | 48 h post-ischemia | NR | L-Lactate (100 mmol/L, 2 mL) or Vehicle (PBS) | 3 (Lactate) |  |  |  |  |  |
|  |  |  |  | 48 h post-ischemia | NR | L-Lactate (100 mmol/L, 2 mL) | 3a (Lactate Timing) |  |  |  |  |  |
|  |  |  |  | 48 h post-ischemia | NR | L-Lactate (100 mmol/L, 2 mL) | 3b (Lactate Timing) |  |  |  |  |  |
|  |  |  |  | Before and after injection | NR | L-Lactate (100 mmol/L, 2 mL) or PBS | 4 (MR Imaging) |  |  |  |  |  |
| 🗸 | | 🗸 |  | 2 h hypoxia + 2 h reoxygenation | 24 h pre-treatment | GA (0.1, 1, 10 μM) | SH-SY5Y cells | SH-SY5Y cells and Sprague-Dawley rats | gallic acid | In vivo and in vitro | Cerebral ischemia | Sun et al. (48)/ 2014 |
|  |  |  |  | 2 h hypoxia + 2 h reoxygenation | None | Control |  |  |  |  |  |  |
|  |  |  |  |  | None | - Sham | Experiment I: infarct volume |  |  |  |  |  |
|  |  |  |  | 20 min pre-ischemia | MCAO surgery | - MCAO |  |  |  |  |  |  |
|  |  |  |  | 20 min pre-ischemia | GA (50 mg/kg) | - GA (50 mg/kg) |  |  |  |  |  |  |
|  |  |  |  | 20 min pre-ischemia | GA (25 mg/kg) | - GA (25 mg/kg) |  |  |  |  |  |  |
|  |  |  |  | 20 min pre-ischemia | CsA (10 mg/kg) | - CsA (10 mg/kg) |  |  |  |  |  |  |
|  |  |  |  | 20 min pre-ischemia | Same as Experiment I | Same as Experiment I | Experiment II: Anti-apoptotic effect |  |  |  |  |  |
|  | | 🗸 |  | 4mg/mL Ascorbic Acid | | | | 217 patients | Ascorbic Acid | Clinical Trial | Vasospasm after  Aneurysmal Subarachnoid  Hemorrhage | Kodama et al. (49, 50)/ 2000 |
|  | | 🗸 |  | 4mg/mL Ascorbic Acid for 10-12 days post-operatively | | | | 63 patients | Ascorbic Acid | Clinical Trial | Symptomatic vasospasm | Satoh et al. (51)/ 2019 |
|  | |  | 🗸 | Baseline, 2 h post-drink | Single dose | 70.5 mg flavanoids (from juice) | High Flavanone | 24 patients | flavonoid | Clinical Trial |  | Lamport et al. (52)/ 2017 |
|  |  |  |  | Baseline, 2 h post-drink | Single dose | 0 mg flavanoids (cordial drink) | Control |  |  |  |  |  |
|  | |  | 🗸 | Before and after 12 weeks of supplementation | 12 weeks | 30 ml Blueberry concentrate (387 mg anthocyanidins) | Blueberry | 26 patients | blueberry concentrate supplementation | Clinical Trial |  | Bowtell et al. (53)/2017 |
|  |  |  |  | Before and after 12 weeks of supplementation | 12 weeks | 30 ml Blackcurrant and apple cordial (isocaloric) | Placebo |  |  |  |  |  |
| 🗸 | | 🗸 |  | Neurological deficit scores (24h, weekly) | 28 days (21 days pre + 14 days post MCAO) | Vehicle | I (Normal diet + vehicle) | Wistar rats | phytosome containing the combined extract of mulberry fruit and ginger | In vivo | Cerebral Ischemia | Palachai et al. (54)/2020 |
|  |  |  |  | Neurological deficit scores (24h, weekly), Brain infarction volume, Brain edema, C-reactive protein (24h post MCAO) | 28 days (21 days pre + 14 days post MCAO) | Vehicle | II (HCHF + Sham + vehicle) |  |  |  |  |  |
|  |  |  |  | Neurological deficit scores (24h, weekly), Brain infarction volume, Brain edema, C-reactive protein (24h post MCAO), Oxidative stress markers, DNMT-1, PPARγ, NF-κB, TNFα (end of study) | 28 days (21 days pre + 14 days post MCAO) | Vehicle | III (HCHF + MCAO + vehicle) |  |  |  |  |  |
|  |  |  |  | Neurological deficit scores (24h, weekly), Brain infarction volume, Brain edema, C-reactive protein (24h post MCAO), Oxidative stress markers, DNMT-1, PPARγ, NF-κB, TNFα (end of study) | 28 days (21 days pre + 14 days post MCAO) | 250 mg/kg BW | IV (HCHF + MCAO + vitamin C) |  |  |  |  |  |
|  |  |  |  | Neurological deficit scores (24h, weekly), Brain infarction volume, Brain edema, C-reactive protein (24h post MCAO), Oxidative stress markers, DNMT-1, PPARγ, NF-κB, TNFα (end of study) | 28 days (21 days pre + 14 days post MCAO) | 250 mg/kg BW | V (HCHF + MCAO + Piracetam) |  |  |  |  |  |
|  |  |  |  | Neurological deficit scores (24h, weekly), Brain infarction volume, Brain edema, C-reactive protein (24h post MCAO), Oxidative stress markers, DNMT-1, PPARγ, NF-κB, TNFα (end of study) | 28 days (21 days pre + 14 days post MCAO) | 50 mg/kg BW | VI (HCHF + MCAO + PMG) |  |  |  |  |  |
|  |  |  |  | Neurological deficit scores (24h, weekly), Brain infarction volume, Brain edema, C-reactive protein (24h post MCAO), Oxidative stress markers, DNMT-1, PPARγ, NF-κB, TNFα (end of study) | 28 days (21 days pre + 14 days post MCAO) | 100 mg/kg BW | VII (HCHF + MCAO + PMG) |  |  |  |  |  |
|  |  |  |  | Neurological deficit scores (24h, weekly), Brain infarction volume, Brain edema, C-reactive protein (24h post MCAO), Oxidative stress markers, DNMT-1, PPARγ, NF-κB, TNFα (end of study) | 28 days (21 days pre + 14 days post MCAO) | 200 mg/kg BW | VIII (HCHF + MCAO + PMG) |  |  |  |  |  |
|  | | 🗸 |  | 2 days after ischemia (neurological exam, neuronal damage assessment) | 2-3 h before ischemia | Ascorbic acid niosomal formulation (8 mg/kg) | 1 | Wistar rats | Niosomes of Ascorbic Acid and α-Tocopherol | In vivo and in vitro | Cerebral Ischemia | Varshosaz et al. (55)/2014 |
|  |  |  |  | 2 days after ischemia (neurological exam, neuronal damage assessment) | 2-3 h before ischemia | α-Tocopherol niosomal formulation (8 mg/kg) | 2 |  |  |  |  |  |
|  |  |  |  | 2 days after ischemia (neurological exam, neuronal damage assessment) | 2-3 h before ischemia | Free ascorbic acid (8 mg/kg) | 3 |  |  |  |  |  |
|  |  |  |  | 2 days after ischemia (neurological exam, neuronal damage assessment) | 2-3 h before ischemia | Normal saline | 4 |  |  |  |  |  |
|  |  |  |  | 2 days after ischemia (neurological exam, neuronal damage assessment) | 2-3 h before ischemia | Blank niosomes | 5 |  |  |  |  |  |
|  |  |  |  | 2 days after ischemia (neurological exam, neuronal damage assessment) | 2-3 h before ischemia | α-Tocopherol + Ascorbic acid niosomes (1:1, 8 mg/kg total) | 6 |  |  |  |  |  |
|  |  |  |  | 2 days after sham surgery (assumed normal histology) | NR | Sham-operated | 7 |  |  |  |  |  |
|  | | 🗸 |  | NR | NR | Vehicle | I (Control) (MES & PTZ) | wistar rats | Anisomeles malabarica | In vivo | epilepsy | Choudhary et al. (56)/2011 |
|  |  |  |  | NR | 45 min | Phenytoin (25 mg/kg, i.p.) | II (MES & PTZ) |  |  |  |  |  |
|  |  |  |  | NR | 45 min | Ethylacetate extract (400 mg/kg, i.p.) | III (MES & PTZ) |  |  |  |  |  |
|  |  |  |  | NR | 45 min | AMFF (12.5 mg/kg, i.p.) | IV (MES & PTZ) |  |  |  |  |  |
|  |  |  |  | NR | 45 min | AMFF (25 mg/kg, i.p.) | V (MES & PTZ) |  |  |  |  |  |
|  |  |  |  | NR | 45 min | AMFF (50 mg/kg, i.p.) | VI (MES & PTZ) |  |  |  |  |  |
|  |  |  |  | NR | NR | AMFF (6.25 mg/kg, i.p.) - 1 week pre-treatment | VII (MES & PTZ) |  |  |  |  |  |
|  |  |  |  | NR | NR | AMFF (12.5 mg/kg, i.p.) - 1 week pre-treatment | VIII (MES & PTZ) |  |  |  |  |  |
|  |  |  |  | NR | 45 min | AMTF (25 mg/kg, i.p.) | IX (MES & PTZ) |  |  |  |  |  |
|  |  |  |  | NR | 45 min | AMTF (50 mg/kg, i.p.) | X (MES & PTZ) |  |  |  |  |  |
|  |  |  |  | NR | NR | PTZ (50 mg/kg, i.p.) | PTZ control (MES) |  |  |  |  |  |
|  |  |  |  | NR | 45 min | DZP (2 mg/kg, i.p.) | DZP (PTZ control) |  |  |  |  |  |
|  | | 🗸 |  | 30 min after PTZ injection | 30 min | Tween 80-saline solution (10%) | Control 1 | Swiss albinomice | Galium spurium | In vivo and in vitro | Epilepsy | Orhan et al. (57)/2012 |
|  |  |  |  | 30 min after PTZ injection | 30 min (Ethosuximide) + 30 min (PTZ) | Ethosuximide (150 mg/kg) + PTZ (110 mg/kg) | Control 2 (Positive) |  |  |  |  |  |
|  |  |  |  | 30 min after PTZ injection (onset, | 30 min (Extract) + 30 min (PTZ) | Extract (250 mg/kg) + PTZ (110 mg/kg) | Test 1 |  |  |  |  |  |
|  |  |  |  | 30 min after PTZ injection | 30 min (Extract) + 30 min (PTZ) | Extract (500 mg/kg) + PTZ (110 mg/kg) | Test 2 |  |  |  |  |  |
|  |  |  |  | 30 min after PTZ injection | 30 min (Extract) + 30 min (PTZ) | Extract (1000 mg/kg) + PTZ (110 mg/kg) | Test 3 |  |  |  |  |  |
|  | | 🗸 |  | 30 minutes after PTZ injection (latency time, time to death, mortality rate, clonic convulsions rate) | 30 minutes before PTZ injection | Abelmoschus manihot ethanol extract (0.2 ml/20 g body weight) |  | ICR mice | Abelmoschus manihot ethanol  extract | In vivo | Epilepsy | Guo et al. (58)/2011 |
|  | | 🗸 |  | NR | | | | Cerebral cortex of rat brain | ethanol extract of T. parthenium | In vitro | Epilepsy | Jäger et al. (59)/2009 |
|  | | 🗸 |  | NR | | | | Wistar rats | Vitexin | In vitro | Pentylenetetrazole-Induced Seizure | Abbasi et al. (60)/2019 |
|  | | 🗸 |  | NR | | | | Sprague–Dawley rats | Hispidulin | In vitro |  | Lin et al. (61)/2012 |
| 🗸 | |  |  | Blood & Liver at sacrifice | 6 weeks (7 days acclimation + 42 days experiment) | Basic Diet & Water | Normal Control | Sprague–Dawley rats | Flavonoid-rich extract of Polygonum capitatum | In vivo | Atherosclerosis | Wang et al. (62)/2018 |
|  |  |  |  | Blood & Liver at sacrifice | 6 weeks (7 days acclimation + 42 days experiment) | High-Fat Diet & Water | HFD |  |  |  |  |  |
|  |  |  |  | Blood & Liver at sacrifice | 6 weeks (7 days acclimation + 42 days experiment) | High-Fat Diet + Xuezhikang (30 mg/kg) & Water | XZK |  |  |  |  |  |
|  |  |  |  | Blood & Liver at sacrifice | 6 weeks (7 days acclimation + 42 days experiment) | High-Fat Diet + High-Dose FPC (180 mg/kg) & Water | HFPC |  |  |  |  |  |
|  |  |  |  | Blood & Liver at sacrifice | 6 weeks (7 days acclimation + 42 days experiment) | High-Fat Diet + Medium-Dose FPC (90 mg/kg) & Water | MFPC |  |  |  |  |  |
|  |  |  |  | Blood & Liver at sacrifice | 6 weeks (7 days acclimation + 42 days experiment) | High-Fat Diet + Low-Dose FPC (45 mg/kg) & Water | LFPC |  |  |  |  |  |
| 🗸 | |  |  | NR | | | | Wistar rats | Diquertin and  Ascorbic Acid | In vivo and in vitro | brain ischemia | Plotnikov et al. (63)/ 2003 |
|  | |  | 🗸 | Latency to enter dark compartment during retention trial (24h after acquisition) | 1 hour before acquisition trial | 10% Tween 80 vehicle (p.o.) | Control | ICR  mice | stigmasterol | In vivo | scopolamine-induced memory  impairments | Park et al. (64)/ 2012 |
|  |  |  |  | Latency to enter dark compartment during retention trial (24h after acquisition) | 1 hour before acquisition trial | 2.5, 5, 10, or 20 mg/kg (p.o.) | Stigmasterol |  |  |  |  |  |
|  |  |  |  | Latency to enter dark compartment during retention trial (24h after acquisition) | 1 hour before acquisition trial | 10 mg/kg (p.o.) | Tacrine |  |  |  |  |  |
|  |  |  |  | Latency to enter dark compartment during acquisition trial | 30 min after Stigmasterol/Tacrine or saline | 1 mg/kg (i.p.) | Scopolamine |  |  |  |  |  |
|  |  |  |  | Latency to enter dark compartment during acquisition trial | 30 min after saline | 0.9% saline (i.p.) | Saline (Scopolamine Control) |  |  |  |  |  |
| 🗸 | |  | 🗸 | NR | 21 days | Vehicle (oral & i.p.) | 1 (Control) | Wistar rats | Curcumin | In vivo | phenytoin-induced cognitive  impairment | Reeta et al. (65)/2009 |
|  | |  |  | NR | 21 days | Phenytoin (75 mg/kg, i.p.) | 2 |  |  |  |  |  |
|  |  |  |  | NR | 21 days | Phenytoin (75 mg/kg, i.p.) + Curcumin (100 mg/kg, oral) | 3 |  |  |  |  |  |
|  |  |  |  | NR | 21 days | Phenytoin (75 mg/kg, i.p.) + Curcumin (200 mg/kg, oral) | 4 |  |  |  |  |  |
|  |  |  |  | NR | 21 days | Phenytoin (75 mg/kg, i.p.) + Curcumin (300 mg/kg, oral) | 5 |  |  |  |  |  |
|  |  |  |  | NR | 21 days | Curcumin (300 mg/kg, oral) + Vehicle (i.p.) | 6 |  |  |  |  |  |
| 🗸 | |  |  | At the end of the experiment | NR | Saline (2 mL/kg) | 1 (Sham) | Wistar rats | gallic acid | In vivo | oxidative stress induced by 6-  hydroxydopamine | Mansouri et al. (66)/2013 |
|  |  |  |  | At the end of the experiment | NR | Saline (2 mL/kg) | 2 (PD) |  |  |  |  |  |
|  |  |  |  | At the end of the experiment | 10 consecutive days | Gallic Acid (50 mg/kg) | 3 |  |  |  |  |  |
|  | |  |  | At the end of the experiment | 10 consecutive days | Gallic Acid (100 mg/kg) | 4 |  |  |  |  |  |
|  |  |  |  | At the end of the experiment | 10 consecutive days | Gallic Acid (200 mg/kg) | 5 |  |  |  |  |  |
|  | |  | 🗸 | Scopolamine-induced memory decline | | | | Wistar rats | ellagic acid | In vivo | scopolamine- and diazepam-induced  cognitive impairments | Mansouri et al. (67)/2016 |
|  |  |  |  | 30 min after scopolamine | NR | Vehicle | 1 (Control) |  |  |  |  |  |
|  |  |  |  | 30 min after scopolamine | NR | Scopolamine (0.4 mg/kg) + Vehicle | 2 |  |  |  |  |  |
|  |  |  |  | 30 min after scopolamine | NR | Scopolamine (0.4 mg/kg) + EA (10 mg/kg) | 3 |  |  |  |  |  |
|  |  |  |  | 30 min after scopolamine | NR | Scopolamine (0.4 mg/kg) + EA (30 mg/kg) | 4 |  |  |  |  |  |
|  |  |  |  | 30 min after scopolamine | NR | Scopolamine (0.4 mg/kg) + EA (100 mg/kg) | 5 |  |  |  |  |  |
|  |  |  |  | 30 min after EA | NR | EA (100 mg/kg) alone | 6 |  |  |  |  |  |
|  |  |  |  | Diazepam-induced memory decline (acute) | | | |  |  |  |  |  |
|  | |  |  | 30 min after diazepam | NR | Vehicle | 1 (Control) |  |  |  |  |  |
|  |  |  |  | 30 min after diazepam | NR | Diazepam (1 mg/kg) + Vehicle | 2 |  |  |  |  |  |
|  |  |  |  | 30 min after diazepam | NR | Diazepam (1 mg/kg) + EA (10 mg/kg) | 3 |  |  |  |  |  |
|  |  |  |  | 30 min after diazepam | NR | Diazepam (1 mg/kg) + EA (30 mg/kg) | 4 |  |  |  |  |  |
|  |  |  |  | 30 min after diazepam | NR | Diazepam (1 mg/kg) + EA (100 mg/kg) | 5 |  |  |  |  |  |
|  |  |  |  | 30 min after EA | NR | EA (100 mg/kg) alone | 6 |  |  |  |  |  |
|  |  |  |  | Diazepam-induced memory decline (chronic) | | | |  |  |  |  |  |
|  |  |  |  | 30 min after diazepam on day 10 | 10 days + 30 min | Vehicle | 1 (Control) |  |  |  |  |  |
|  |  |  |  | 30 min after diazepam on day 10 | 10 days + 30 min | Diazepam (1 mg/kg) + Vehicle | 2 |  |  |  |  |  |
|  |  |  |  | 30 min after diazepam on day 10 | 10 days + 30 min | EA (30 mg/kg) + Diazepam (1 mg/kg) | 3 |  |  |  |  |  |
|  |  |  |  | 30 min after EA on day 10 | 10 days + 30 min | EA (30 mg/kg) alone | 4 |  |  |  |  |  |
| 🗸 | |  |  | Blood glucose after 1 week, tissues after 28 days | 28 days | None | Normal Control | Wistar Albino  rats | Ellagic acid | In vivo | Diabetes | Uzar et al. (68)/2012 |
|  |  |  |  | Blood glucose after 1 week, tissues after 28 days | 28 days | Ellagic Acid (12.5 mg/ml in water) | Ellagic Acid Control |  |  |  |  |  |
|  |  |  |  | Blood glucose after 1 week, tissues after 28 days | Single injection | Streptozotocin (STZ) (50 mg/kg body weight) | Diabetic Control |  |  |  |  |  |
|  |  |  |  | Blood glucose after 1 week (STZ), tissues after 28 days | 1 week STZ + 21 days Ellagic Acid | STZ (50 mg/kg body weight) + Ellagic Acid (50 mg/kg/day orally) | Ellagic Acid Treated Diabetic |  |  |  |  |  |
| 🗸 | |  |  | NR | NR | No treatment | 1. Control | Wistar rats | ellagic acid | In vivo | Brain ischemia | Rafieirad et al. (69)/ 2107 |
|  |  |  |  | NR | 14 days | No treatment (ischemic hypoperfusion) | 2. Ischemia |  |  |  |  |  |
|  |  |  |  | NR | 14 days | Ellagic Acid (50 mg/kg) | 3. Ischemia + Ellagic Acid |  |  |  |  |  |
| 🗸 | |  |  | NR | 7 days before TBI | None | Control | Wistar rats | ellagic acid | In vivo | traumatic brain injury | Farbood et al. (70)/2015 |
|  |  |  |  | NR | 7 days before TBI | EA vehicle (10% DMSO in saline, 10 ml/kg) | Sham-injury |  |  |  |  |  |
|  |  |  |  | NR | 7 days before TBI + TBI | EA vehicle (10% DMSO in saline, 10 ml/kg) | Veh+TBI |  |  |  |  |  |
|  |  |  |  | NR | 7 days before TBI + TBI | EA (100 mg/kg) + 10% DMSO in saline (solvent, 10 ml/kg) | EA+TBI |  |  |  |  |  |
|  | | 🗸 |  | Behavioral testing (from 11.5 months) | 6 months (from 6 months to 11.5 months of age) | Sugar water (mimicking 1:40 PJ sugar content) | APPsw Control | transgenic mice | Pomegranate juice | In vivo | AD | Hartman et al. (71)./2016 |
|  |  |  |  | Behavioral testing (from 11.5 months) | 6 months (from 6 months of age) | PJ diluted 1:40 or 1:80 in filtered water (equivalent to 0.3–0.6mg polyphenols/day) | APPsw PJ |  |  |  |  |  |
|  |  |  |  | Behavioral testing (after 3 weeks of treatment) | 3-5 months to behavioral testing | Sugar water | Wildtype Control |  |  |  |  |  |
|  |  |  |  | Behavioral testing (after 3 weeks of treatment) | 3-5 months to behavioral testing | PJ diluted 1:40 in filtered water | Wildtype PJ |  |  |  |  |  |
|  | | 🗸 |  | NR | 2, 6, 12, and 24 hours | Ab42 (10 µM) | Ab42 Control | SH-SY5Y neuroblastoma cells | Ellagic acid | In vitro | Ab42-induced neurotoxicity | Feng et al. (72)./2009 |
|  |  |  |  | NR | 2, 6, 12, and 24 hours | Ab42 (10 µM) + EA (100 µM) | Ab42 + EA |  |  |  |  |  |
|  |  |  |  | NR | 24 hours | Control medium | Cells |  |  |  |  |  |
|  |  |  |  | NR | 48 hours | Ab42 (1 µM) | Cells + Ab42 |  |  |  |  |  |
|  |  |  |  | NR | 48 hours | Ab42 (1 µM) pre-incubated with EA | Cells + Ab42 + EA |  |  |  |  |  |
| 🗸 | |  |  | 24 h after differentiation (following treatment) | NR | None | Control | PC12 cells | Ellagic acid | In vitro | Oxidative Stress | Pavlica et al. (73) /2005 |
|  |  |  |  | 24 h after treatment | NR | NR | Peroxide or Iron |  |  |  |  |  |
|  |  |  |  | 24 h after treatment | NR | CGA & EA or metabolites (6.2, 12.5 & 25 mM) | Polyphenol + Peroxide/Iron (a) |  |  |  |  |  |
|  |  |  |  | 24 h after treatment | NR | CGA & EA or metabolites (6.2, 12.5 & 25 mM) | Polyphenol + Peroxide/Iron (b) |  |  |  |  |  |
| 🗸 | |  |  | After decapitation | 7 days | (30 mg/kg/day) | High Baicalein | Sprague-Dawley rats | Baicalein | In vivo | Parkinson’s disease | Hung et al. (74)/2016 |
|  |  |  |  | After decapitation | 7 days | (10 mg/kg/day) | Low Baicalein |  |  |  |  |  |
|  |  |  |  | After decapitation | 7 days | (10% DMSO in saline) | Vehicle |  |  |  |  |  |
|  | | 🗸 |  | - Before baicalein/vehicle pretreatment  - 1 day after last MPTP/saline administration (motor activity, pole test)  - 3 days after last behavioral assessment (striatal dopamine, metabolites, protein assay) | 1 week (daily) | Vehicle (0.9% NaCl) | Control (A) | C57BL/6 mice | Baicalein | In vivo | Parkinson’s disease | Cheng et al. (75)/2008 |
|  |  |  |  | - Before baicalein/vehicle pretreatment  - 1 day after last MPTP/saline administration (motor activity, pole test)  - 3 days after last behavioral assessment (striatal dopamine, metabolites, protein assay) | 4 days | MPTP (30 mg/kg/day, i.p.) for 4 consecutive days (total 120 mg/kg) | MPTP (B) |  |  |  |  |  |
|  |  |  |  | - Before baicalein/vehicle pretreatment  - 1 day after last MPTP/saline administration (motor activity, pole test)  - 3 days after last behavioral assessment (striatal dopamine, metabolites, protein assay) | 1 week (baicalein) + 4 days (MPTP) | Baicalein (200 mg/kg; i.g.) daily for 1 week before MPTP challenge, then MPTP (30 mg/kg/day, i.p.) for 4 consecutive days (total 120 mg/kg) | Baicalein + MPTP (C) |  |  |  |  |  |
|  | | 🗸 |  | In vivo | | | | Sprague-Dawley rats and Human neuroblastoma SH-SY5Y cells | baicalein | In vivo and in vitro | Parkinson’s disease | Zhang et al. (76)/ 2019 |
|  |  |  |  | Day 0, 14, 42 | 42 days | Sunflower oil (1 ml/kg/day) | Control |  |  |  |  |  |
|  |  |  |  | Day 0, 14, 42 | 42 days | Rotenone (2.5 mg/kg/day) | Rotenone |  |  |  |  |  |
|  |  |  |  | Day 14, 42 | 27 days (Baicalein) | Rotenone (2.5 mg/kg/day) + Baicalein (100 mg/kg/day) | Rotenone + Baicalein (100 mg/kg) |  |  |  |  |  |
|  |  |  |  | Day 14, 42 | 27 days (Baicalein) | Rotenone (2.5 mg/kg/day) + Baicalein (200 mg/kg/day) | Rotenone + Baicalein (200 mg/kg) |  |  |  |  |  |
|  |  |  |  | Day 14, 42 | 27 days (Baicalein) | Rotenone (2.5 mg/kg/day) + Baicalein (400 mg/kg/day) | Rotenone + Baicalein (400 mg/kg) |  |  |  |  |  |
|  |  |  |  | In vitro | | | |  |  |  |  |  |
|  |  |  |  | SH-SY5Y cells were exposed to different amounts of baicalein (0.1, 1, and 10 micromolar) and then incubated with either rotenone (1 micromolar) or a control solution for 24 hours. | | | |  |  |  |  |  |
| 🗸 | | 🗸 |  | NR | 14 days | Vehicle (2 µl) | Control (DMSO) | Sprague-Dawley rats | apigenin | In vivo | Parkinson's disease | Anusha et al. (77)/ 2017 |
|  | |  |  | NR | 14 days | ROT (6 µg/rat) | ROT |  |  |  |  |  |
|  |  |  |  | NR | 14 days | ROT (6 µg/rat) + AGN (10 mg/kg) | ROT + AGN (10 mg) |  |  |  |  |  |
|  |  |  |  | NR | 14 days | ROT (6 µg/rat) + AGN (20 mg/kg) | ROT + AGN (20 mg) |  |  |  |  |  |
|  |  |  |  | NR | 14 days | AGN (20 mg/kg) | AGN (20 mg) |  |  |  |  |  |
| 🗸 | | 🗸 |  | NR | 28 days | Vehicle | Vehicle/Vehicle | C57B/6J mice | chrysin | In vivo | Parkinson's disease | Goes et al. (78)/2018 |
|  |  |  |  | NR | 28 days | Chrysin (10 mg/kg, p.o.) | Vehicle/Chrysin |  |  |  |  |  |
|  |  |  |  | NR | 28 days | Vehicle | 6-OHDA/Vehicle |  |  |  |  |  |
|  |  |  |  | NR | 28 days | Chrysin (10 mg/kg, p.o.) | 6-OHDA/Chrysin |  |  |  |  |  |
| 🗸 | |  |  | NR | 6 days postinjection | PBS | Control (PBS) | Sprague-Dawley rats | Jeong | In vivo | Parkinson's disease | Jeong et al. (79)/ 2015 |
|  |  |  |  | NR | 6 days postinjection | MPP+ (7.4 lg in 2 μL) | MPP+ |  |  |  |  |  |
|  |  |  |  | NR | 1 day before + 6 days postinjection | Nobiletin (1 mg/kg/day) | Nobiletin (1 mg/kg) |  |  |  |  |  |
|  |  |  |  | NR | 1 day before + 6 days postinjection | Nobiletin (10 mg/kg/day) | Nobiletin (10 mg/kg) |  |  |  |  |  |
|  |  |  |  | NR | 1 day before + 6 days postinjection | Nobiletin (20 mg/kg/day) | Nobiletin (20 mg/kg) |  |  |  |  |  |
| 🗸 | | 🗸 |  | Day 13 | 12 days (Morin) | PBS | Normal control | C57BL/6 mice | Morin | In vivo and in vitro | Parkinson's disease | Lee et al. (80)/2016 |
|  |  |  |  | Day 13 | MPTP (single dose on day 13) | MPTP (20 mg/kg) | MPTP |  |  |  |  |  |
|  |  |  |  | Day 13 | MPTP (single dose on day 13) + Morin (12 days) | MPTP (20 mg/kg) + Morin (5 mg/kg) | MPTP + Morin (5 mg/kg) |  |  |  |  |  |
|  |  |  |  | Day 13 | MPTP (single dose on day 13) + Morin (12 days) | MPTP (20 mg/kg) + Morin (50 mg/kg) | MPTP + Morin (50 mg/kg) |  |  |  |  |  |
|  |  |  |  | Day 13 | Morin (12 days) | Morin (50 mg/kg) | Morin (50 mg/kg) |  |  |  |  |  |
| 🗸 | |  |  | NR | 14 days | Saline | Control | C57BL/6 mice | Quercetin | In vivo | Parkinson's disease | Lv et al. (81)/2007 |
|  |  |  |  | NR | 14 days | MPTP (30 mg/kg x 5) | MPTP |  |  |  |  |  |
|  |  |  |  | Day 14 | 14 days (Quercetin) + MPTP (5 days) | MPTP (30 mg/kg) + Quercetin (50 mg/kg/day) | Low dose (50 mg/kg) |  |  |  |  |  |
|  |  |  |  | Day 14 | 14 days (Quercetin) + MPTP (5 days) | MPTP (30 mg/kg) + Quercetin (100 mg/kg/day) | Middle dose (100 mg/kg) |  |  |  |  |  |
|  |  |  |  | Day 14 | 14 days (Quercetin) + MPTP (5 days) | MPTP (30 mg/kg) + Quercetin (200 mg/kg/day) | High dose (200 mg/kg) |  |  |  |  |  |
| 🗸 | |  |  | NR | 6 weeks | 25 mg/kg quercetin or vehicle solution (5% DMSO in corn oil) | MitoPark | transgenic mice and C57BL/6 control mice | Quercetin | In vivo | Parkinson's disease | Ay et al. (79/2017 |
|  |  |  |  | NR | 8 weeks | Vehicle (50% propylene glycol) or QB3C comprising quercetin (175 mg/kg), vitamin B3 (7 mg/kg), vitamin C (175 mg/kg), and folic acid (140 µg/kg) | MitoPark |  |  |  |  |  |
|  |  |  |  | NR | 6 weeks | Vehicle solution (5% DMSO in corn oil) | C57BL/6 control |  |  |  |  |  |
|  |  |  |  | NR | 8 weeks | Vehicle (50% propylene glycol) | C57BL/6-LC |  |  |  |  |  |
| 🗸 | | 🗸 |  | NR | 3 weeks | Saline | Sham (S) | Wistar rats | Rutin | In vivo | Parkinson's disease | Khan et al. (82)/2011 |
|  |  |  |  | NR | 3 weeks | 6-OHDA (5 lg/ll) | Lesioned (L) |  |  |  |  |  |
|  |  |  |  | NR | 3 weeks + 6-OHDA | Rutin (25 mg/kg/day) | Rutin (R) + Lesion (L) |  |  |  |  |  |
|  |  |  |  | NR | 3 weeks | Rutin (25 mg/kg/day) | Rutin (R) + Sham (S) |  |  |  |  |  |
| 🗸 | | 🗸 |  | NR | 4 days | Drug vehicle (10% Cremophor, p.o) | Control | Sprague-Dawley rats | curcumin and naringenin | In vivo | Parkinson's disease | Zbarsky et al. (83)/2005 |
|  |  |  |  | NR | 4 days | Curcumin (50 mg/kg, p.o) | Curcumin |  |  |  |  |  |
|  |  |  |  | NR | 4 days | Naringenin (50 mg/kg, p.o) | Naringenin |  |  |  |  |  |
| 🗸 | |  |  | 48 h after first MPTP injection | 5 days | 0.5% CMC + Saline | Control (CMC + Saline) | C57BL/6J mice | naringenin | In vivo | Parkinson's disease | Mani et al. (84)/ 2018 |
|  |  |  |  | 48 h after first MPTP injection | 5 days | 0.5% CMC + MPTP (80 mg/kg) | MPTP |  |  |  |  |  |
|  |  |  |  | 48 h after first MPTP injection | 5 days | NGN (25 mg/kg) + MPTP (80 mg/kg) | NGN (25 mg/kg) + MPTP |  |  |  |  |  |
|  |  |  |  | 48 h after first MPTP injection | 5 days | NGN (50 mg/kg) + MPTP (80 mg/kg) | NGN (50 mg/kg) + MPTP |  |  |  |  |  |
|  |  |  |  | 48 h after first MPTP injection | 5 days | NGN (100 mg/kg) + MPTP (80 mg/kg) | NGN (100 mg/kg) + MPTP |  |  |  |  |  |
|  |  |  |  | 48 h after first MPTP injection | 5 days | L-DOPA (100 mg/kg) + MPTP (80 mg/kg) | L-DOPA (100 mg/kg) + MPTP |  |  |  |  |  |
| 🗸 | |  |  | NR | NR | Saline | Sham | Wistar rats | Hesperetin | In vivo | Unilateral Striatal  6-Hydroxydopamine Damage | Kiasalari et al. (85)/2015 |
|  |  |  |  | NR | NR | Hesperetin (50 mg/kg/day) | Hesperetin-treated sham |  |  |  |  |  |
|  |  |  |  | NR | NR | 6-OHDA | Lesion (6-OHDA) |  |  |  |  |  |
|  |  |  |  | NR | NR | 6-OHDA + Cremophor | Lesion + Cremophor (vehicle) |  |  |  |  |  |
|  |  |  |  | NR | 1 week | 6-OHDA + Hesperetin (50 mg/kg/day) | Hesperetin-treated Lesion |  |  |  |  |  |
| 🗸 | |  |  | NR | 7-15 weeks | Naringin (80 mg/kg/day) |  | C57BL/6 mice | Naringin | In vivo | Parkinson's disease | Kim et al. (86)/ 2016 |
| 🗸 | |  |  | After behavioral testing | NR | Vehicle | Sham/Vehicle | C57BL/6 mice | hesperidin | In vivo | Parkinson's disease | Antunes et al. (87)/2014 |
|  |  |  |  | After behavioral testing | 7 days + 28 days | 6-OHDA | Sham/6-OHDA |  |  |  |  |  |
|  |  |  |  | After behavioral testing | 28 days | Hesperidin (50 mg/kg, p.o.) | Hesperidin/Vehicle |  |  |  |  |  |
|  |  |  |  | After behavioral testing | 7 days + 28 days | Hesperidin (50 mg/kg, p.o.) + 6-OHDA | Hesperidin/6-OHDA |  |  |  |  |  |
|  | |  | 🗸 | NR | NR | NR | NR | Wistar rats | Flavonoids from Stems and Leaves of Scutellaria baicalensis Georgi | In vivo | AD | Shengkai et al. (88)/ 2022 |
|  | | 🗸 |  | 70 days of age | once a day from 42 days after birth | 10 mg/kg body weight/day | EGCG-treated | Transgenic mice | (-)-Epigallocatechin-3-gallate | In vivo | ALS | Xu et al. (89)/ 2006 |
|  |  |  |  | 70 days of age | NR | vehicle | Vehicle-treated (control) |  |  |  |  |  |
| 🗸 | |  |  | 5 μM-40 μM | | | | C57BL/6, Wild Type (WT) and BALB/c mice and RAW 264.7 whole cells | Quercetin | In vivo and in vitro |  | Qureshi et al. (90)/ 2017 |
| 🗸 | |  |  | 48 h | 72 h | None | Control | Neuronal N2a Cells | Resveratrol, Quercetin, Apigenin | In vitro | Ketocholesterol-Induced Oxiapoptophagy | Yammine et al. (91)/ 2020 |
|  |  |  |  | 48 h | 72 h | 50 µM | 7-Ketocholesterol |  |  |  |  |  |
|  |  |  |  | 48 h | 72 h | 400 μM | α-Tocopherol |  |  |  |  |  |
|  |  |  |  | 48 h | 72 h | 1.5 - 25 µM | Resveratrol |  |  |  |  |  |
|  |  |  |  | 48 h | 72 h | 1.5 - 25 µM | Quercetin |  |  |  |  |  |
|  |  |  |  | 48 h | 72 h | 1.5 - 25 µM | Apigenin |  |  |  |  |  |
|  |  |  |  | 48 h | 72 h | 1.5 - 25 µM (2 h before 7KC) | Resveratrol (Pre-treatment) |  |  |  |  |  |
|  |  |  |  | 48 h | 72 h | 1.5 - 25 µM (2 h after 7KC) | Resveratrol (Post-treatment) |  |  |  |  |  |
|  |  |  |  | 48 h | 72 h | 1.5 - 25 µM (2 h before 7KC) | Quercetin (Pre-treatment) |  |  |  |  |  |
|  |  |  |  | 48 h | 72 h | 1.5 - 25 µM (2 h after 7KC) | Quercetin (Post-treatment) |  |  |  |  |  |
|  |  |  |  | 48 h | 72 h | 1.5 - 25 µM (2 h before 7KC) | Apigenin (Pre-treatment) |  |  |  |  |  |
|  |  |  |  | 48 h | 72 h | 1.5 - 25 µM (2 h after 7KC) | Apigenin (Post-treatment) |  |  |  |  |  |
|  |  |  |  | 48 h | 48 h | None | Control |  |  |  |  |  |
|  |  |  |  | 48 h | 48 h | 50 µM | 7-Ketocholesterol |  |  |  |  |  |
|  |  |  |  | 48 h | 48 h | 1.5 - 25 µM | Resveratrol |  |  |  |  |  |
|  |  |  |  | 48 h | 48 h | 1.5 - 25 µM | Quercetin |  |  |  |  |  |
|  |  |  |  | 48 h | 48 h | 1.5 - 25 µM | Apigenin |  |  |  |  |  |
| 🗸 | |  |  | Daily until all dead | NR | NR | Control | Caenorhabditis elegans | Epigallocatechin-3-gallate | In vivo |  | Xiong et al. (92)/ 2018 |
|  |  |  |  | Daily until all dead | * 2 days (or 6 days) for oxidative stress * Throughout lifespan for lifespan assay | NR | EGCG |  |  |  |  |  |
| 🗸 | |  |  | NR | | | | BY4742 yeast | Humulus japonicus extract | In vitro |  | Sung et al. (93)/ 2015 |
| 🗸 | |  |  | Weekly weight, Blood and liver at sacrifice | 8 weeks | Standard diet | Control | Wistar rats | Strawberry consumption | In vivo and in vitro |  | Giampieri et al. (94)/2017 |
|  |  |  |  | Weekly weight, Blood and liver at sacrifice | 8 weeks | Strawberry-enriched diet (15% of total calories from freeze-dried strawberry powder) | Strawberry-enriched diet |  |  |  |  |  |
| 🗸 | |  |  | NR | | | | Human foreskin fibroblasts BJ | quercetin | In vitro |  | Lewinska et al. (95)/ 2019 |
| Abbreviation: 7KC, 7-Ketocholesterol; AA, Ascorbic Acid; AD, Alzheimer's disease; AFF, flavonoids of A. esculentus flowers; AGN, Apigenin; ALS, Amyotrophic lateral sclerosis; AMFF, Anisomeles malabarica flavonoids fraction; AMTF, Anisomeles malabarica tannins fraction; Anth, Anthocyanins; BB, blueberry; BCCAO, Bilateral Common Carotid Arterial occlusion; CD, control diet; CF, Crataegus flavonoids; CG, cyanidin-3-O-glucoside; CGA, chlorogenic acid; CsA, cyclosporin A; D-gal, D-galactose; DHM, Dihydromyrcetin; DZP, diazepam; EA, ellagic acid; ED, elderberry diet; EGCG, (–)-epigallocatechin-3-gallate; EPM, Elevated plus maze test; FPC, flavonoids ingredient from Polygonum capitatum; GA, Gallic acid; GANP, Gallic Acid nanoparticles; HCHF, high-carbohydrate high-fat diet; HFD, High-Fat Diet; HFPC, high-fat diet plus high-dose FPC; I/R, ischemia/reperfusion; iAS, Arsenic induced- neurotoxicity; ICS, Icariside; KET, Ketamine; LFPC, low-fat diet plus high-dose FPC; MCAO, Middle Cerebral Artery Occlusion; ME, Mulberry extract; MFE, Mullberry fruit extract; MFPC, medium-fat diet plus high-dose FPC; MPP, 1-Methyl-4-phenylpyridinium; MPTP, 1-Methyl-4-phenyl-1,2,3,6-tetrahydropyridine; NGN, naringenin; NORT, Novel object recognition test; NP, nanoparticles; NR, not reported; OHDA, hydroxidopamine; PJ, Pomegranate Juice; PMG, phytosome containing the combined extract of mulberry fruit and ginger; PT, Piracetam; ROT, rotenone; SC, Scopolamine; SD, Sutherlandia diet; STZ, Streptozotocin; T1DM, Type 1 diabetes mellitus; XZK, Xuezhikang | | | | | | | | | | | | |

ADDIN EN.REFLIST 1. Chen Y, Li Q, Zou Y, Zhou ZX, Feng WW, Bao YT, et al. Protective effect of mulberry extract against Pb-induced learning and memory deficits in mice. Biomed Environ Sci. 2014;27(1):70-3.

2. Parle M, Dhingra D. Ascorbic Acid: a promising memory-enhancer in mice. J Pharmacol Sci. 2003;93(2):129-35.

3. Tan L, Yang H, Pang W, Li H, Liu W, Sun S, et al. Investigation on the Role of BDNF in the Benefits of Blueberry Extracts for the Improvement of Learning and Memory in Alzheimer's Disease Mouse Model. J Alzheimers Dis. 2017;56(2):629-40.

4. Kiasalari Z, Heydarifard R, Khalili M, Afshin-Majd S, Baluchnejadmojarad T, Zahedi E, et al. Ellagic acid ameliorates learning and memory deficits in a rat model of Alzheimer's disease: an exploration of underlying mechanisms. Psychopharmacology (Berl). 2017;234(12):1841-52.

5. Puerta E, Suárez-Santiago JE, Santos-Magalhães NS, Ramirez MJ, Irache JM. Effect of the oral administration of nanoencapsulated quercetin on a mouse model of Alzheimer’s disease. International journal of pharmaceutics. 2017;517(1-2):50-7.

6. Sabogal-Guaqueta AM, Munoz-Manco JI, Ramirez-Pineda JR, Lamprea-Rodriguez M, Osorio E, Cardona-Gomez GP. The flavonoid quercetin ameliorates Alzheimer's disease pathology and protects cognitive and emotional function in aged triple transgenic Alzheimer's disease model mice. Neuropharmacology. 2015;93:134-45.

7. Moghbelinejad S, Nassiri-Asl M, Farivar TN, Abbasi E, Sheikhi M, Taghiloo M, et al. Rutin activates the MAPK pathway and BDNF gene expression on beta-amyloid induced neurotoxicity in rats. Toxicol Lett. 2014;224(1):108-13.

8. Ali T, Kim MJ, Rehman SU, Ahmad A, Kim MO. Anthocyanin-loaded PEG-gold nanoparticles enhanced the neuroprotection of anthocyanins in an Aβ1–42 mouse model of Alzheimer’s disease. Molecular neurobiology. 2017;54(8):6490-506.

9. Kim MJ, Rehman SU, Amin FU, Kim MO. Enhanced neuroprotection of anthocyanin-loaded PEG-gold nanoparticles against Aβ1-42-induced neuroinflammation and neurodegeneration via the NF-KB/JNK/GSK3β signaling pathway. Nanomedicine: Nanotechnology, Biology and Medicine. 2017;13(8):2533-44.

10. Seong K-J, Lee H-G, Kook MS, Ko H-M, Jung J-Y, Kim W-J. Epigallocatechin-3-gallate rescues LPS-impaired adult hippocampal neurogenesis through suppressing the TLR4-NF-κB signaling pathway in mice. The Korean journal of physiology & pharmacology: official journal of the Korean Physiological Society and the Korean Society of Pharmacology. 2016;20(1):41.

11. Liu CM, Yang W, Ma JQ, Yang HX, Feng ZJ, Sun JM, et al. Dihydromyricetin Inhibits Lead-Induced Cognitive Impairments and Inflammation by the Adenosine 5'-Monophosphate-Activated Protein Kinase Pathway in Mice. J Agric Food Chem. 2018;66(30):7975-82.

12. Yang W, Tian ZK, Yang HX, Feng ZJ, Sun JM, Jiang H, et al. Fisetin improves lead-induced neuroinflammation, apoptosis and synaptic dysfunction in mice associated with the AMPK/SIRT1 and autophagy pathway. Food Chem Toxicol. 2019;134:110824.

13. Yin C, Deng Y, Liu Y, Gao J, Yan L, Gong Q. Icariside II Ameliorates Cognitive Impairments Induced by Chronic Cerebral Hypoperfusion by Inhibiting the Amyloidogenic Pathway: Involvement of BDNF/TrkB/CREB Signaling and Up-Regulation of PPARalpha and PPARgamma in Rats. Front Pharmacol. 2018;9:1211.

14. Li Y, Li J, Li S, Li Y, Wang X, Liu B, et al. Curcumin attenuates glutamate neurotoxicity in the hippocampus by suppression of ER stress-associated TXNIP/NLRP3 inflammasome activation in a manner dependent on AMPK. Toxicol Appl Pharmacol. 2015;286(1):53-63.

15. Valles SL, Dolz-Gaiton P, Gambini J, Borras C, LLoret A, Pallardo FV, et al. Estradiol or genistein prevent Alzheimer's disease-associated inflammation correlating with an increase PPARγ expression in cultured astrocytes. Brain research. 2010;1312:138-44.

16. Bonet-Costa V, Herranz-Perez V, Blanco-Gandia M, Mas-Bargues C, Ingles M, Garcia-Tarraga P, et al. Clearing amyloid-β through PPAR γ/ApoE activation by genistein is a treatment of experimental Alzheimer’s disease. Journal of Alzheimer's Disease. 2016;51(3):701-11.

17. Nagpal K, Singh SK, Mishra DN. Nanoparticle mediated brain targeted delivery of gallic acid: in vivo behavioral and biochemical studies for protection against scopolamine-induced amnesia. Drug Deliv. 2013;20(3-4):112-9.

18. Wang Q, Xu J, Rottinghaus GE, Simonyi A, Lubahn D, Sun GY, et al. Resveratrol protects against global cerebral ischemic injury in gerbils. Brain research. 2002;958(2):439-47.

19. Wang Q, Sun AY, Simonyi A, Jensen MD, Shelat PB, Rottinghaus GE, et al. Neuroprotective mechanisms of curcumin against cerebral ischemia‐induced neuronal apoptosis and behavioral deficits. Journal of neuroscience research. 2005;82(1):138-48.

20. Chuang DY, Cui J, Simonyi A, Engel VA, Chen S, Fritsche KL, et al. Dietary Sutherlandia and elderberry mitigate cerebral ischemia-induced neuronal damage and attenuate p47phox and phospho-ERK1/2 expression in microglial cells. ASN neuro. 2014;6(6):1759091414554946.

21. Moghaddam MH, Bayat A-H, Eskandari N, Abdollahifar M-a, Fotouhi F, Forouzannia A, et al. Elderberry diet ameliorates motor function and prevents oxidative stress-induced cell death in rat models of Huntington disease. Brain Research. 2021;1762:147444.

22. Sharma P, Kumar M, Bansal N. Ellagic acid prevents 3-nitropropionic acid induced symptoms of Huntington's disease. Naunyn Schmiedebergs Arch Pharmacol. 2021;394(9):1917-28.

23. Shin SK, Yoo J-M, Li FY, Baek SY, Kim MR. Mulberry fruit improves memory in scopolamine-treated mice: Role of cholinergic function, antioxidant system, and TrkB/Akt signaling. Nutritional neuroscience. 2021;24(12):940-50.

24. Andres-Lacueva C, Shukitt-Hale B, Galli RL, Jauregui O, Lamuela-Raventos RM, Joseph JA. Anthocyanins in aged blueberry-fed rats are found centrally and may enhance memory. Nutritional neuroscience. 2005;8(2):111-20.

25. Rehman SU, Shah SA, Ali T, Chung JI, Kim MO. Anthocyanins reversed D-galactose-induced oxidative stress and neuroinflammation mediated cognitive impairment in adult rats. Molecular neurobiology. 2017;54(1):255-71.

26. Wei J, Zhang G, Zhang X, Xu D, Gao J, Fan J, et al. Anthocyanins from black chokeberry (Aroniamelanocarpa Elliot) delayed aging-related degenerative changes of brain. Journal of agricultural and food chemistry. 2017;65(29):5973-84.

27. Carey AN, Fisher DR, Rimando AM, Gomes SM, Bielinski DF, Shukitt-Hale B. Stilbenes and anthocyanins reduce stress signaling in BV-2 mouse microglia. Journal of agricultural and food chemistry. 2013;61(25):5979-86.

28. Nam SM, Seo M, Seo JS, Rhim H, Nahm SS, Cho IH, et al. Ascorbic Acid Mitigates D-galactose-Induced Brain Aging by Increasing Hippocampal Neurogenesis and Improving Memory Function. Nutrients. 2019;11(1):176.

29. Bastianetto S, Yao ZX, Papadopoulos V, Quirion R. Neuroprotective effects of green and black teas and their catechin gallate esters against beta-amyloid-induced toxicity. Eur J Neurosci. 2006;23(1):55-64.

30. Salehi A, Rabiei Z, Setorki M. Effect of gallic acid on chronic restraint stress-induced anxiety and memory loss in male BALB/c mice. Iran J Basic Med Sci. 2018;21(12):1232-7.

31. Samad N, Jabeen S, Imran I, Zulfiqar I, Bilal K. Protective effect of gallic acid against arsenic-induced anxiety-/depression- like behaviors and memory impairment in male rats. Metab Brain Dis. 2019;34(4):1091-102.

32. Alfaris N, Alshammari G, Altamimi J, Aljabryn D, Alagal R, Aldera H, et al. Ellagic acid prevents streptozotocin-induced hippocampal damage and memory loss in rats by stimulating Nrf2 and nuclear factor-κB, and activating insulin receptor substrate/PI3K/Akt axis. Journal of Physiology and Pharmacology: an Official Journal of the Polish Physiological Society. 2021;72(4).

33. Brum GF, Rosa HZ, Rossato DR, Rosa JLO, Metz VG, Milanesi LH, et al. Binge and Subchronic Exposure to Ketamine Promote Memory Impairments and Damages in the Hippocampus and Peripheral Tissues in Rats: Gallic Acid Protective Effects. Neurotox Res. 2020;38(2):274-86.

34. Kumar RS, Narayanan SN, Nayak S. Ascorbic acid protects against restraint stress-induced memory deficits in Wistar rats. Clinics (Sao Paulo). 2009;64(12):1211-7.

35. Rivera F, Urbanavicius J, Gervaz E, Morquio A, Dajas F. Some aspects of thein vivo neuroprotective capacity of flavonoids: bioavailability and structure-activity relationship. Neurotoxicity research. 2004;6(7):543-53.

36. Luo Y, Cui HX, Jia A, Jia SS, Yuan K. The Protective Effect of the Total Flavonoids of Abelmoschus esculentus L. Flowers on Transient Cerebral Ischemia-Reperfusion Injury Is due to Activation of the Nrf2-ARE Pathway. Oxid Med Cell Longev. 2018;2018:8987173.

37. Burguete MC, Torregrosa Pérez‐Asensio G, Fernando J, Castelló‐Ruiz M, Salom Gil JB, José V, Alborch E. Dietary phytoestrogens improve stroke outcome after transient focal cerebral ischemia in rats. European Journal of Neuroscience. 2006;23(3):703-10.

38. Aggarwal A, Gaur V, Kumar A. Nitric oxide mechanism in the protective effect of naringin against post-stroke depression (PSD) in mice. Life sciences. 2010;86(25-26):928-35.

39. Shin WH, Park SJ, Kim EJ. Protective effect of anthocyanins in middle cerebral artery occlusion and reperfusion model of cerebral ischemia in rats. Life Sci. 2006;79(2):130-7.

40. Cai Y, Li X, Pan Z, Zhu Y, Tuo J, Meng Q, et al. Anthocyanin ameliorates hypoxia and ischemia induced inflammation and apoptosis by increasing autophagic flux in SH-SY5Y cells. Eur J Pharmacol. 2020;883:173360.

41. Min J, Yu SW, Baek SH, Nair KM, Bae ON, Bhatt A, et al. Neuroprotective effect of cyanidin-3-O-glucoside anthocyanin in mice with focal cerebral ischemia. Neurosci Lett. 2011;500(3):157-61.

42. Al-Majed AA, Al-Omar FA, Nagi MN. Neuroprotective effects of thymoquinone against transient forebrain ischemia in the rat hippocampus. Eur J Pharmacol. 2006;543(1-3):40-7.

43. Ozacmak VH, Sayan H, Cetin A, Akyildiz-Igdem A. AT1 receptor blocker candesartan-induced attenuation of brain injury of rats subjected to chronic cerebral hypoperfusion. Neurochem Res. 2007;32(8):1314-21.

44. Wang Y, Wu Y, Liang C, Tan R, Tan L, Tan R. Pharmacodynamic Effect of Ellagic Acid on Ameliorating Cerebral Ischemia/Reperfusion Injury. Pharmacology. 2019;104(5-6):320-31.

45. Zhang DL, Zhang YT, Yin JJ, Zhao BL. Oral administration of Crataegus flavonoids protects against ischemia/reperfusion brain damage in gerbils. Journal of neurochemistry. 2004;90(1):211-9.

46. Ryou MG, Liu R, Ren M, Sun J, Mallet RT, Yang SH. Pyruvate protects the brain against ischemia-reperfusion injury by activating the erythropoietin signaling pathway. Stroke. 2012;43(4):1101-7.

47. Berthet C, Lei H, Thevenet J, Gruetter R, Magistretti PJ, Hirt L. Neuroprotective role of lactate after cerebral ischemia. J Cereb Blood Flow Metab. 2009;29(11):1780-9.

48. Sun J, Li YZ, Ding YH, Wang J, Geng J, Yang H, et al. Neuroprotective effects of gallic acid against hypoxia/reoxygenation-induced mitochondrial dysfunctions in vitro and cerebral ischemia/reperfusion injury in vivo. Brain Res. 2014;1589:126-39.

49. Kodama N, Sasaki T, Kawakami M, Sato M, Asari J. Cisternal irrigation therapy with urokinase and ascorbic acid for prevention of vasospasm after aneurysmal subarachnoid hemorrhage. Outcome in 217 patients. Surg Neurol. 2000;53(2):110-7; discussion 7-8.

50. Kodama N, Matsumoto M, Sasaki T, Konno Y, Sato T. Cisternal irrigation therapy with urokinase and ascorbic acid for prevention of vasospasm. Cerebral Vasospasm: Springer; 2001. p. 171-4.

51. Satoh A, Sugiyama T, Ooigawa H, Nakajima H, Ogura T, Neki H, et al. Prevention of symptomatic vasospasm by continuous cisternal irrigation with mock-CSF containing ascorbic acid and Mg(2+). Acta Neurochir Suppl. 2010;107:115-8.

52. Lamport DJ, Pal D, Macready AL, Barbosa-Boucas S, Fletcher JM, Williams CM, et al. The effects of flavanone-rich citrus juice on cognitive function and cerebral blood flow: an acute, randomised, placebo-controlled cross-over trial in healthy, young adults. British journal of nutrition. 2016;116(12):2160-8.

53. Bowtell JL, Aboo-Bakkar Z, Conway ME, Adlam A-LR, Fulford J. Enhanced task-related brain activation and resting perfusion in healthy older adults after chronic blueberry supplementation. Applied Physiology, Nutrition, and Metabolism. 2017;42(7):773-9.

54. Palachai N, Wattanathorn J, Muchimapura S, Thukham-Mee W. Phytosome Loading the Combined Extract of Mulberry Fruit and Ginger Protects against Cerebral Ischemia in Metabolic Syndrome Rats. Oxid Med Cell Longev. 2020;2020:5305437.

55. Varshosaz J, Taymouri S, Pardakhty A, Asadi-Shekaari M, Babaee A. Niosomes of ascorbic acid and α-tocopherol in the cerebral ischemia-reperfusion model in male rats. (2314-6141 (Electronic)).

56. Choudhary N, Bijjem KR, Kalia AN. Antiepileptic potential of flavonoids fraction from the leaves of Anisomeles malabarica. J Ethnopharmacol. 2011;135(2):238-42.

57. Orhan N, Deliorman Orhan D, Aslan M, Sukuroglu M, Orhan IE. UPLC-TOF-MS analysis of Galium spurium towards its neuroprotective and anticonvulsant activities. J Ethnopharmacol. 2012;141(1):220-7.

58. Guo J, Xue C, Duan JA, Qian D, Tang Y, You Y. Anticonvulsant, antidepressant-like activity of Abelmoschus manihot ethanol extract and its potential active components in vivo. Phytomedicine. 2011;18(14):1250-4.

59. Jager AK, Krydsfeldt K, Rasmussen HB. Bioassay-guided isolation of apigenin with GABA-benzodiazepine activity from Tanacetum parthenium. Phytother Res. 2009;23(11):1642-4.

60. Abbasi E, Nassiri-Asl M, Shafeei M, Sheikhi M. Neuroprotective effects of vitexin, a flavonoid, on pentylenetetrazole-induced seizure in rats. Chem Biol Drug Des. 2012;80(2):274-8.

61. Lin TY, Lu CW, Wang CC, Lu JF, Wang SJ. Hispidulin inhibits the release of glutamate in rat cerebrocortical nerve terminals. Toxicol Appl Pharmacol. 2012;263(2):233-43.

62. Wang Z, Jiang X. Flavonoid-rich extract of Polygonum capitatum attenuates high-fat diet–induced atherosclerosis development and inflammatory and oxidative stress in hyperlipidemia rats. European Journal of Inflammation. 2018;16.

63. Plotnikov M, Aliev O, Maslov MJ, Vasiliev A, Tjukavkina N. Correction of the high blood viscosity syndrome by a mixture of diquertin and ascorbic acid in vitro and in vivo. Phytotherapy Research. 2003;17(3):276-8.

1. Chen Y, Li Q, Zou Y, Zhou ZX, Feng WW, Bao YT, et al. Protective effect of mulberry extract against Pb-induced learning and memory deficits in mice. Biomed Environ Sci. 2014;27(1):70-3.

2. Parle M, Dhingra D. Ascorbic Acid: a promising memory-enhancer in mice. J Pharmacol Sci. 2003;93(2):129-35.

3. Tan L, Yang H, Pang W, Li H, Liu W, Sun S, et al. Investigation on the Role of BDNF in the Benefits of Blueberry Extracts for the Improvement of Learning and Memory in Alzheimer's Disease Mouse Model. J Alzheimers Dis. 2017;56(2):629-40.

4. Kiasalari Z, Heydarifard R, Khalili M, Afshin-Majd S, Baluchnejadmojarad T, Zahedi E, et al. Ellagic acid ameliorates learning and memory deficits in a rat model of Alzheimer's disease: an exploration of underlying mechanisms. Psychopharmacology (Berl). 2017;234(12):1841-52.

5. Puerta E, Suárez-Santiago JE, Santos-Magalhães NS, Ramirez MJ, Irache JM. Effect of the oral administration of nanoencapsulated quercetin on a mouse model of Alzheimer’s disease. International journal of pharmaceutics. 2017;517(1-2):50-7.

6. Sabogal-Guaqueta AM, Munoz-Manco JI, Ramirez-Pineda JR, Lamprea-Rodriguez M, Osorio E, Cardona-Gomez GP. The flavonoid quercetin ameliorates Alzheimer's disease pathology and protects cognitive and emotional function in aged triple transgenic Alzheimer's disease model mice. Neuropharmacology. 2015;93:134-45.

7. Moghbelinejad S, Nassiri-Asl M, Farivar TN, Abbasi E, Sheikhi M, Taghiloo M, et al. Rutin activates the MAPK pathway and BDNF gene expression on beta-amyloid induced neurotoxicity in rats. Toxicol Lett. 2014;224(1):108-13.

8. Ali T, Kim MJ, Rehman SU, Ahmad A, Kim MO. Anthocyanin-loaded PEG-gold nanoparticles enhanced the neuroprotection of anthocyanins in an Aβ1–42 mouse model of Alzheimer’s disease. Molecular neurobiology. 2017;54(8):6490-506.

9. Kim MJ, Rehman SU, Amin FU, Kim MO. Enhanced neuroprotection of anthocyanin-loaded PEG-gold nanoparticles against Aβ1-42-induced neuroinflammation and neurodegeneration via the NF-KB/JNK/GSK3β signaling pathway. Nanomedicine: Nanotechnology, Biology and Medicine. 2017;13(8):2533-44.

10. Seong K-J, Lee H-G, Kook MS, Ko H-M, Jung J-Y, Kim W-J. Epigallocatechin-3-gallate rescues LPS-impaired adult hippocampal neurogenesis through suppressing the TLR4-NF-κB signaling pathway in mice. The Korean journal of physiology & pharmacology: official journal of the Korean Physiological Society and the Korean Society of Pharmacology. 2016;20(1):41.

11. Liu CM, Yang W, Ma JQ, Yang HX, Feng ZJ, Sun JM, et al. Dihydromyricetin Inhibits Lead-Induced Cognitive Impairments and Inflammation by the Adenosine 5'-Monophosphate-Activated Protein Kinase Pathway in Mice. J Agric Food Chem. 2018;66(30):7975-82.

12. Yang W, Tian ZK, Yang HX, Feng ZJ, Sun JM, Jiang H, et al. Fisetin improves lead-induced neuroinflammation, apoptosis and synaptic dysfunction in mice associated with the AMPK/SIRT1 and autophagy pathway. Food Chem Toxicol. 2019;134:110824.

13. Yin C, Deng Y, Liu Y, Gao J, Yan L, Gong Q. Icariside II Ameliorates Cognitive Impairments Induced by Chronic Cerebral Hypoperfusion by Inhibiting the Amyloidogenic Pathway: Involvement of BDNF/TrkB/CREB Signaling and Up-Regulation of PPARalpha and PPARgamma in Rats. Front Pharmacol. 2018;9:1211.

14. Li Y, Li J, Li S, Li Y, Wang X, Liu B, et al. Curcumin attenuates glutamate neurotoxicity in the hippocampus by suppression of ER stress-associated TXNIP/NLRP3 inflammasome activation in a manner dependent on AMPK. Toxicol Appl Pharmacol. 2015;286(1):53-63.

15. Valles SL, Dolz-Gaiton P, Gambini J, Borras C, LLoret A, Pallardo FV, et al. Estradiol or genistein prevent Alzheimer's disease-associated inflammation correlating with an increase PPARγ expression in cultured astrocytes. Brain research. 2010;1312:138-44.

16. Bonet-Costa V, Herranz-Perez V, Blanco-Gandia M, Mas-Bargues C, Ingles M, Garcia-Tarraga P, et al. Clearing amyloid-β through PPAR γ/ApoE activation by genistein is a treatment of experimental Alzheimer’s disease. Journal of Alzheimer's Disease. 2016;51(3):701-11.

17. Nagpal K, Singh SK, Mishra DN. Nanoparticle mediated brain targeted delivery of gallic acid: in vivo behavioral and biochemical studies for protection against scopolamine-induced amnesia. Drug Deliv. 2013;20(3-4):112-9.

18. Wang Q, Xu J, Rottinghaus GE, Simonyi A, Lubahn D, Sun GY, et al. Resveratrol protects against global cerebral ischemic injury in gerbils. Brain research. 2002;958(2):439-47.

19. Wang Q, Sun AY, Simonyi A, Jensen MD, Shelat PB, Rottinghaus GE, et al. Neuroprotective mechanisms of curcumin against cerebral ischemia‐induced neuronal apoptosis and behavioral deficits. Journal of neuroscience research. 2005;82(1):138-48.

20. Chuang DY, Cui J, Simonyi A, Engel VA, Chen S, Fritsche KL, et al. Dietary Sutherlandia and elderberry mitigate cerebral ischemia-induced neuronal damage and attenuate p47phox and phospho-ERK1/2 expression in microglial cells. ASN neuro. 2014;6(6):1759091414554946.

21. Moghaddam MH, Bayat A-H, Eskandari N, Abdollahifar M-a, Fotouhi F, Forouzannia A, et al. Elderberry diet ameliorates motor function and prevents oxidative stress-induced cell death in rat models of Huntington disease. Brain Research. 2021;1762:147444.

22. Sharma P, Kumar M, Bansal N. Ellagic acid prevents 3-nitropropionic acid induced symptoms of Huntington's disease. Naunyn Schmiedebergs Arch Pharmacol. 2021;394(9):1917-28.

23. Shin SK, Yoo J-M, Li FY, Baek SY, Kim MR. Mulberry fruit improves memory in scopolamine-treated mice: Role of cholinergic function, antioxidant system, and TrkB/Akt signaling. Nutritional neuroscience. 2021;24(12):940-50.

24. Andres-Lacueva C, Shukitt-Hale B, Galli RL, Jauregui O, Lamuela-Raventos RM, Joseph JA. Anthocyanins in aged blueberry-fed rats are found centrally and may enhance memory. Nutritional neuroscience. 2005;8(2):111-20.

25. Rehman SU, Shah SA, Ali T, Chung JI, Kim MO. Anthocyanins reversed D-galactose-induced oxidative stress and neuroinflammation mediated cognitive impairment in adult rats. Molecular neurobiology. 2017;54(1):255-71.

26. Wei J, Zhang G, Zhang X, Xu D, Gao J, Fan J, et al. Anthocyanins from black chokeberry (Aroniamelanocarpa Elliot) delayed aging-related degenerative changes of brain. Journal of agricultural and food chemistry. 2017;65(29):5973-84.

27. Carey AN, Fisher DR, Rimando AM, Gomes SM, Bielinski DF, Shukitt-Hale B. Stilbenes and anthocyanins reduce stress signaling in BV-2 mouse microglia. Journal of agricultural and food chemistry. 2013;61(25):5979-86.

28. Nam SM, Seo M, Seo JS, Rhim H, Nahm SS, Cho IH, et al. Ascorbic Acid Mitigates D-galactose-Induced Brain Aging by Increasing Hippocampal Neurogenesis and Improving Memory Function. Nutrients. 2019;11(1):176.

29. Bastianetto S, Yao ZX, Papadopoulos V, Quirion R. Neuroprotective effects of green and black teas and their catechin gallate esters against beta-amyloid-induced toxicity. Eur J Neurosci. 2006;23(1):55-64.

30. Salehi A, Rabiei Z, Setorki M. Effect of gallic acid on chronic restraint stress-induced anxiety and memory loss in male BALB/c mice. Iran J Basic Med Sci. 2018;21(12):1232-7.

31. Samad N, Jabeen S, Imran I, Zulfiqar I, Bilal K. Protective effect of gallic acid against arsenic-induced anxiety-/depression- like behaviors and memory impairment in male rats. Metab Brain Dis. 2019;34(4):1091-102.

32. Alfaris N, Alshammari G, Altamimi J, Aljabryn D, Alagal R, Aldera H, et al. Ellagic acid prevents streptozotocin-induced hippocampal damage and memory loss in rats by stimulating Nrf2 and nuclear factor-κB, and activating insulin receptor substrate/PI3K/Akt axis. Journal of Physiology and Pharmacology: an Official Journal of the Polish Physiological Society. 2021;72(4).

33. Brum GF, Rosa HZ, Rossato DR, Rosa JLO, Metz VG, Milanesi LH, et al. Binge and Subchronic Exposure to Ketamine Promote Memory Impairments and Damages in the Hippocampus and Peripheral Tissues in Rats: Gallic Acid Protective Effects. Neurotox Res. 2020;38(2):274-86.

34. Kumar RS, Narayanan SN, Nayak S. Ascorbic acid protects against restraint stress-induced memory deficits in Wistar rats. Clinics (Sao Paulo). 2009;64(12):1211-7.

35. Rivera F, Urbanavicius J, Gervaz E, Morquio A, Dajas F. Some aspects of thein vivo neuroprotective capacity of flavonoids: bioavailability and structure-activity relationship. Neurotoxicity research. 2004;6(7):543-53.

36. Luo Y, Cui HX, Jia A, Jia SS, Yuan K. The Protective Effect of the Total Flavonoids of Abelmoschus esculentus L. Flowers on Transient Cerebral Ischemia-Reperfusion Injury Is due to Activation of the Nrf2-ARE Pathway. Oxid Med Cell Longev. 2018;2018:8987173.

37. Burguete MC, Torregrosa Pérez‐Asensio G, Fernando J, Castelló‐Ruiz M, Salom Gil JB, José V, Alborch E. Dietary phytoestrogens improve stroke outcome after transient focal cerebral ischemia in rats. European Journal of Neuroscience. 2006;23(3):703-10.

38. Aggarwal A, Gaur V, Kumar A. Nitric oxide mechanism in the protective effect of naringin against post-stroke depression (PSD) in mice. Life sciences. 2010;86(25-26):928-35.

39. Shin WH, Park SJ, Kim EJ. Protective effect of anthocyanins in middle cerebral artery occlusion and reperfusion model of cerebral ischemia in rats. Life Sci. 2006;79(2):130-7.

40. Cai Y, Li X, Pan Z, Zhu Y, Tuo J, Meng Q, et al. Anthocyanin ameliorates hypoxia and ischemia induced inflammation and apoptosis by increasing autophagic flux in SH-SY5Y cells. Eur J Pharmacol. 2020;883:173360.

41. Min J, Yu SW, Baek SH, Nair KM, Bae ON, Bhatt A, et al. Neuroprotective effect of cyanidin-3-O-glucoside anthocyanin in mice with focal cerebral ischemia. Neurosci Lett. 2011;500(3):157-61.

42. Al-Majed AA, Al-Omar FA, Nagi MN. Neuroprotective effects of thymoquinone against transient forebrain ischemia in the rat hippocampus. Eur J Pharmacol. 2006;543(1-3):40-7.

43. Ozacmak VH, Sayan H, Cetin A, Akyildiz-Igdem A. AT1 receptor blocker candesartan-induced attenuation of brain injury of rats subjected to chronic cerebral hypoperfusion. Neurochem Res. 2007;32(8):1314-21.

44. Wang Y, Wu Y, Liang C, Tan R, Tan L, Tan R. Pharmacodynamic Effect of Ellagic Acid on Ameliorating Cerebral Ischemia/Reperfusion Injury. Pharmacology. 2019;104(5-6):320-31.

45. Zhang DL, Zhang YT, Yin JJ, Zhao BL. Oral administration of Crataegus flavonoids protects against ischemia/reperfusion brain damage in gerbils. Journal of neurochemistry. 2004;90(1):211-9.

46. Ryou MG, Liu R, Ren M, Sun J, Mallet RT, Yang SH. Pyruvate protects the brain against ischemia-reperfusion injury by activating the erythropoietin signaling pathway. Stroke. 2012;43(4):1101-7.

47. Berthet C, Lei H, Thevenet J, Gruetter R, Magistretti PJ, Hirt L. Neuroprotective role of lactate after cerebral ischemia. J Cereb Blood Flow Metab. 2009;29(11):1780-9.

48. Sun J, Li YZ, Ding YH, Wang J, Geng J, Yang H, et al. Neuroprotective effects of gallic acid against hypoxia/reoxygenation-induced mitochondrial dysfunctions in vitro and cerebral ischemia/reperfusion injury in vivo. Brain Res. 2014;1589:126-39.

49. Kodama N, Sasaki T, Kawakami M, Sato M, Asari J. Cisternal irrigation therapy with urokinase and ascorbic acid for prevention of vasospasm after aneurysmal subarachnoid hemorrhage. Outcome in 217 patients. Surg Neurol. 2000;53(2):110-7; discussion 7-8.

50. Kodama N, Matsumoto M, Sasaki T, Konno Y, Sato T. Cisternal irrigation therapy with urokinase and ascorbic acid for prevention of vasospasm. Cerebral Vasospasm: Springer; 2001. p. 171-4.

51. Satoh A, Sugiyama T, Ooigawa H, Nakajima H, Ogura T, Neki H, et al. Prevention of symptomatic vasospasm by continuous cisternal irrigation with mock-CSF containing ascorbic acid and Mg(2+). Acta Neurochir Suppl. 2010;107:115-8.

52. Lamport DJ, Pal D, Macready AL, Barbosa-Boucas S, Fletcher JM, Williams CM, et al. The effects of flavanone-rich citrus juice on cognitive function and cerebral blood flow: an acute, randomised, placebo-controlled cross-over trial in healthy, young adults. British journal of nutrition. 2016;116(12):2160-8.

53. Bowtell JL, Aboo-Bakkar Z, Conway ME, Adlam A-LR, Fulford J. Enhanced task-related brain activation and resting perfusion in healthy older adults after chronic blueberry supplementation. Applied Physiology, Nutrition, and Metabolism. 2017;42(7):773-9.

54. Palachai N, Wattanathorn J, Muchimapura S, Thukham-Mee W. Phytosome Loading the Combined Extract of Mulberry Fruit and Ginger Protects against Cerebral Ischemia in Metabolic Syndrome Rats. Oxid Med Cell Longev. 2020;2020:5305437.

55. Varshosaz J, Taymouri S, Pardakhty A, Asadi-Shekaari M, Babaee A. Niosomes of ascorbic acid and α-tocopherol in the cerebral ischemia-reperfusion model in male rats. (2314-6141 (Electronic)).

56. Choudhary N, Bijjem KR, Kalia AN. Antiepileptic potential of flavonoids fraction from the leaves of Anisomeles malabarica. J Ethnopharmacol. 2011;135(2):238-42.

57. Orhan N, Deliorman Orhan D, Aslan M, Sukuroglu M, Orhan IE. UPLC-TOF-MS analysis of Galium spurium towards its neuroprotective and anticonvulsant activities. J Ethnopharmacol. 2012;141(1):220-7.

58. Guo J, Xue C, Duan JA, Qian D, Tang Y, You Y. Anticonvulsant, antidepressant-like activity of Abelmoschus manihot ethanol extract and its potential active components in vivo. Phytomedicine. 2011;18(14):1250-4.

59. Jager AK, Krydsfeldt K, Rasmussen HB. Bioassay-guided isolation of apigenin with GABA-benzodiazepine activity from Tanacetum parthenium. Phytother Res. 2009;23(11):1642-4.

60. Abbasi E, Nassiri-Asl M, Shafeei M, Sheikhi M. Neuroprotective effects of vitexin, a flavonoid, on pentylenetetrazole-induced seizure in rats. Chem Biol Drug Des. 2012;80(2):274-8.

61. Lin TY, Lu CW, Wang CC, Lu JF, Wang SJ. Hispidulin inhibits the release of glutamate in rat cerebrocortical nerve terminals. Toxicol Appl Pharmacol. 2012;263(2):233-43.

62. Wang Z, Jiang X. Flavonoid-rich extract of Polygonum capitatum attenuates high-fat diet–induced atherosclerosis development and inflammatory and oxidative stress in hyperlipidemia rats. European Journal of Inflammation. 2018;16.

63. Plotnikov M, Aliev O, Maslov MJ, Vasiliev A, Tjukavkina N. Correction of the high blood viscosity syndrome by a mixture of diquertin and ascorbic acid in vitro and in vivo. Phytotherapy Research. 2003;17(3):276-8.

64. Park SJ, Kim DH, Jung JM, Kim JM, Cai M, Liu X, et al. The ameliorating effects of stigmasterol on scopolamine-induced memory impairments in mice. Eur J Pharmacol. 2012;676(1-3):64-70.

65. Reeta KH, Mehla J, Gupta YK. Curcumin is protective against phenytoin-induced cognitive impairment and oxidative stress in rats. Brain Res. 2009;1301:52-60.

66. Mansouri MT, Farbood Y, Sameri MJ, Sarkaki A, Naghizadeh B, Rafeirad M. Neuroprotective effects of oral gallic acid against oxidative stress induced by 6-hydroxydopamine in rats. Food chemistry. 2013;138(2-3):1028-33.

67. Mansouri MT, Farbood Y, Naghizadeh B, Shabani S, Mirshekar MA, Sarkaki A. Beneficial effects of ellagic acid against animal models of scopolamine-and diazepam-induced cognitive impairments. Pharmaceutical biology. 2016;54(10):1947-53.

68. Uzar E, Alp H, Cevik MU, Fırat U, Evliyaoglu O, Tufek A, et al. Ellagic acid attenuates oxidative stress on brain and sciatic nerve and improves histopathology of brain in streptozotocin-induced diabetic rats. Neurological Sciences. 2012;33(3):567-74.

69. Rafieirad M, GHASEMZADEH DK. Effect of ellagic acid on oxidative stress duo to brain ischemia/hypoperfusion in male rat. 2014.

70. Farbood Y, Sarkaki A, Dianat M, Khodadadi A, Haddad MK, Mashhadizadeh S. Ellagic acid prevents cognitive and hippocampal long-term potentiation deficits and brain inflammation in rat with traumatic brain injury. Life Sciences. 2015;124:120-7.

71. Hartman RE, Shah A, Fagan AM, Schwetye KE, Parsadanian M, Schulman RN, et al. Pomegranate juice decreases amyloid load and improves behavior in a mouse model of Alzheimer's disease. Neurobiology of disease. 2006;24(3):506-15.

72. Feng Y, Yang SG, Du XT, Zhang X, Sun XX, Zhao M, et al. Ellagic acid promotes Abeta42 fibrillization and inhibits Abeta42-induced neurotoxicity. Biochem Biophys Res Commun. 2009;390(4):1250-4.

73. Pavlica S, Gebhardt R. Protective effects of ellagic and chlorogenic acids against oxidative stress in PC12 cells. Free radical research. 2005;39(12):1377-90.

74. Hung K-C, Huang H-J, Wang Y-T, Lin AM-Y. Baicalein attenuates α-synuclein aggregation, inflammasome activation and autophagy in the MPP+-treated nigrostriatal dopaminergic system in vivo. Journal of ethnopharmacology. 2016;194:522-9.

75. Cheng Y, He G, Mu X, Zhang T, Li X, Hu J, et al. Neuroprotective effect of baicalein against MPTP neurotoxicity: behavioral, biochemical and immunohistochemical profile. Neuroscience Letters. 2008;441(1):16-20.

76. Zhang X, Du L, Zhang W, Yang Y, Zhou Q, Du G. Therapeutic effects of baicalein on rotenone-induced Parkinson’s disease through protecting mitochondrial function and biogenesis. Scientific reports. 2017;7(1):1-14.

77. Anusha C, Sumathi T, Joseph LD. Protective role of apigenin on rotenone induced rat model of Parkinson's disease: Suppression of neuroinflammation and oxidative stress mediated apoptosis. Chemico-biological interactions. 2017;269:67-79.

78. Goes AT, Jesse CR, Antunes MS, Ladd FVL, Ladd AAL, Luchese C, et al. Protective role of chrysin on 6-hydroxydopamine-induced neurodegeneration a mouse model of Parkinson's disease: Involvement of neuroinflammation and neurotrophins. Chemico-biological interactions. 2018;279:111-20.

79. Jeong KH, Jeon M-T, Kim HD, Jung UJ, Jang MC, Chu JW, et al. Nobiletin protects dopaminergic neurons in the 1-methyl-4-phenylpyridinium-treated rat model of Parkinson's disease. Journal of medicinal food. 2015;18(4):409-14.

80. Lee KM, Lee Y, Chun HJ, Kim AH, Kim JY, Lee JY, et al. Neuroprotective and anti‐inflammatory effects of morin in a murine model of Parkinson's disease. Journal of neuroscience research. 2016;94(10):865-78.

81. Lv C, Hong T, Yang Z, Zhang Y, Wang L, Dong M, et al. Effect of quercetin in the 1-methyl-4-phenyl-1, 2, 3, 6-tetrahydropyridine-induced mouse model of Parkinson's disease. Evidence-Based Complementary and Alternative Medicine. 2012;2012.

82. Khan M, Raza SS, Javed H, Ahmad A, Khan A, Islam F, et al. Rutin protects dopaminergic neurons from oxidative stress in an animal model of Parkinson’s disease. Neurotoxicity research. 2012;22(1):1-15.

83. Zbarsky V, Datla KP, Parkar S, Rai DK, Aruoma OI, Dexter DT. Neuroprotective properties of the natural phenolic antioxidants curcumin and naringenin but not quercetin and fisetin in a 6-OHDA model of Parkinson's disease. Free radical research. 2005;39(10):1119-25.

84. Mani S, Sekar S, Barathidasan R, Manivasagam T, Thenmozhi AJ, Sevanan M, et al. Naringenin decreases α-synuclein expression and neuroinflammation in MPTP-induced Parkinson’s disease model in mice. Neurotoxicity Research. 2018;33(3):656-70.

85. Kiasalari Z, Khalili M, Baluchnejadmojarad T, Roghani M. Protective Effect of Oral Hesperetin Against Unilateral Striatal 6-Hydroxydopamine Damage in the Rat. Neurochem Res. 2016;41(5):1065-72.

86. Kim HD, Jeong KH, Jung UJ, Kim SR. Naringin treatment induces neuroprotective effects in a mouse model of Parkinson's disease in vivo, but not enough to restore the lesioned dopaminergic system. J Nutr Biochem. 2016;28:140-6.

87. Antunes MS, Goes AT, Boeira SP, Prigol M, Jesse CR. Protective effect of hesperidin in a model of Parkinson's disease induced by 6-hydroxydopamine in aged mice. Nutrition. 2014;30(11-12):1415-22.

88. Shengkai D, Qianqian L, Yazhen S. The Effects and Regulatory Mechanism of Flavonoids from Stems and Leaves of Scutellaria baicalensis Georgi in Promoting Neurogenesis and Improving Memory Impairment Mediated by the BDNF-ERK-CREB Signaling Pathway in Rats. CNS Neurol Disord Drug Targets. 2022;21(4):354-66.

89. Xu Z, Chen S, Li X, Luo G, Li L, Le W. Neuroprotective effects of (-)-epigallocatechin-3-gallate in a transgenic mouse model of amyotrophic lateral sclerosis. Neurochem Res. 2006;31(10):1263-9.

90. Qureshi AA, Tan X, Reis JC, Badr MZ, Papasian CJ, Morrison DC, et al. Suppression of nitric oxide induction and pro-inflammatory cytokines by novel proteasome inhibitors in various experimental models. Lipids in health and disease. 2011;10(1):1-25.

91. Yammine A, Zarrouk A, Nury T, Vejux A, Latruffe N, Vervandier-Fasseur D, et al. Prevention by Dietary Polyphenols (Resveratrol, Quercetin, Apigenin) Against 7-Ketocholesterol-Induced Oxiapoptophagy in Neuronal N2a Cells: Potential Interest for the Treatment of Neurodegenerative and Age-Related Diseases. Cells. 2020;9(11):2346.

92. Xiong L-G, Chen Y-J, Tong J-W, Gong Y-S, Huang J-A, Liu Z-H. Epigallocatechin-3-gallate promotes healthy lifespan through mitohormesis during early-to-mid adulthood in Caenorhabditis elegans. Redox biology. 2018;14:305-15.

93. Sung B, Chung JW, Bae HR, Choi JS, Kim CM, Kim ND. Humulus japonicus extract exhibits antioxidative and anti-aging effects via modulation of the AMPK-SIRT1 pathway. Exp Ther Med. 2015;9(5):1819-26.

94. Giampieri F, Alvarez-Suarez JM, Cordero MD, Gasparrini M, Forbes-Hernandez TY, Afrin S, et al. Strawberry consumption improves aging-associated impairments, mitochondrial biogenesis and functionality through the AMP-activated protein kinase signaling cascade. Food chemistry. 2017;234:464-71.

95. Lewinska A, Adamczyk-Grochala J, Bloniarz D, Olszowka J, Kulpa-Greszta M, Litwinienko G, et al. AMPK-mediated senolytic and senostatic activity of quercetin surface functionalized Fe(3)O(4) nanoparticles during oxidant-induced senescence in human fibroblasts. Redox Biol. 2020;28:101337.
